# New Difunctional Derivatives of Betulin: Preparation, Characterization and Antiproliferative Potential

**DOI:** 10.3390/molecules30030611

**Published:** 2025-01-30

**Authors:** Elwira Chrobak, Marta Świtalska, Joanna Wietrzyk, Ewa Bębenek

**Affiliations:** 1 Department of Organic Chemistry, Faculty of Pharmaceutical Sciences in Sosnowiec, Medical University of Silesia in Katowice, 4 Jagiellońska Str., 41-200 Sosnowiec, Poland; 2 Hirszfeld Institute of Immunology and Experimental Therapy, Polish Academy of Sciences, 12 Rudolfa Weigla Str., 53-114 Wrocław, Poland; marta.switalska@hirszfeld.pl (M.Ś.); joanna.wietrzyk@hirszfeld.pl (J.W.)

**Keywords:** betulin, triterpenes, antiproliferative activity, apoptosis

## Abstract

Biologically active compounds of natural origin, such as betulin, are a source of obtaining new medicinal substances. The presence of chemically active hydroxyl groups in the betulin structure at C-3 and C-28 positions enables esterification with dicarboxylic acid anhydrides or carboxylic acids. As a result of a four-step synthesis, difunctional betulin derivatives were obtained, which were evaluated for their antiproliferative activity against the following human cell lines: leukemia (MV4-11), (A549), breast cancer (MCF-7), prostate adenocarcinoma (PC-3), colon cancer (HCT116), pancreatic cancer (MiaPaca-2), and melanoma (Hs294T). The target 3-carboxyacyl-28-alkynyloyl betulin derivatives showed significant antiproliferative activity against MV4-11 cells. For 3-carboxyacylbetulins and their selected alkynyl derivatives, studies to investigate the effect on the cell cycle and apoptosis process, as well as drug similarity analysis, were performed.

## 1. Introduction

Despite the development of the new treatment strategies, cancer is still the main cause of death in the world. Commonly used treatments such as radiotherapy, chemotherapy, and surgical removal do not always provide the ability to defeat cancer cells. In addition, available treatment regimens are often characterized by insufficient selectivity. Currently used anticancer drugs are based on the use of compounds of natural origin like alkaloids, taxanes, lignans, saponins, terpenes, and flavonoids. The anticancer effect of natural compounds may be related to the inhibition of proteins and enzymes that activate cancer cells, the initiation of DNA (deoxyribonucleic acid) repair mechanisms, the activation of the formation of protective enzymes (caspase-3, -7, -8, -9, -10, and -12) or the induction of antioxidant activity. Compounds of natural origin often act selectively on cancer cells while having no cytotoxic effect on normal cells. Moreover, relatively simple modifications of the chemical structure of natural compounds also help us with obtaining new anticancer drugs with a similar or more favorable biological action profile than the parent compounds [1,2].

Betulin, obtained from the bark of various birch species, has been the subject of many studies for several decades aimed at using its pharmacological potential in therapy. It is characterized by a wide spectrum of biological activity, while at the same time containing reactive groups in its structure that allow new derivatives to be obtained [3,4,5,6,7,8,9,10,11]. There are many strategies that can be used for the functionalization of the betulin molecule.

It has been shown that triterpene hemiesters obtained via the esterification of free hydroxyl groups with dicarboxylic acid anhydrides may be characterized by reduced lipophilicity and increased bioavailability compared to the parent molecules [12,13]. Such a modification of the structure may also cause an increase in biological activity or cause the action to be revealed in a different direction. Among the most well-known derivatives of this type, it is worth mentioning 3-O-(3′,3′-dimethylsuccinyl)betulinic acid (bevirimat) with anti-HIV (anti-human immunodeficiency virus) activity [14]. However, few works investigate the effect of such a substituent in the betulin molecule on anticancer activity [13,15,16].

The growing interest in alkyne derivatives results from the possibility of using them in multicomponent reactions (including with carboxylic acids, amines, ketones, aldehydes, isocyanides, sulfur-containing compounds, and azides), leading to the formation of molecules with diverse chemical structures and biological activity [17]. The role of the alkynyl substituent as an important functional group in medicinal chemistry is confirmed by a large number (about 30) of approved drugs that contain it and are characterized by different directions of action [18,19]. Numerous studies, including those on betulin derivatives, have shown that the introduction of an alkynyl substituent may have a beneficial effect on the anticancer activity of the obtained molecule, and they may also enable further beneficial chemical modifications of the derivatives obtained in this way [20,21,22,23,24].

**Figure 1:**
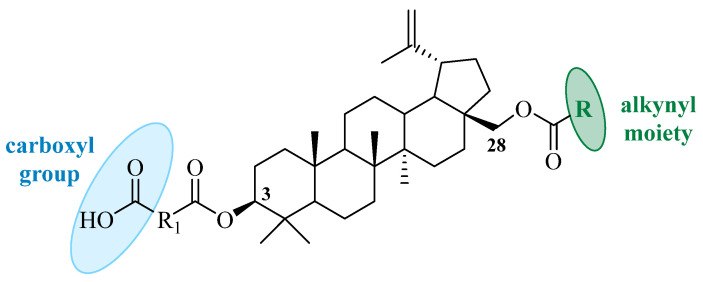
Strategy for the functionalization of the betulin molecule.

Taking into account the above reports, the aim of this work was to determine what effect can be obtained by combining these two strategies centered around the functionalization of the betulin molecule (Figure 1). For this purpose, new betulin derivatives containing a 3′,3′-dimethylsuccinic, succinic, or 3′,3′-dimethylglutaric substituent at the C-3 position and alkynyloyl substituents at the C-28 position differ in the number of carbon atoms, ranging from three to five, and the position of the triple bond in the carbon chain.

Seven human cancer cell lines were selected to investigate the antiproliferative potential of synthesized 3,28-disubstituted betulin derivatives. The obtained results allowed for the analysis of structure–activity relationships and for the study of the mechanism of action for the most active molecules.

## 2. Results and Discussion

### 2.1. Chemistry

**Scheme 1:**
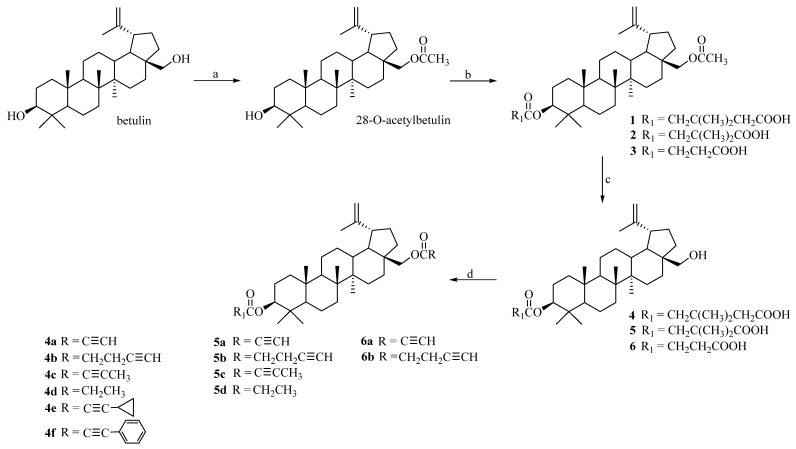
Synthesis scheme of difunctional derivatives of betulin **4a**–**4f**, **5a**–**5d**, **6a**, and **6b**. Reagents and conditions: (a) (CH_3_CO)_2_O, pyridine, room temperature, 24 h; (b) dicarboxylic acid anhydride, DMAP (4-dimethylaminopyridine), pyridine, 160 °C, 1 h, microwave; (c) NaOH, THF/CH_3_OH/H_2_O, room temperature, 1.5 h; (d) carboxylic acid, DCC *(N*,*N*′-dicyclohexylcarbodiimide), DMAP, CH_2_Cl_2_, from −5 °C to room temperature, 29 h.

The first stage of functionalization of the betulin molecule was carried out to obtain 3-carboxyacyl derivatives **4**–**6**, to which the introduction of ester groups was then also planned at the C-28 position. Compounds **4**–**6** were obtained from betulin in a three-step procedure involving the protection of the hydroxyl group at the C-28 position with an acetyl group (Scheme 1, step a), the esterification of the obtained 28-O-acetylbetulin with dicarboxylic acid anhydrides (Scheme 1, step b), and finally the selective basic hydrolysis of the acetyl protecting group at the C-28 position (Scheme 1, step c).

Betulin, which is the starting compound for the synthesis, is characterized by the presence of two hydroxyl groups, where the primary hydroxyl group at the C-28 position is much more reactive in the esterification reaction than the secondary hydroxyl group at the C-3 position. For this reason, before introducing the carboxyacyl substituent at C-3, the hydroxyl group at C-28 had to be protected. In the literature, descriptions of the synthesis of these compounds using various protecting agents can be found. An example is the formation of esters, which is carried out by formulating a reaction with tert-butyldimethylsilyl chloride (TBDMSCl), deprotecting them using HF-pyridine or ethers via a reaction with triphenylmethyl chloride, and then deprotecting them using pyridinium p-toluenesulfonate (PPTS) or zinc chloride (ZnCl_2_) in tetrahydrofuran (THF) [25,26,27].

In the presented work, the protection of the primary hydroxyl group of betulin was carried out using selective acetylation with acetic anhydride. This can be achieved using a molar equivalent of acetic anhydride in the presence of catalytic amounts of 4-dimethylaminopyridine (DMAP) in pyridine solution. Mainly 28-O-acetylbetulin and small amounts of 3,28-O,O-diacetylbetulin are obtained, which can then be separated from each other using column chromatography on the silica gel [28].

The esterification of the hydroxyl group at the C-3 position with dicarboxylic acid anhydrides catalyzed by DMAP requires the use of high temperatures. In order to increase the yield and shorten the reaction time, the microwave-assisted organic synthesis technique was used [29]. Two anhydrides containing geminal methyl substituents and differing in the number of carbon atoms in the chain were selected for the reaction, and, for comparison, an anhydride without chain branches was added [30]. In the reaction of 28-O-acetylbetulin with 3,3-dimethylglutaric, 2,2-dimethylsuccinic and succinic acid anhydrides were carried out using this method, and compounds **1**–**3** were obtained with yields in the range of 68–86%.

Due to the asymmetric structure of 2,2-dimethylsuccinyl anhydride, two isomeric esterification products can be formed at the C-3 position, 28-acetyl-3-(3′,3′-dimethylsuccinyl)betulin and 28-acetyl-3-(2′,2′-dimethylsuccinyl)betulin (Figure S1, Supplementary Materials). The main product isolated from the reaction mixture obtained in this work is a compound with a 3′,3′-dimethylsuccinyl substituent (compound **2**). The acylation reaction occurring at the C-4 carbon of 2,2-dimethylsuccinyl anhydride is characterized by less steric hindrance. The presence of two methyl groups at the C-2 carbon of the anhydride hinders the reaction at its C-1 carbon. This result is consistent with the previously described reaction of 2,2-dimethylsuccinyl anhydride with betulinic acid performed under similar conditions, where the 3′,3′-dimethylsuccinic acid derivative (bevirimat) was obtained in a 62–81% yield [31]. The structure of derivative **2**, which is a substrate for further synthesis steps, was confirmed based on the analysis of two-dimensional NMR (nuclear magnetic resonance) spectra, which are included in the Supplementary Materials. In the HMBC (heteronuclear multiple-bond correlation) spectrum, a strong correlation of geminal protons at the C-2′ with the carboxyl carbon atom (C-4′) and the carbon of the ester group (C-1′) was observed. At the same time, no correlation of protons of two methyl groups (at the C-3′) with the carbon atom of the ester group (C-1′) was found. This result confirms that compound **2** is 28-acetyl-3-(3′,3′-dimethylsuccinyl)betulin. The reactions leading to the transformation of compound **2** into the target derivatives **5** and **5a**–**5d** occurred at the C-28 position and did not affect the carboxyacyl substituent at the C-3 position.

The hydrolysis reaction of the acetyl protecting group was carried out in an alkaline medium, (in a solution of sodium hydroxide in a mixture of water, tetrahydrofuran, and methanol in a volume ratio of 1:2:1) [32]. The ester group at the C-28 position is more susceptible to hydrolysis than the group at the C-3 position, so by carefully controlling the reaction time, it was possible to obtain products **4**–**6** in a good yield of 56–82%.

The most important step of the planned synthetic path was the introduction of alkynyl substituents to the 3-carboxyacylbetulin molecules **4**–**6**. Additionally, to compare the effect of such a functional group containing a triple carbon–carbon bond, two derivatives of propanoic acid **4d** and **5d** were also synthesized.

### 2.2. Antiproliferative Activity

So far, 3-carboxyacylbetulins containing 3′,3′-dimethylsuccinic, succinic, or 3′,3′-dimethylglutaric substituents have been tested for anti-HIV activity [25,26,27]. Shintyapina et al. reported the effect of 3,28-disuccinylbetulin and 3-acetyl-28-succinylbetulin on the growth and apoptosis of human leukemia (MT-4, MOLT-4, and CEM) and hepatocellular carcinoma (Hep G2) cells. The activity of these two derivatives against the tested cells was in the range of 17.11–108.89 µM. Succinyl derivatives induced apoptosis in all tested cell lines and their activity was similar to that of betulinic acid [15].

The alkynyl derivatives of pentacyclic triterpenes of the lupane type described in the literature have significant biological activity, including antiproliferative, hepatoprotective, and antiviral effects [33,34,35]. In our work, we attempted to find structurally new, promising betulin derivatives based on the concept of combining a carboxyacyl and alkynyl group in one molecule. Reagents (acid anhydrides and carboxylic acids) with diverse structures were used in the synthesis to conduct a preliminary assessment of the structure–activity relationship for these new combinations.

All eighteen synthesized compounds **1**–**6b** were subjected to an evaluation of antiproliferative activity. Human cell lines of various cancers such as biphenotypic myelomonocytic leukemia (MV4-11), breast cancer (MCF-7), lung cancer (A549), prostate cancer (PC-3), pancreatic cancer (MiaPaca-2), colon cancer (HCT116), and melanoma (Hs249T) were selected for the studies. The cytotoxicity of the synthesized compounds was tested on a non-tumorigenic epithelial cell line MCF-10A. The obtained results are presented in Table 1.

As can be seen, the initial betulin molecule shows relatively weak activity against the tested cell lines [IC_50_ (inhibitory concentration 50%) in the range of 14–144 µM] and esterification with acetic anhydride, leading to the formation of 28-O-acetylbetulin and resulting in a further decrease in activity. In the case of the MiaPaca-1 line, the activity of 28-O-acetylbetulin is slightly higher but still very low in relation to the derivatives that are the subject of the work.

Considering the antiproliferative activity of the carboxyacyl derivatives of 28-acetylbetulin (**1**–**3**), the highest activity is shown by compound **1** containing a 3′,3′-dimethylglutaric substituent at C-3. Shortening the chain (3′,3′-dimethylsuccinyl derivative **2**) weakens the activity of the obtained derivative **2**. The presence of an unbranched succinyl substituent (compound **3**) causes a further decrease in activity, with the exception of line MV-4-11.

A similar pattern can be observed for hydrolysis products **4**–**6**. For all cell lines used in the study, the most active is the 3′,3′-dimethylglutarate derivative (compound **4**, 3.76–24.45 µM). Derivatives with a shorter carbon chain of the substituent at C-3 are characterized by significantly lower activity in the range of 6.45 µM to 38.13 µM, while for cell lines MV4-11, A549, MCF-7, and PC-3, compound **6** (with a succinyl substituent) showed slightly higher activity. In relation to the other lines, the branched compound **5** (with a dimethylsuccinic substituent) is more active.

The subsequent products of betulin molecule modification, consisting in introducing alkynyl groups containing a terminal triple carbon–carbon bond (compounds of series “a” and “b”) or internal (“c”, “e”, and “f”) into the C-28 position, were subjected to an evaluation of antiproliferative activity. For comparison, derivatives not containing a multiple bond (compounds **4d** and **5d**) were also tested.

Considering the derivatives with a terminal triple bond, it can be seen that the introduction of a propynoyl group causes a significant increase in activity against most of the tested lines, except for lung cancer A549 cells (compounds **4a**, **5a**, and **6a**) and breast cancer MCF-7 (compound **4a**). Csuk et al. described the synthesis and anticancer activity of betulin derivatives containing a carbonyl group at C-28 and a short substituent with a terminal triple bond [37]. In relation to the cell lines also used in our study (A549, HCT116, and MCF-7), these compounds showed high activity in the range of 0.35–18.7 µM, which confirms our results.

Carbon chain extension (pentynoyl derivatives **4b**, **5b**, and **6b**) causes a decrease in activity against the MV4-11 leukemia line. The activity of compounds with an internal C≡C bond depends on the size of the substituent at the end of the chain. As previously shown by Csuk et al., the presence of a phenyl ring at this site can lead to a decrease in or even loss of activity [38]. Considering the A549, HTC116, and MCF-7 cell lines, our results confirm this effect. The phenyl derivative **4f** does not show activity against the A549 and HCT116 line, while its IC_50_ value against the MCF-7 line is determined at 130.71 µM. Comparing the activity of the remaining derivatives with an internal triple bond, a decrease in activity is observed when the terminal methyl group (compound **4c**) is replaced by a more spatially expanded cyclopropyl group (compound **4e**).

The saturated analogue of the propynoyl group at position C-28 (compound **4d**) is characterized by lower activity than **4a** against the tested cancer cells, except for the A549 line.

By analyzing the activity of the target difunctional betulins, it can be stated that branching the carbon chain of the acyl substituent at position C-3 of propynoyl derivatives (**4a**, **5a**, and **6a**) causes an increase in activity against MV4-11 cells.

Based on the IC_50_ values of the compounds towards the tumor lines and the normal line MCF-10A, the selectivity index (SI) was calculated (Table 2). Most of the compounds showed selectivity of action (SI > 1), and the highest SI coefficient value was calculated for the most active compound **4a**, especially towards the leukemia line MV4-11 (SI = 99), prostate cancer PC-3 (SI = 38.6), and melanoma Hs294T (SI = 67). Compound **6a** also showed a high selectivity of action towards the leukemia line MV4-11 (SI =19), prostate cancer PC-3 (SI =121), and melanoma Hs294T (SI = 14).

### 2.3. Cell Cycle Study

Apoptosis is a process that is essential for maintaining the proper balance between cell proliferation and death. The inhibition of this process causes uncontrolled cell division and is the cause of various diseases, mainly cancer. Current research on new anticancer drugs is often directed towards finding agents that induce apoptosis [39]. Terpenoids, including betulin and its derivatives, belong to this group [36,37,38,39,40,41,42,43].

**Figure 2:**
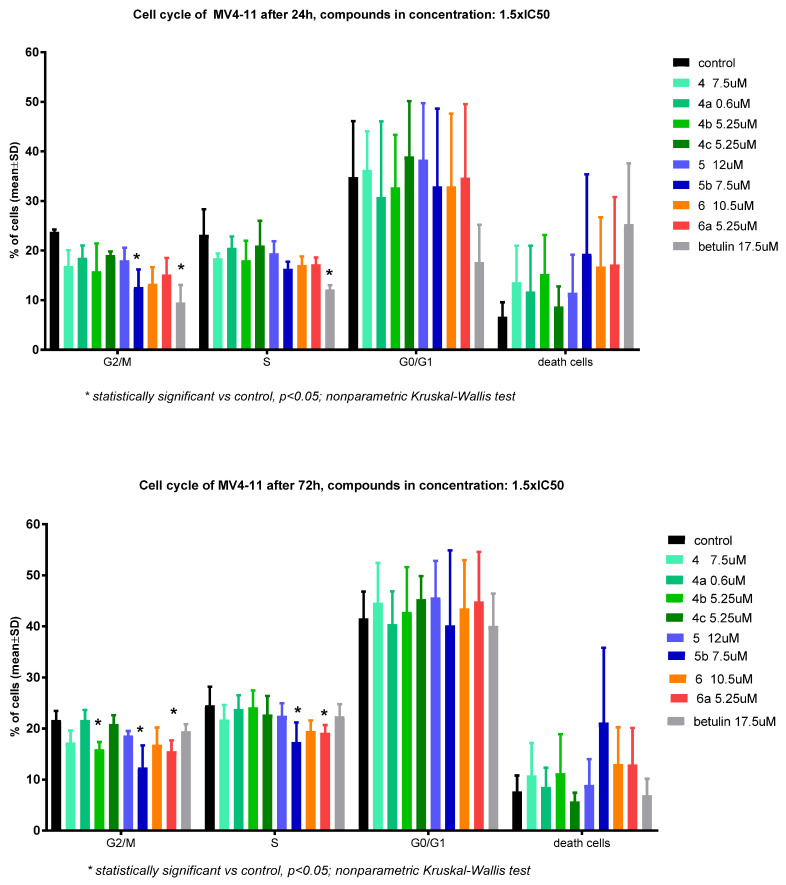
Cell cycle of MV4-11 cells after 24 h and 72 h of incubation with tested compounds.

MV4-11 cells were incubated for 24 h and 72 h with tested compounds and then cell cycle phases were determined using flow cytometry. Only **4b**, **5b**, and **6a** compounds had some influence on cell cycle. They statistically significant decreased the number of cells in the G2/M and S phases after 72 h of incubation and increased the number of death cells. After 24 h of incubation, only derivative **5b** and betulin statistically significantly lowered the number of cells in the G2/M phase and also betulin in the S and G0/G1 phases (Figure 2).

### 2.4. Apoptosis Determination via Annexin V Staining

**Figure 3:**
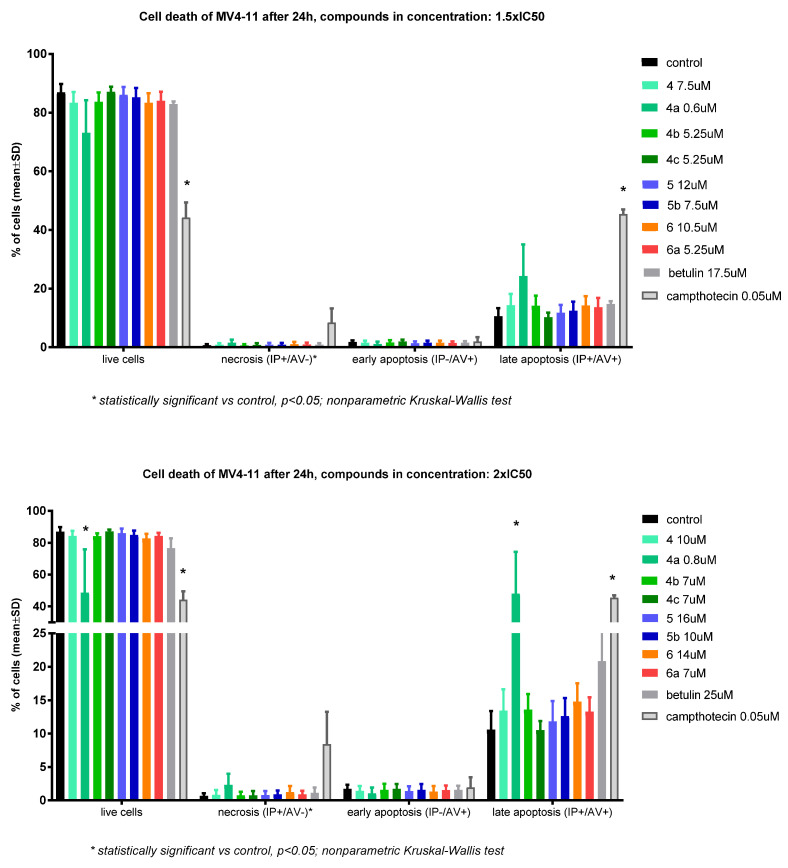
Cell death (apoptosis and necrosis) of MV4-11 cells after 24 h of incubation with the tested compounds.

**Figure 4:**
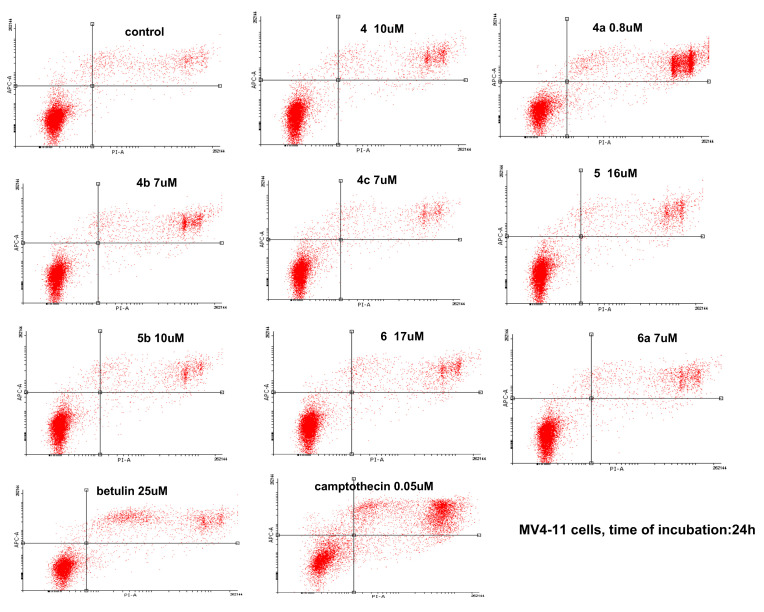
Cell death (apoptosis and necrosis) of MV4-11 cells after 24 h of incubation with the tested compounds. Dot plots were obtained from the flow cytometry. X-axis: PI fluorescence; Y- axis: APC fluorescence.

**Figure 5:**
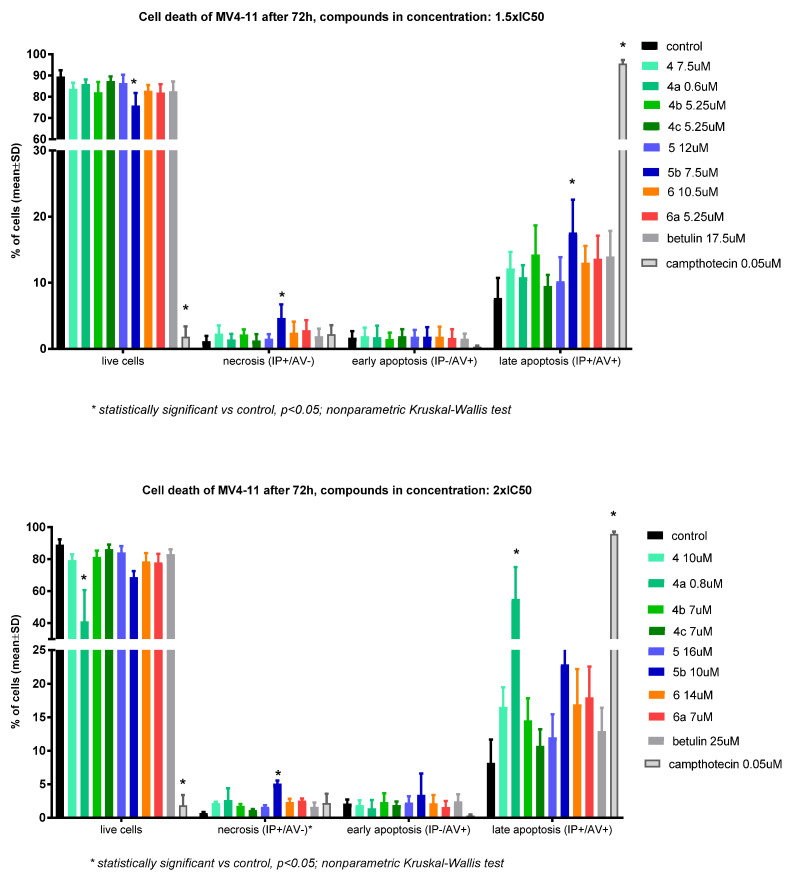
Cell death (apoptosis and necrosis) of MV4-11 cells after 72 h of incubation with the tested compounds.

**Figure 6:**
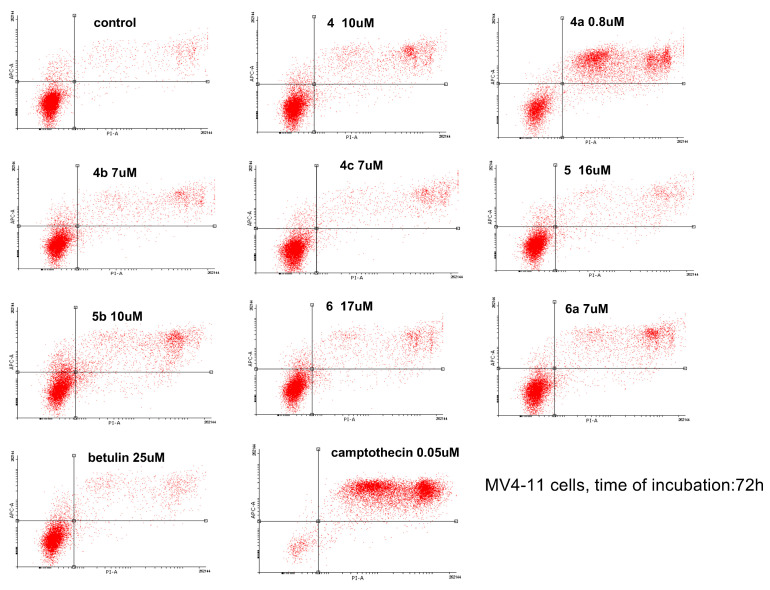
Cell death (apoptosis and necrosis) of MV4-11 cells after 72 h of incubation with the tested compounds. Dot plots were obtained from the flow cytometry. X-axis: PI fluorescence; Y- axis: APC fluorescence.

MV4-11 cells were incubated in 24 h and 72 h (Figure 3, Figure 4, Figure 5 and Figure 6) with tested compounds and camptothecin (as a positive control), and then apoptosis was determined using Annexin V and PI staining and the cells were analyzed using flow cytometry. The compounds were used in two concentrations. After 24 h of incubation, only derivative **4a** and camptothecin induced apoptosis. When leukemia cells were incubated with compounds (at higher concentrations) for longer (72 h), the induction of apoptosis was observed after the incubation of cells with **4**, **4a, 4b**, **5b**, **6**, and **6a** (the result was statistically significant for this compound). Compound **5b** also increased the number of necrotic cells.

### 2.5. Caspase-3/7 Activity Study

No caspase-3/7 activity was observed after 24 h and 72 h incubation of MV4-11 cells with tested compounds. In comparison to control cells, the activity of caspase-3 was observed to be only 5-fold higher in cells after being incubated with camptothecin, which was used as a positive control.

### 2.6. Similarity to Drugs

The drug likeness of chemical molecules is determined at an early stage of research in order to select substances with optimized physicochemical and pharmacokinetic properties that fall within the range of values determined for approved drugs. The evaluation of newly synthesized compounds is based on molecular descriptors such as molecular weight (MW), the number of hydrogen bond donors (HDs) and acceptors (HAs), topological polar surface area (TPSA), lipophilicity (log P), molar refractive index (MR), and the number of rotatable bonds (RBs). Several rules using molecular properties have been developed to predict the drug likeness of chemical compounds, namely Lipinski’s rule of five, Ghose’s rule, and Veber’s rule [44,45].

For compounds similar to orally active drugs, the rule proposed by Lipinski in 1997 recommends a certain molecular weight (MW ≤ 500), a certain number of hydrogen bond acceptors (HAs ≤ 10), a certain number of hydrogen bond donors (HDs ≤ 5), and a certain logarithm of the n-octanol/water partition coefficient log (P ≤ 5) [44,45]. Another rule defining drug candidates is the rule proposed by Veber. According to this rule, two parameters such as topological polar surface (TPSA ≤ 140 Å^2^) and the number of rotatable bonds (RBs ≤ 10) are selective criteria in the evaluation of compounds showing similarity to orally active drugs [46]. By analyzing the calculable physicochemical properties of various known drug classes, Ghose established guidelines according to which potential drugs should exhibit log P values from −0.4 to 5.6, molar refractive index (MR) values from 40 to 130, molecular weight (MW) values from 160 to 480, and a total number of atoms (nAtom) from 20 to 70 [47].

**Figure 7:**
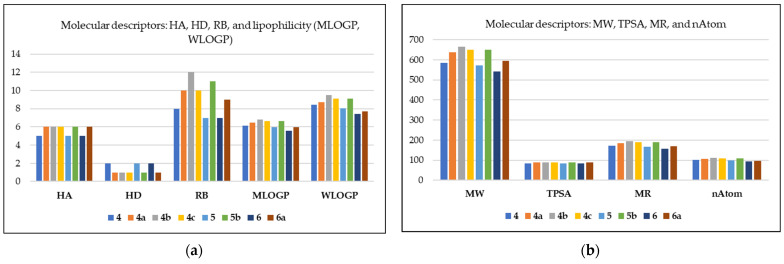
Molecular descriptors for derivatives **4**, **4a**–**c**, **5**, **5b**, **6**, and **6a**: (**a**) HAs—hydrogen bond acceptors, HDs—hydrogen bond donors, RBs—rotational bonds, and lipophilicity (MLOGP and WLOGP); (**b**) MW—molecular weight (g/mol), TPSA—topological polar surface area (Å^2^), MR—molar refractivity (cm^3^/mol), and number of atoms (nAtom).

To determine the similarity of drugs, eight derivatives (**4**, **4a**–**c**, **5**, **5b**, **6**, and **6a**) were selected, which had previously been subjected to cell cycle analysis and apoptosis studies. The physicochemical parameters of these compounds were calculated using the Swiss-sADME web server, and the obtained data are shown in Figure 7a,b.

All compounds show two violations of Lipinski’s rule regarding molar mass (MW in the range of 542.79 to 664.95 g/mol) and lipophilicity parameter values (MLOGP in the range of 5.60 to 6.82). Naturally derived drugs are the main source of oral drugs that do not meet Lipinski’s rule of five. The last two decades have seen an increase in the number of approved oral drugs with molecular weights above 500 [48]. Two derivatives **4b** and **5b** containing a pentynoyl substituent at C-28 position do not satisfy Veber’s rule due to exceeding the number of rotatable bonds (RBs) from 11 to 12. The remaining compounds **4**, **4a**, **4c**, **5**, **6**, and **6a** satisfy the criteria of Veber’s rule. Based on the values of calculated molecular parameters presented in Figure 7a,b, we can conclude that the analyzed derivatives do not satisfy Ghose’s rule (a violation of the MW, WLOGP, MR, and nAtom).

## 3. Materials and Methods

All reagents and solvents applied in the synthesis of new betulin derivatives come from a commercial source (Merck, Darmstadt, Germany). Reactions of 28-O-acetylbetulin with the corresponding carboxylic acid anhydrides were carried out in a microwave reactor (Discover SP-D, CEM Corporation, Matthews, NC, USA).

The identity and structure of the synthesized compounds were confirmed by determining the melting points and spectroscopic analysis. The melting points (mp) of all synthesized compounds were determined in an Electrothermal IA 9300 apparatus (Electrothermal, Rochford, UK) and uncorrected.

^1^H and ^13^C NMR (600 MHz and 150 MHz, respectively) spectra were obtained for samples dissolved in deuterated chloroform (CDCl_3_) using a Bruker Avance III 600 (Bruker Corporation, Billerica, MA, USA). The chemical shift scale (δ in ppm) was determined relative to the CDCl_3_ signal. HRMS spectra were recorded on a Bruker Impact II instrument (Bruker Corporation, Billerica, MA, USA) in the negative ion mode (APCI).

The progress of the reaction was monitored by conducting thin-layer chromatography on silica gel 60 254F plates (Merck, Darmstadt, Germany) in a mixture of dichloromethane and ethanol in appropriate volume proportions comparable to those of the eluent. The chromatograms were visualized by spraying the mixture with a 10% ethanolic solution of H_2_SO_4_ and then heating it to 100 °C. The purification of crude products was carried out via column chromatography, where the solid phase was silica gel 60 (0.063–0.200 mm, Merck, Darmstadt, Germany) and appropriate mixtures of dichloromethane and ethanol were used as the mobile phase.

### 3.1. General Experimental Procedures

**Figure 8:**
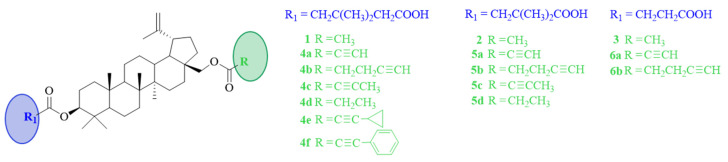
General formula of the new compounds synthesized in this work.

The general formula including all new 3,28-difunctional derivatives of betulin synthesized in this work is given in Figure 8.

### 3.2. Procedure for the Synthesis of Betulin Derivatives

#### 3.2.1. Procedure for the Preparation of 3-Carboxyacyl-28-O-Acetylbetulins **1-3**

Carboxyacyl derivatives of 28-O-acetylbetulin were obtained by reacting 730 mg of 28-O-acetylbetulin (1.5 mmol) with 7.5 mmol of the appropriate dicarboxylic acid anhydride (3,3-dimethylglutaric, 2,2-dimethylsuccinic or succinic). The reaction was carried out in a solution of 2.5 mL of pyridine and in the presence of 275 mg (2.25 mmol) of 4-dimethylaminopyridine (DMAP) as a catalyst. The tightly closed test tube containing the mixture was placed in a microwave reactor and heated at maximum power (300 W) for an hour at 160 °C. The reaction mixture was cooled and diluted with 20 mL of ethyl acetate and then transferred to a separatory funnel. The solution was shaken twice with 10 mL of 20% hydrochloric acid solution and then three times with 10 mL portions of water. The organic phase was collected and dried by adding anhydrous sodium sulfate (VI), before then being filtered and evaporated to dryness in a vacuum evaporator. The crude product was purified by conducting column chromatography on the silica gel. A mixture of dichloromethane and ethyl alcohol in a volume ratio of 40:1 was used as the eluent. Spectroscopic data for the obtained products **1**–**3** are provided in the Supplementary Materials.

#### 3.2.2. Procedure for the Preparation of 3-Carboxyacylbetulins **4-6**

Compounds **4**–**6** were obtained via the hydrolysis of 28-acetyl group of derivatives **1**–**3**. Then, 1.3 Mmol of the appropriate carboxyacyl derivative of 28-O-acetylbetulin (**1**–**3**) was placed in a reaction flask, and sodium hydroxide solution (321 mg of NaOH in a mixture of 14.5 mL of water, 14.5 mL of methanol, and 29 mL of tetrahydrofuran) was added. The reaction mixture was stirred with a magnetic stirrer for 80 min at room temperature. After this time, TLC analysis showed the completion of the hydrolysis reaction. The reaction mixture was transferred to a separatory funnel, 10% HCl solution was added until a weakly acidic reaction was obtained, and then it was extracted with 4 portions of 10 mL of dichloromethane. The organic phase was dried with anhydrous sodium sulfate (VI), filtered into a flask, and evaporated to dryness on a rotary evaporator. The remaining precipitate was purified by conducting column chromatography on the silica gel. A mixture of chloroform and ethyl alcohol in a ratio of 15:1 was used as the eluent.

The 3-carboxyacylbetulin derivatives **4**–**6** described earlier in the literature were obtained from betulin 28-O-triphenylmethyl ether, which was reacted with the appropriate anhydride in pyridine at reflux in the presence of DMAP. The protecting group at the C-28 (trityl group) was removed by refluxing with pyridinium p-toluenesulfonate (PPTS) in a mixture of dichloromethane and ethanol [26,27].

The spectroscopic data of compounds **4**–**6** were consistent with the literature data [25,26,27]. The melting points determined by us were as follows: for derivative **4**, mp 206–208 °C; for derivative **5**, mp 259–261 °C; and for derivative **6**, mp 253–255 °C (lit. mp 255.4–256.8 °C [25]).

#### 3.2.3. Procedure for Preparation of 3,28-Diacylbetulins **4a–f, 5a–5d, 6a**, and **6b**

To a mixture of 0.23 mmol of 3-carboxyacylbetulin (**4** or **5** or **6**) and 0.29 mmol of the appropriate acid (propynoic, 4-pentynoic, 2-butynoic, propanoic, 3-cyclopropyl-2-propynoic, or phenylpropynoic) in 3 mL of dichloromethane, cooled in a water–salt bath to −10 °C, a solution of 0.018 mmol (2 mg) of DMAP and 0.29 mmol (60 mg) of DCC in 2 mL of dichloromethane was added dropwise. The whole was stirred on a magnetic stirrer for 5 h in an ice bath, and then for 24 h at room temperature. When TLC analysis showed the absence of substrate, the reaction was terminated. The reaction mixture was filtered into a round-bottom flask, and then the solvent was evaporated on a rotary evaporator. The reaction products were purified using column chromatography (SiO_2_, chloroform/ethanol, 15:1, *v*/*v*). Spectroscopic data for the obtained products **4a**–**f**, **5a**–**5d**, **6a**, and **6b** are provided in the Supplementary Materials.

### 3.3. In Vitro Studies

#### 3.3.1. Biological Materials and Assays

Human biphenotypic B myelomonocytic leukemia MV4-11, human pancreas cancer MiaPaca-2 cells, human melanoma Hs294T cells, human colon cancer HCT116 cells, and human normal epithelial cells from the mammary gland (MCF-10A) were obtained from American Type Culture Collection (ATCC, Rockville, MD, USA), while human lung carcinoma A549, human breast cancer MCF-7, and human prostate adenocarcinoma PC-3 were obtained from European Collection of Authenticated Cell Cultures (Culture Collections UK Health Security Agency, Porton Down, Salisbury, UK). All the cell lines were maintained at the Hirszfeld Institute of Immunology and Experimental Therapy, Polish Academy of Sciences, Wrocław, Poland.

MV4-11 and PC-3 cells were cultured in RPMI 1640 medium (HIIET PAS, Wrocław, Poland) with 1.0 mM sodium pyruvate (only MV4-11) and 10% fetal bovine serum (FBS) (all from Merck, Darmstadt, Germany). A549 cells were cultured in RPMI 1640+Opti-MEM (1:1) (HIIET PAS, Poland and Gibco, Paisley, UK) supplemented with 5% fetal bovine serum (Merck, Darmstadt, Germany). The MCF-7 cells were cultured in Eagle medium (HIIET PAS, Wrocław, Poland) supplemented with 10% FBS, 8 µg/mL of insulin, and 1% of MEM non-essential amino acid (all Merck, Darmstadt, Germany). Hs294T cells were cultured in RPMI 1640+Opti-MEM (1:1) (HIIET PAS, Poland, Wrocław and Gibco, Paisley, UK), supplemented with 10% fetal bovine serum and 4.5 g/L of glucose (all from Merck, Darmstadt, Germany). HCT116 cells were cultured in McCoy’s 5A medium (Gibco, Paisley, UK) supplemented with 10% FBS (Merck, Darmstadt, Germany). MiaPaca-2 cells were cultured in Dulbecco medium (Gibco, Paisley, UK) supplemented with 10% FBS and 2.5% HS (horse serum) (all from Merck, Darmstadt, Germany). MCF-10A cells were cultured in Ham’s F12 medium (Gibco, Paisley, UK) supplemented with 7.5% HS, 20 ng/mL of EGFh, 10 µg/mL of insulin, 0.5 µg/mL of hydrocortisone, and 0.05 mg/mL of cholera toxin (from Vibrio cholerae) (all from Merck, Darmstadt, Germany). All culture media were supplemented with 2 mM L-glutamine (Merck, Darmstadt, Germany), 100 units/mL of penicillin, (Polfa Tarchomin S.A., Tarchomin, Poland), and 100 µg/mL of streptomycin (Merck, Darmstadt, Germany). All cell lines were grown at 37 °C with 5% CO_2_ humidified atmosphere.

#### 3.3.2. Determination of Antiproliferative Activity

The tested compounds were dissolved in DMSO (dimethyl sulfoxide) to a concentration of 10 mg/mL and then were diluted in the culture medium to reach the final concentrations. The cells were plated in 384-well plates at a density of 1 × 10^3^ or 2 × 10^3^ (MCF-10A) cells per well (Greiner Bio One, Kremsmünster, Austria) and in 96-well plates (MV4-11 cell; Sarstedt, Nümbrecht, Germany) at a density of 5 × 10^3^ cells per well. After 24 h of incubation, the tested compounds were added at concentrations in the range of 100–0.03 µg/mL and, after the next 72 h of exposure to tested agents, the cytotoxic effect of all agents was examined using the MTT (MV4-11) or SRB assay, described previously [49]. The values of IC_50_ (inhibitory concentration 50%) were calculated separately for each experiment (repeated 3–5 times) using the Prolab-3 system which is based on Cheburator 0.4 software [49]. Doxorubicin was applied as a reference drug. The IC_50_ values in µg/mL were calculated to µM.

#### 3.3.3. MTT Assay

This assay was used against MV4-11 leukemia cells and was performed after 72 h of exposure of the tested agents to the cells. For each well, 20 µL of the MTT [3-(4,5-dimethylthiazol-2-yl)-2,5-diphenyl tetrazolium bromide] solution (5 mg/mL, Merck, Darmstadt, Germany) was added. After the incubation time (4 h at 37 °C), 80 µL of the lysing buffer was added to each well (mixture of 225 mL of DMF (dimethylformamide) Avantor, Poland; 67.5 g of SDS, Merck, Darmstadt, Germany; and 275 mL of distilled water). After the next 24 h, the absorbance of the samples was measured at a 570 nm wavelength on a Synergy H4 photometer (BioTek Instruments, Winooski, VT, USA). The absorbance of wells without the cells was the background [49].

#### 3.3.4. SRB Assay

The cytotoxicity assay was performed after the cultured cells were exposed to the tested agents for 72 h using the authomatic Washer dyspenser EL406, BioTek. At the first step, the washer dispenser collected 40 µL of cultured medium from each wells, and then the cells were fixed by gently layering 20 µL per well of cold 25% trichloroacetic acid to each well. The incubation time was 40 min and then the plates were washed with tap water. The cells were stained with 20 µL per well of 0.1% sulforhodamine B (SRB, Merck, Darmstadt, Germany) for 30 min. Unbound sulforhodamine B was removed by washing it with 1% acetic acid. The protein-bound dye was extracted in 30 min using 70 µL per well of 10 mM unbuffered Tris base (Merck, Darmstadt, Germany). The absorbance (at 540 nm) was measured on a Synergy H4 photometer (BioTek Instruments, Winooski, VT, USA). The absorbance of the wells without the cells was the background [49].

#### 3.3.5. Cell Cycle Analysis

The MV4-11 cells were seeded on 12-well plates (Greiner Bio One) and, after 24 h, the cells were exposed to the tested compounds at concentrations of about 1.5 × IC_50_ for 24 and 72 h. After incubation, the cells were collected and counted with a hemocytometer, and the 5×10^5^ cells were washed twice in cold PBS (phosphate-buffered saline) and fixed for 24 h in 70% ethanol at −20 °C. Then, the cells were washed twice in PBS and incubated with 0.5 mL of RNAse (8 μg/mL, Thermo Fisher, Waltham, MA, USA) at 37 °C for 1 h. The cells were stained for 30 min with propidium iodide (final concentration: 50 μg/mL, Merck, Darmstadt, Germany) at 4 °C and the cellular DNA content was analyzed by conducting flow cytometry using a BD LSRFortessa cytometer (BD Bioscience, San Jose, CA, USA). Compounds at each concentration were tested at least three times independently. The obtained results were analyzed using Flowing software 2.5.1 (Cell Imaging Core, Turku Centre for Biotechnology, University of Turku and Åbo Akademi University, Finland).

#### 3.3.6. Apoptosis Studies Using Annexin V and Propidium Iodide Staining

The human leukemia MV4-11 cells were seeded on 12-well plates (Greiner Bio One) and were exposed to the tested compounds at 2 concentrations of about 1.5×IC_50_ and 2×IC_50_ for 24 and 72 h. Camptothecin at a concentration of 0.05 uM was used as a positive control. After incubation, the cells were collected and counted with a hemocytometer, and 1×10^5^ cells were washed twice with PBS. APC-Annexin V (Becton Dickinson, Pharmingen, Franklin Lakes, NJ, USA) was dissolved to the concentration of 1 mg/mL in a binding buffer (10 mM HEPES/NaOH, pH 7.4; 150 mM NaCl; 5 mM KCl; 1 mM MgCl_2_; and 1.8 mM CaCl_2_) (HIIET, Wrocław, Poland), and the cells were suspended in 200 µL of this solution. After 15 min of the incubation in the dark at room temperature, 20 µL of propidium iodide (PI) solution (0.02 mg/mL) was added. Data acquisition was performed by conducting flow cytometry using a BD LSRFortessa cytometer (BD Bioscience, San Jose, CA, USA). The compounds were tested at least three times independently. Results were analyzed using Flowing software 2.5.1 (Turku, Finland). The data were displayed as a two-color dot plot with an APC-Annexin V vs. PI. Double-negative cells were live cells, PI+/Annexin V+ were late apoptotic cells, PI-/Annexin V+ were early apoptotic cells, and PI+/Annexin V- were necrotic cells.

#### 3.3.7. Caspase-3/7 Activity Determination

The MV4-11 cells were seeded on 48-well plates to the final volume of 1 mL. The cells were exposed to the tested compounds at concentrations of about 1.5 × IC_50_ and 2 × IC_50_, and camptothecin (0.05 µg/mL) was used as a positive control for 24 and 72 h. After incubation, the cells were collected and centrifuged (5 min, 4 °C, 250× *g*). Cells were suspended in 50 µL of ice-cold lysis buffer (50 mM HEPES; 10% (*w*/*v*) sucrose; 150 mM NaCl; 2 mM EDTA; 1% (*v*/*v*) Triton X-100, pH 7.3; and HIIET, Wrocław, Poland) and incubated for 30 min at 4 °C. After the incubation, 40 µL of each sample was transferred to a white 96-well plate (Corning, USA) containing 160 µL of the reaction buffer (20 mM HEPES; 10% sucrose; 100 mM NaCl; 1 mM EDTA; 10 mM DTT; and 0.02% Trition X-100, pH 7.3) (HIIET, Wrocław, Poland) with a 9 µM AC-DEVD-AMC (Cayman Chemical, Ann Arbor, MI, USA) fluorogenic substrate (λ_ex_ = 360 nm, λ_em_ = 460 nm). The correlation of the fluorescence increase with the caspase-3/7 level was continuously recorded at 37 °C for 120 min using Biotek Synergy H4 (Biokom, Warsaw, Poland). Compounds were tested in duplicates in single experiments, and each experiment was repeated at least three times independently. Results were normalized to the number of cells in each well and are reported as the mean relative caspase-3/7 activity compared to the untreated control sample ± SD.

#### 3.3.8. Statistical Analysis

Kruskal–Wallis tests (using GraphPad Prism 7) were performed to indicate significant differences in the comparison of two groups (control vs. study). A statistically significant difference was considered at *p* ≤ 0.05.

### 3.4. In Silico Study

Molecular parameters calculated using the SwissADME website [50] enabled the assessment of the drug likeness of selected betulin derivatives.

## 4. Conclusions

Betulin, a naturally occurring pentacyclic triterpene, can be used as a starting material in the synthesis of derivatives exhibiting various directions of biological activity, including anticancer activity. New bifunctional derivatives were obtained by introducing a carboxyacyl group at C-3 and an alkynyl group at C-28 into the betulin molecule. 3,28-Disubstituted betulin derivatives were evaluated for their anticancer activity against MV4-11, MCF-7, A549, PC-3, MiaPaca-2, HCT116, and Hs249T cells.

The presence of a 3-carboxyacyl substituent in both the betulin (compounds **4**–**6**) and 28-O-acetylbetulin (compounds **1**–**3**) systems increases the antiproliferative activity compared to the unsubstituted counterparts. This effect is most evident for dimethylglutaric derivatives **1** and **4**. The strong antiproliferative properties of the C-28 propynoyl-substituted derivatives (**4a**, **5a**, and **6a**), especially against the leukemia cell line MV4-11, indicate that these compounds may be candidates for in vivo studies.

The results of the study on the mechanism of action in MV4-11 cells performed for the five most active derivatives indicate that they can induce apoptosis.

## Figures and Tables

**Table 1:** Antiproliferative activity of the tested betulins and references.

Compound	IC_50_ [µM]							
	MV4-11	A549	MCF-7	PC-3	HCT116	MiaPaca-1	Hs249T	MCF-10A
**1**	2.90 ± 0.26	4.94 ± 0.48	7.02 ± 2.39	14.04 ± 7.02	6.38 ± 2.87	23.61 ± 3.83	11.64 ± 1.12	33.18 ± 1.91
**2**	4.40 ± 1.63	4.57 ± 1.79	16.64 ± 7.67	20.07 ± 2.12	10.93 ± 2.94	28.23 ± 4.40	27.57 ± 0.65	33.94 ± 4.24
**3**	3.93 ± 1.54	5.13 ± 0.85	17.27 ± 6.33	22.40 ± 2.56	13.16 ± 1.02	34.54 ± 5.47	21.88 ± 4.27	33.51 ± 1.88
**4**	4.77 ± 0.44	3.76 ± 2.05	12.14 ± 3.08	19.15 ± 3.76	11.11 ± 2.56	22.57 ± 6.50	24.45 ± 8.89	31.46 ± 1.71
**4a**	0.38 ± 0.14	7.54 ± 1.25	16.48 ± 1.88	0.86 ± 0.25	7.85 ± 2.35	7.54 ± 0.47	0.49 ± 0.23	33.13 ± 5.18
**4b**	3.19 ± 1.29	4.06 ± 1.05	11.73 ± 2.41	11.58 ± 4.51	7.82 ± 2.86	24.51 ± 2.56	10.68 ± 1.50	32.63 ± 2.41
**4c**	3.23 ± 1.38	7.37 ± 2.30	16.59 ± 2.15	10.14 ± 2.46	8.45 ± 2.15	22.74 ± 5.53	9.68 ± 1.54	32.57 ± 2.15
**4d**	5.15 ± 0.47	6.24 ± 1.25	20.59 ± 5.77	21.68 ± 4.52	16.85 ± 5.61	34.17 ± 2.96	18.72 ± 9.67	60.38 ± 15.91
**4e**	3.16 ± 0.88	59.82 ± 22.16	25.70 ± 4.43	15.21 ± 5.91	23.19 ± 5.91	67.06 ± 14.48	19.79 ± 5.61	67.95 ± 34.86
**4f**	46.56 ± 11.78	Neg	130.71 ± 8.83	95.79 ± 27.07	Neg	Neg	Neg	Neg
**5**	7.81 ± 2.80	9.11 ± 0.87	29.25 ± 3.85	26.97 ± 3.85	14.71 ± 4.55	30.65 ± 2.63	30.83 ± 1.40	26.80 ± 2.10
**5a**	0.600 ± 0.175	10.72 ± 3.0	19.43 ± 3.1	0.85 ± 0.17	3.64 ± 0.6	-	-	12.7 ± 0.5
**5b**	4.76 ± 0.92	4.15 ± 0.92	12.29 ± 2.92	21.05 ± 1.54	9.06 ± 2.92	28.42 ± 3.23	16.13 ± 4.76	33.80 ± 3.38
**5c**	10.99 ± 1.10	6.59 ± 1.88	24.96 ± 4.87	22.92 ± 4.71	9.26 ± 0.47	32.03 ± 6.12	23.24 ± 3.77	32.03 ± 4.87
**5d**	2.50 ± 0.48	4.62 ± 1.59	21.05 ± 9.89	16.59 ± 6.54	12.28 ± 4.15	29.19 ± 1.59	59.66 ± 38.60	34.13 ± 4.94
**6**	6.45 ± 2.03	8.66 ± 1.10	18.97 ± 6.45	26.16 ± 5.71	20.26 ± 5.53	38.13 ± 3.87	32.06 ± 1.66	31.87 ± 4.24
**6a**	2.57 ± 1.01	12.94 ± 0.67	15.97 ± 1.01	0.41 ± 0.08	20.68 ± 9.08	18.49 ± 9.75	3.53 ± 0.67	49.42 ± 17.82
**6b**	3.53 ± 0.64	3.21 ± 0.64	11.40 ± 4.17	13.16 ± 2.57	9.95 ± 0.64	27.45 ± 2.57	17.50 ± 6.26	30.66 ± 3.69
**A**	41.05 ± 8.46	27.84 ± 10.52	72.40 ± 6.60	80.65 ± 13.41	40.64 ± 13.61	126.86 ± 16.30	127.69 ± 14.23	123.98 ± 33.00
**B**	32.75 ± 12.87	14.00 ± 1.13	43.59 ± 15.81	51.50 ± 9.94	37.72 ± 7.45	144.33 ± 22.13	58.50 ± 15.58	98.48 ± 0.90
**C ^a^**	0.021 ± 0.008	0.040 ± 0.012	0.150 ± 0.015	1.27 ± 0.2	0.118 ± 0.02	-	-	0.114 ± 0.013

28-O-acetylbetulin; **B**—betulin; **C**—doxorubicin;^“a”^ —literature data [36]; “-“—not tested.

**Table 2:** The SI index = IC_50_ for the normal cell line (MCF-10A) and IC_50_ for the respective cancerous cell line. A beneficial SI > 1.0 indicates a drug with efficacy against tumor cells greater than toxicity against normal cells.

Compound	SI						
	MV4-11	A549	MCF-7	PC-3	HCT116	MiaPaca-1	Hs249T
**1**	11.44	6.71	4.73	2.36	5.20	1.41	2.85
**2**	7.70	7.43	2.04	1.69	3.10	1.20	1.23
**3**	8.82	6.53	1.94	1.50	2.55	0.97	1.53
**4**	6.60	8.36	2.60	1.64	2.83	1.39	1.29
**4a**	98.92	4.40	2.01	38.57	4.22	4.40	67.0
**4b**	10.20	8.04	2.78	2.82	4.17	1.33	3.06
**4c**	10.10	4.42	1.96	3.21	3.85	1.43	3.37
**4d**	11.73	9.68	2.93	2.78	3.58	1.77	3.23
**4e**	21.50	1.14	2.60	4.47	2.93	1.01	3.40
**5**	3.43	2.94	0.92	1.01	1.82	0.87	0.87
**5a**	21.17	1.18	0.65	14.94	3.49	-	-
**5b**	7.10	8.15	2.75	1.61	3.73	1.19	2.10
**5c**	2.91	4.86	1.28	1.40	3.46	1.00	1.38
**5d**	13.63	7.38	1.62	2.06	2.78	1.17	0.57
**6**	4.94	3.68	1.68	1.22	1.57	0.84	0.99
**6a**	19.22	3.82	3.09	121.0	2.39	2.67	14.0
**6b**	8.68	9.55	2.69	2.33	3.08	1.12	1.75
**28-O-acetylbetulin**	3.02	4.45	1.71	1.54	3.05	0.98	0.97
**betulin**	3.01	7.03	2.26	1.91	2.61	0.68	1.68

## Data Availability

The original contributions presented in the study are included in the article/supplementary material; further inquiries can be directed to the corresponding author/s.

## Supplementary Materials

The following supporting information can be downloaded at the following: www.mdpi.com/article/10.3390/molecules30030611/s1. Figure S1. Reaction scheme of 28-*O*-acetylbetuline with 2,2-dimethylsuccinic anhydride; Figure S2. The ^1^H-^13^C HSQC spectra of 28-acetyl-3-(3’,3’-dimethylsuccinyl)betulin 2; Figure S3. Structure of compound 2 showing proton-carbon interactions (through two and three bonds) in substituents at C-3 and C-28 atoms; Figure S4. The ^1^H-^13^C HMBC spectra of 28-acetyl-3-(3’,3’-dimethylsuccinyl)betulin 2; Figure S5. ^1^H NMR, compound 1; Figure S6. ^13^C NMR, compound 1; Figure S7. HRMS, compound 1; Figure S8. ^1^H NMR, compound 2; Figure S9. ^13^C NMR, compound 2; Figure S10. HRMS, compound 2; Figure S11. ^1^H NMR, compound 3; Figure S12. ^13^C NMR compound 3; Figure S13. HRMS, compound 3; Figure S14. ^1^H NMR, compound 4a; Figure S15. ^13^C NMR, compound 4a; Figure S16. HRMS, compound 4a; Figure S17. ^1^H NMR, compound 4b; Figure S18. ^13^C NMR, compound 4b; Figure S19. HRMS, compound 4b; Figure S20. ^1^H NMR, compound 4c; Figure S21. ^13^C NMR, compound 4c; Figure S22. HRMS, compound 4c; Figure S23. ^1^H NMR, compound 4d; Figure S24. ^13^C NMR, compound 4d; Figure S25. HRMS, compound 4d; Figure S26. ^1^H NMR, compound 4e; Figure S27. ^13^C NMR, compound 4e; Figure S28. HRMS, compound 4e; Figure S29. ^1^H NMR, compound 4f; Figure S30. ^13^C NMR, compound 4f; Figure S31. HRMS, compound 4f; Figure S32. ^1^H NMR, compound 5a; Figure S33. ^13^C NMR, compound 5a; Figure S34. HRMS, compound 5a; Figure S35. ^1^H NMR, compound 5b; Figure S36. ^13^C NMR, compound 5b; Figure S37. HRMS, compound 5b; Figure S38. ^1^H NMR, compound 5c; Figure S39. ^13^C NMR, compound 5c; Figure S40. HRMS, compound 5c; Figure S41. ^1^H NMR, compound 5d; Figure S42. ^13^C NMR, compound 5d; Figure S43. HRMS, compound 5d; Figure S44. ^1^H NMR, compound 6a; Figure S45. ^13^C NMR, compound 6a; Figure S46. HRMS, compound 6a; Figure S47. ^1^H NMR, compound 6b; Figure S48. ^13^C NMR, compound 6b; Figure S49. HRMS, compound 6b; Table S1. The selected proton–carbon correlations of 28-acetyl-3-(3’,3’-dimethylsuccinyl)betulin 2.
