# Supplementary material for: New Difunctional Derivatives of Betulin: Preparation, Characterization and Antiproliferative Potential
Source: Molecules. 2025 Jan 30;30(3):611. doi: 10.3390/molecules30030611 (PMC11821258; doi:10.3390/molecules30030611)
Supplement: Supplementary file 1 [file molecules-30-00611-s001.zip › molecules-3374165-supplementary.pdf]

# Supplementary Materials

# New Difunctional Derivatives of Betulin: Preparation, Characterization and Antiproliferative Potential

**Elwira Chrobak [1,*], Marta Świtalska [2], Joanna Wietrzyk [2] and Ewa Bębenek [1,*]**

[1] Department of Organic Chemistry, Faculty of Pharmaceutical Sciences in Sosnowiec, Medical University of Silesia in Katowice, 4 Jagiellońska Str., 41-200 Sosnowiec, Poland; echrobak@sum.edu.pl (EC), ebebenek@sum.edu.pl (EB)
[2] Hirszfeld Institute of Immunology and Experimental Therapy, Polish Academy of Sciences, 12 Rudolfa Weigla Str., 53-114 Wrocław, marta.switalska@hirszfeld.pl (MŚ), joanna.wietrzyk@hirszfeld.pl (JW)
* Correspondence: Correspondence: echrobak@sum.edu.pl (EC), ebebenek@sum.edu.pl (EB)

**Content:**

Determination of the structure of compound **2**

**Figure S1**. Reaction scheme of 28-*O*-acetylbetuline with 2,2-dimethylsuccinic anhydride.
**Figure S2**. The $^{1}$H-$^{13}$C HSQC spectra of 28-acetyl-3-(3′,3′-dimethylsuccinyl)betulin **2**.
**Figure S3**. Structure of compound 2 showing proton-carbon interactions (through two and three bonds) in substituents at C-3 and C-28 atoms.
**Figure S4**. The $^{1}$H-$^{13}$C HMBC spectra of 28-acetyl-3-(3′,3′-dimethylsuccinyl)betulin **2**.
**Table S1**. The selected proton–carbon correlations of 28-acetyl-3-(3′,3′-dimethylsuccinyl)betulin **2**.

Spectroscopic data for the compound **1-3**, **4a-4f**, **5a-5d** and **6a-6b**

**Figure S5**. $^{1}$H NMR, compound **1**
**Figure S6.** $^{13}$C NMR, compound **1**
**Figure S7.** HRMS, compound **1**
**Figure S8**. $^{1}$H NMR, compound **2**
**Figure S9.** $^{13}$C NMR, compound **2**
**Figure S10.** HRMS, compound **2**
**Figure S11**. $^{1}$H NMR, compound **3**
**Figure S12.** $^{13}$C NMR, compound **3**
**Figure S13.** HRMS, compound **3**
**Figure S14**. $^{1}$H NMR, compound **4a**
**Figure S15.** $^{13}$C NMR, compound **4a**
**Figure S16.** HRMS, compound **4a**
**Figure S17**. $^{1}$H NMR, compound **4b**
**Figure S18.** $^{13}$C NMR, compound **4b**
**Figure S19.** HRMS, compound **4b**
**Figure S20**. $^{1}$H NMR, compound **4c**
**Figure S21.** $^{13}$C NMR, compound **4c**

**Figure S22.** HRMS, compound **4c**
**Figure S23**. $^{1}$H NMR, compound **4d**
**Figure S24.** $^{13}$C NMR, compound **4d**
**Figure S25.** HRMS, compound **4d**
**Figure S26**. $^{1}$H NMR, compound **4e**
**Figure S27.** $^{13}$C NMR, compound **4e**
**Figure S28.** HRMS, compound **4e**
**Figure S29**. $^{1}$H NMR, compound **4f**
**Figure S30.** $^{13}$C NMR, compound **4f**
**Figure S31.** HRMS, compound **4f**
**Figure S32**. $^{1}$H NMR, compound **5a**
**Figure S33.** $^{13}$C NMR, compound **5a**
**Figure S34.** HRMS, compound **5a**
**Figure S35**. $^{1}$H NMR, compound **5b**
**Figure S36.** $^{13}$C NMR, compound **5b**
**Figure S37.** HRMS, compound **5b**
**Figure S38**. $^{1}$H NMR, compound **5c**
**Figure S39.** $^{13}$C NMR, compound **5c**
**Figure S40.** HRMS, compound **5c**
**Figure S41**. $^{1}$H NMR, compound **5d**
**Figure S42.** $^{13}$C NMR, compound **5d**
**Figure S43.** HRMS, compound **5d**
**Figure S44**. $^{1}$H NMR, compound **6a**
**Figure S45.** $^{13}$C NMR, compound **6a**
**Figure S46.** HRMS, compound **6a**
**Figure S47.** $^{1}$H NMR, compound **6b**
**Figure S48.** $^{13}$C NMR, compound **6b**
**Figure S49.** HRMS, compound **6b**

Determination of the structure of compound **2**

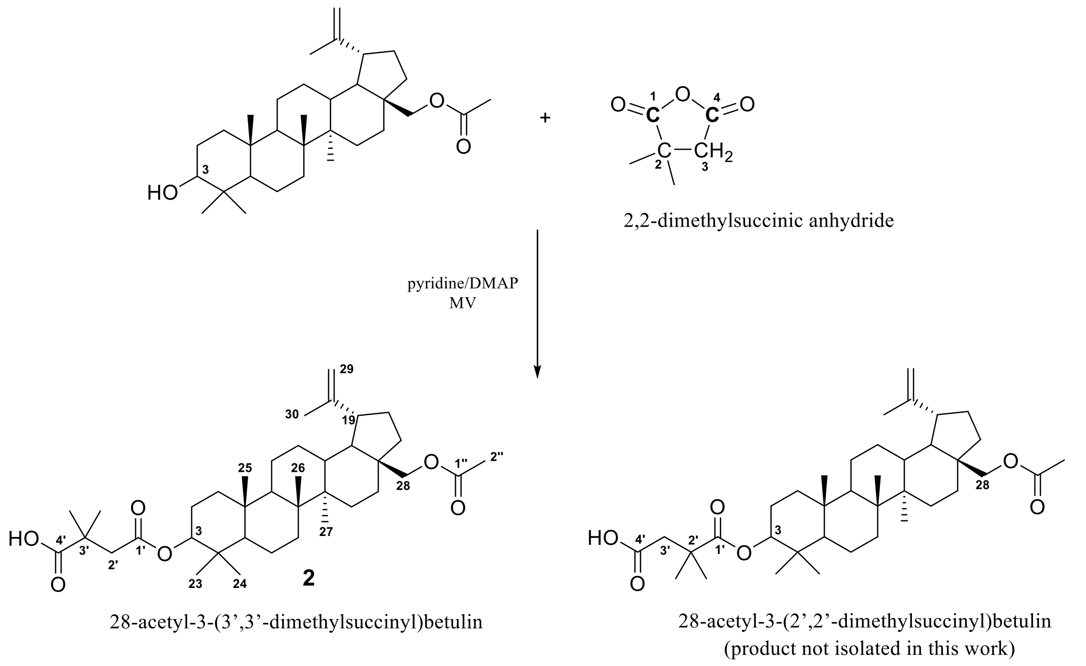


**Figure S1**. Reaction scheme of 28-*O*-acetylbetuline with 2,2-dimethylsuccinic anhydride.

The structure of the substituents present at C-3 and C-28 positions of compound 2 was determined based on the results of HSQC (*Heteronuclear Single Quantum Correlation*) and HMBC (*Heteronuclear Multiple Bond Correlation*) experiments. Two-dimensional HSQC and HMBC spectra of compound **2** were recorded on a Bruker Avance spectrometer, using HSQCGPPH and HMBCGP experiments. The HSQC spectrum was used to assign the chemical shifts of the carbon atoms bonded to the corresponding protons of the substituents (Figure S2, Table S1).

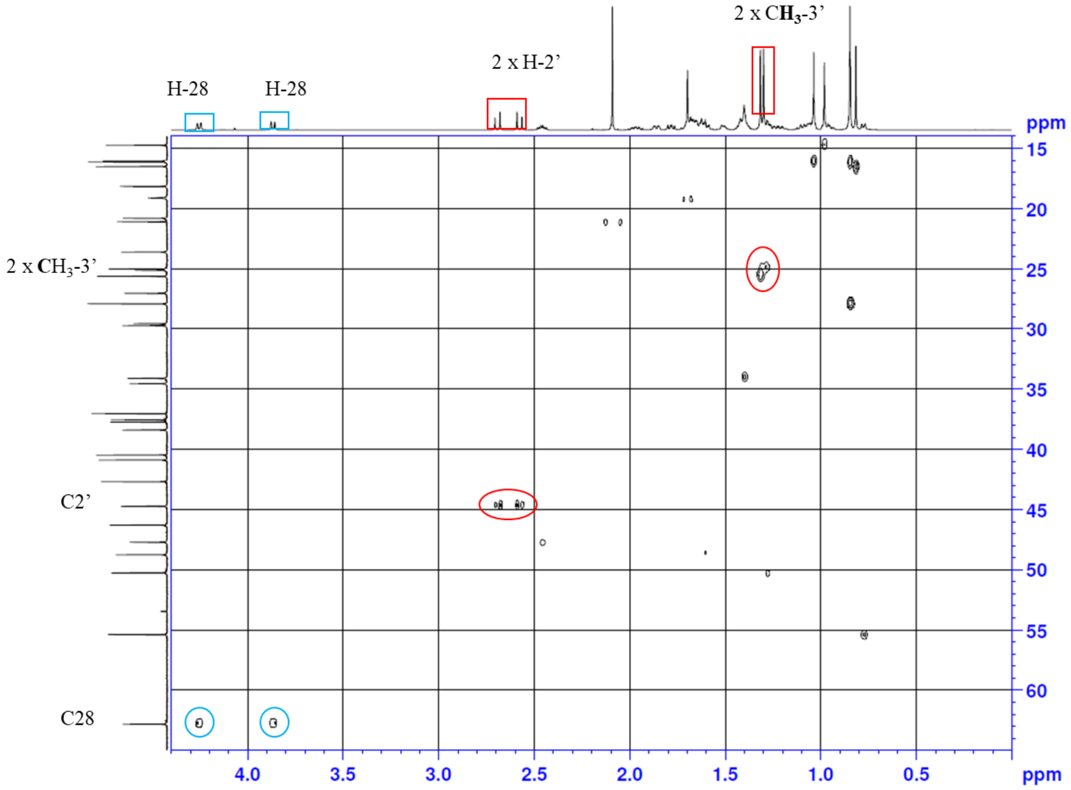


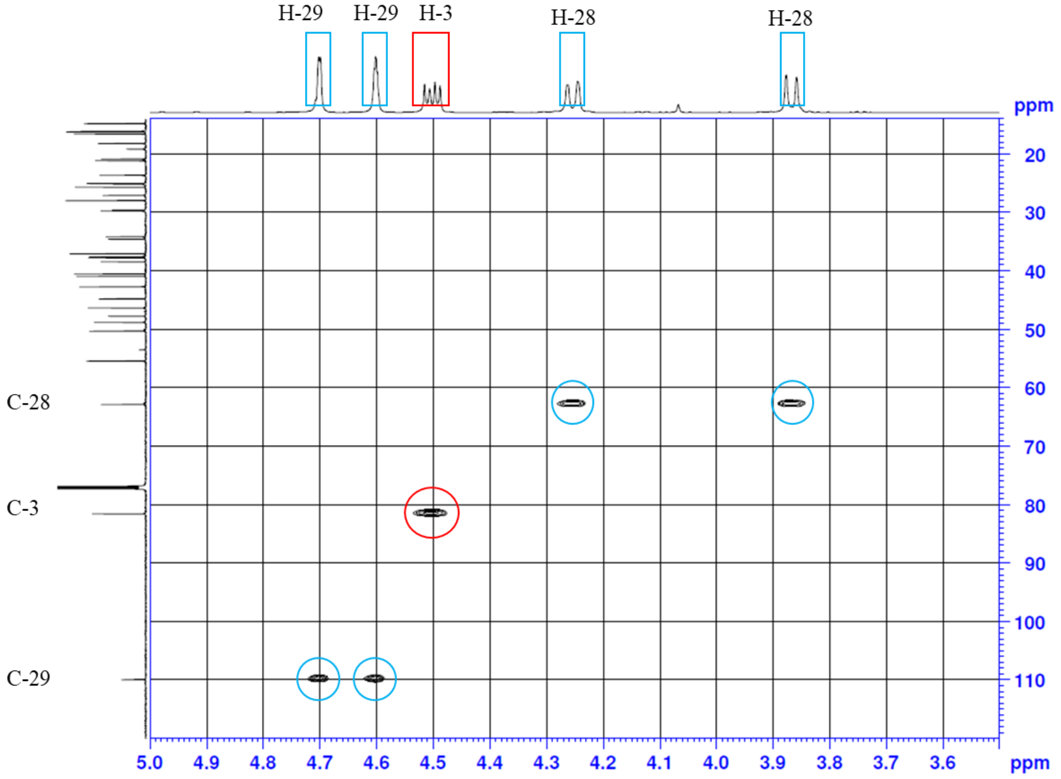


**Figure S2**. The $^{1}H$-$^{13}C$ HSQC spectra of 28-acetyl-3-(3′,3′-dimethylsuccinyl)betulin **2**.

The interactions of these protons via two or three bonds visible in the HMBC spectrum allowed the isolation protons of the substituent at C-28 and the assignment of the appropriate structure of the dimethylsuccinyl substituent at C-3 (Figure S3 and S4, Table 1).

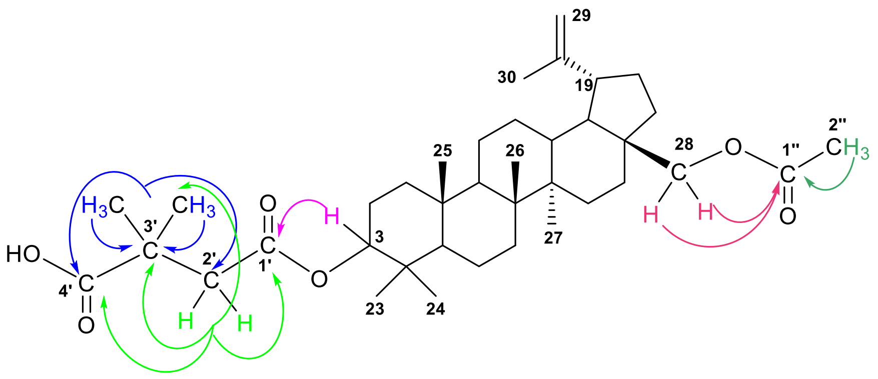


**Figure S3**. Structure of compound 2 showing proton-carbon interactions (through two and three bonds) in substituents at C-3 and C-28 atoms.

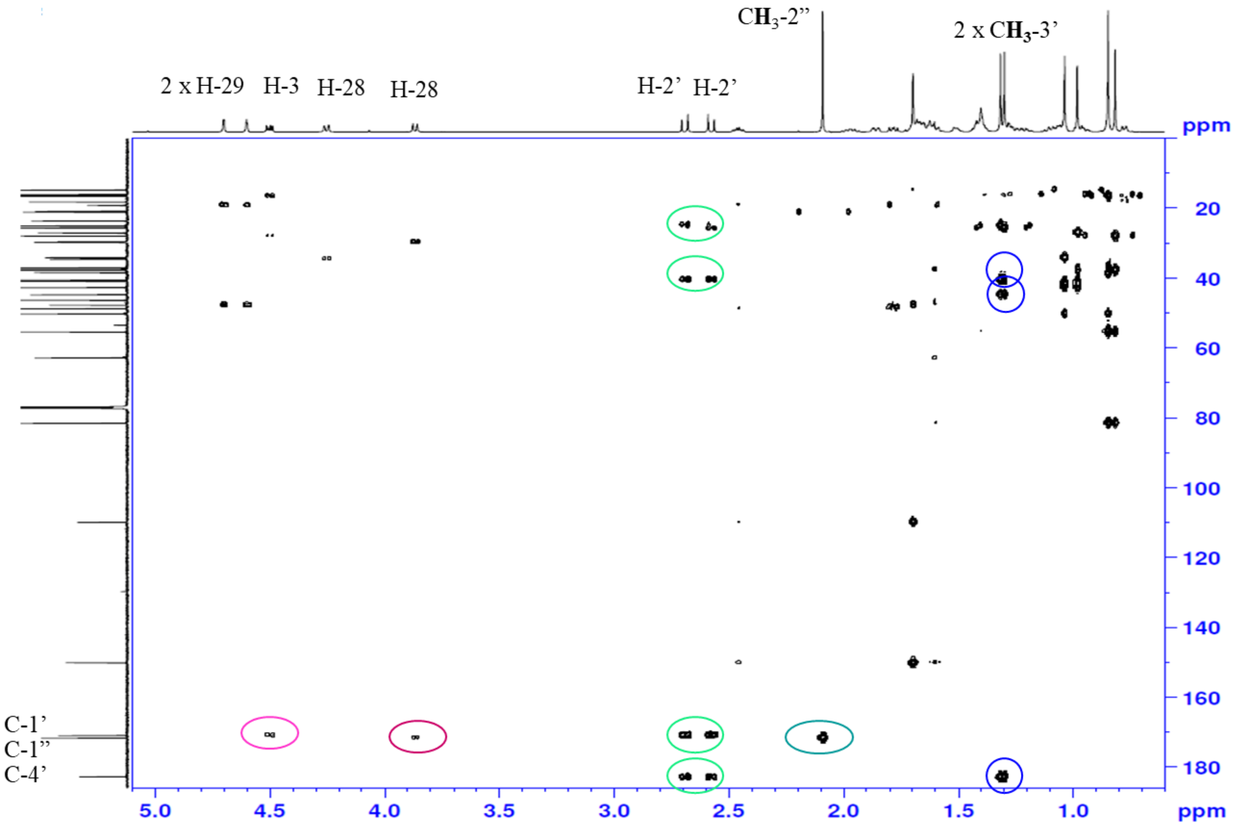

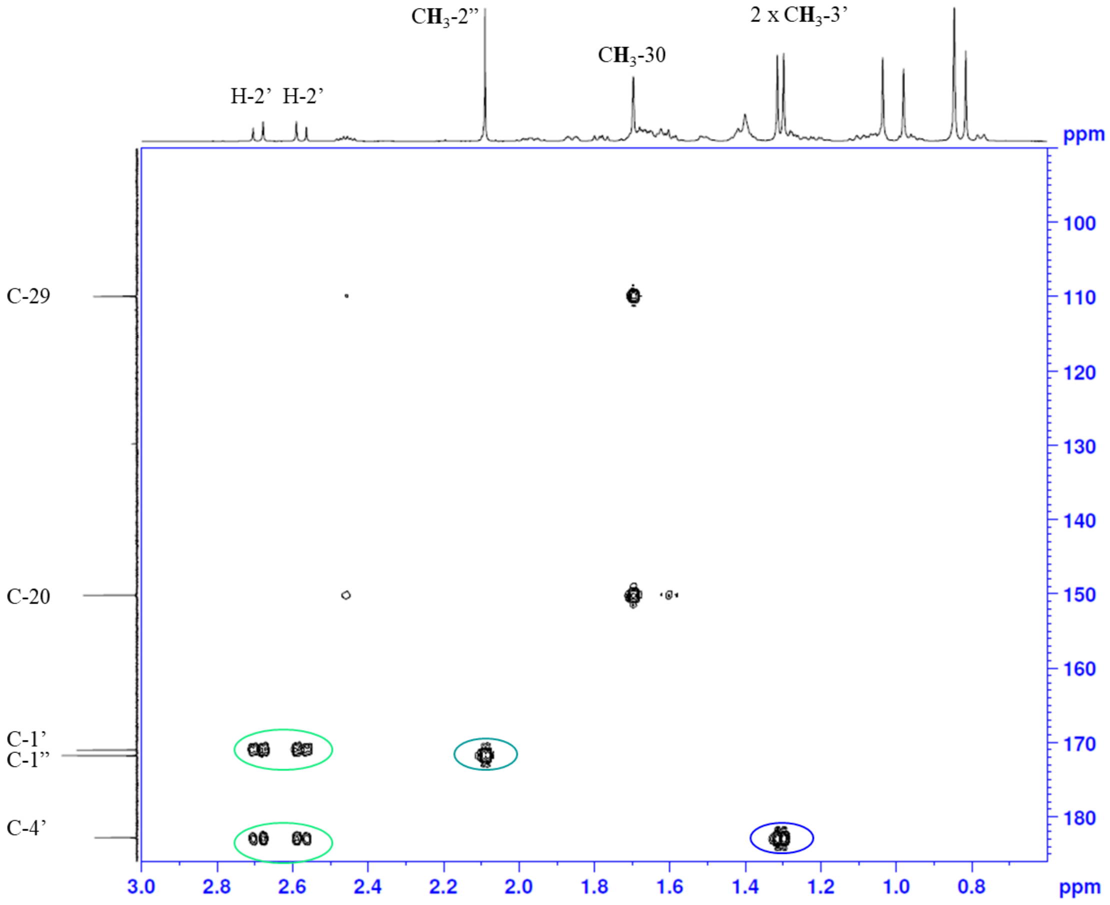


**Figure S4**. The $^{1}$H-$^{13}$C HMBC spectra of 28-acetyl-3-(3′,3′-dimethylsuccinyl)betulin **2**.

**Table S1**. The selected proton–carbon correlations of 28-acetyl-3-(3′,3′-dimethylsuccinyl)betulin **2**.

| $^{1}$H NMR δ [ppm] | HSQC | HMBC |
|---|---|---|
| 4.50 **H-3** | 4.50 - 81.6 (**C**-3) | 4.50 – 171.0 (**C**-1′) |
| 2.59, 2.70<br>2 x **H-2′** | 2.59 – 44.7 (**C**-2′)<br>2.70 – 44.7 (**C**-2′) | 2.59, 2.70 - 171.0 (**C**-1′)<br>181.8 (**C**-4′)<br>37.7 (**C**-3′)<br>25.6 (**C**$H_3$-3′) |
| 1.30, 1.31<br>2 x C$H_3$-3′ | 1.30 - 25.0 (**C**$H_3$-3′)<br>1.31 - 25.6 (**C**$H_3$-3′) | 1.30, 1.31 - 181.8 (**C**-4′)<br>44.7 (**C**-2′)<br>37.7 (**C**-3′) |
| 3.87, 4.26<br>2 x **H-28** | 3.87 – 62.8 (**C**-28)<br>4.26 – 62.8 (**C**-28) | 3.87, 4.26 –171.7 (**C**-1″) |
| 2.10 C$H_3$-2″ | 2.10 – 20.8 (**C**$H_3$-2″) | 2.10 – 171.7 (**C**-1″) |

Spectroscopic data for the compound **1**-**3**, **4a**-**4f**, **5a**-**5d** and **6a**-**6b**

### Compound 1: 28-acetyl-3-(3′,3′-dimethylglutaryl)betulin

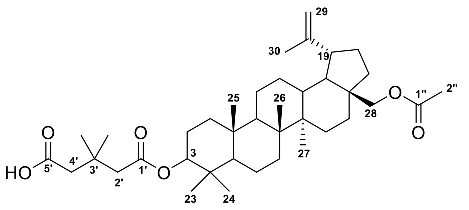

Yield 73%; mp 112-114°C; $R_f$ 0.20 (dichloromethane/ethanol, 40:1, *v/v*). $^1$H NMR ($CDCl_3$, 600 MHz) δ (ppm): 0.79-0.81 (m, 1H, H-5), 0.86 (s, 3H, $CH_3$-24), 0.87 (s, 3H, $CH_3$-23), 0.88 (s, 3H, $CH_3$-25), 0.99 (s, 3H, $CH_3$-27), 1.04 (s, 3H, $CH_3$-26), 1.16 (m, 6H, 2 x $CH_3$, dimethylglutaric), 1.71 (s, 3H, $CH_3$-30), 2.09-0.86 (m, 24H, CH, $CH_2$), 2.10 (s, 3H, $CH_3$-2″) 2.41-2.52 (m, 4H, 2 x $CH_2$, dimethylglutaric H-2′, H-4′; H-19), 3.86 (d, 1H, $^2J$ = 11.4 Hz, H-28), 4.27 (d, 1H, $^2J$ = 11.4 Hz, H-28), 4.51-4.54 (m, 1H, H-3), 4.61 (s, 1H, H-29), 4.71 (s, 1H, H-29). $^{13}$C NMR ($CDCl_3$, 150 MHz) δ (ppm): 14.7, 16.0, 16.1, 16.6, 18.2, 20.8, 21.1, 23.8, 25.1, 27.0, 28.0, 28.2, 28.3, 29.7, 33.0, 34.1, 37.0, 37.4, 37.5, 37.7, 38.3, 40.9, 40.9, 42.7, 42.7, 45.3 45.5, 46.3, 48.8, 50.2, 50.3, 55.4, 62.8, 82.0, 110.6, 150.3, 171.7, 171.7, 173.5. HRMS (APCI) m/z (neg): 625.4479; $C_{39}H_{61}O_6$ [M-H] (Calculated 625.4468).

### Compound 2: 28-acetyl-3-(3′,3′-dimethylsuccinyl)betulin

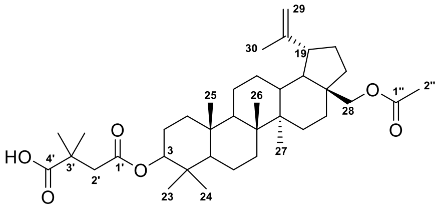

Yield 86%; mp 120-122°C; $R_f$ 0.15 (dichloromethane/ethanol, 15:1, *v/v*). $^1$H NMR ($CDCl_3$, 600 MHz) δ (ppm): 0.77-0.79 (m, 1H, H-5), 0.82 (s, 3H, $CH_3$-24), 0.85 (s, 6H, $CH_3$-23, 25), 0.98 (s, 3H, $CH_3$-27), 1.05 (s, 3H, $CH_3$-26), 1.30 (s, 3H, dimethylsuccinic $CH_3$-3′), 1.31 (s, 3H, dimethylsuccinic $CH_3$-3′), 1.70 (s, 3H, $CH_3$-30), 2.00-0.80 (m, 23 H, CH, $CH_2$), 2.10 (s, 3H, $CH_3$-2′′), 2.44-2.49 (m, 1H, H-19), 2.59 (d, 1H, dimethylsuccinic H-2′, $^2J$ =15.6 Hz), 2.70 (d, 1H, dimethylsuccinic H-2′ $^2J$=15.6 Hz), 3.87 (d, 1H, H-28 $^2J$=11.4 Hz), 4.26 (d, 1H, H- 28 $^2J$=11.4 Hz), 4.49-4.51 (m, 1H, H-3), 4.60 (s, 1H, H-29), 4.70 (s, 1H, H-29). $^{13}$C NMR ($CDCl_3$, 150 MHz) δ (ppm): 14.8, 16.0, 16.1, 16.5, 18.2, 20.8, 21.1, 23.6, 25.1, 25.0, 25.6, 27.0, 27.9, 29.5, 29.7, 34.1, 34.5, 37.0, 37.5, 37.7, 38.4, 40.5, 40.9, 42.7, 44.7, 46.3, 47.7, 48.7, 50.2, 53.4, 55.4, 62.8, 81.6, 109.9, 150.1, 171.0, 171.7, 181.8. HRMS (APCI) m/z (neg): 611.4306; $C_{38}H_{59}O_6$ [M-H] (Calculated 611.4311).

### Compound 3: 28-acetyl-3-succinylbetulin

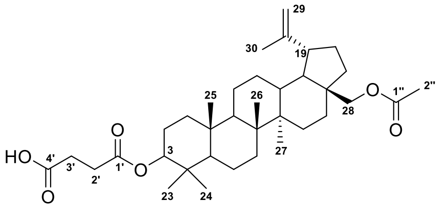

Yield 68%; mp 123-125°C; $R_f$ 0.43 (chloroform/ethanol, 15:1, v/v). $^1$H NMR ($CDCl_3$, 600 MHz) δ (ppm): 0.79-0.80 (m, 1H, H-5), 0.85 (s, 3H, $CH_3$-24), 0.86 (s, 6H, $CH_3$-23, 25), 0.99 (s, 3H, $CH_3$-27), 1.05 (s, 3H, $CH_3$-26), 1.70 (s, 3H, $CH_3$-30), 2.00-0.90 (m, 26 H, CH, $CH_2$), 2.09 (s, 3H, $CH_3$-2″), 2.46-2.47 (m, 1H, H-19), 2.65-2.67 (m, 2H, succinic $CH_2$), 2.69-2.70 (m, 2H, succinic $CH_2$), 3.87 (d, 1H, H-28 $^2J$=10.8 Hz), 4.27 (d, 1H, H-

28 $^{2}J$=10.8 Hz), 4.50-4.53 (m, 1H, H-3), 4.61 (s, 1H, H-29), 4.70 (s, 1H, H-29).$^{13}$C NMR ($CDCl_3$, 150 MHz) δ (ppm): 14.7, 16.0, 16.1, 16.5, 18.1, 19.1, 20.8, 21.1, 23.6, 25.1, 27.0, 27.9, 28.9, 29.3, 29.6, 29.7, 34.1, 34.5, 37.0, 37.5, 37.8, 38.3, 40.9, 42.7, 46.3, 47.7, 48.8, 50.3, 55.4, 62.8, 81.6, 109.9, 150.2, 171.7, 171.9, 176.9. HRMS (APCI) m/z (neg): 583.3977; $C_{36}H_{55}O_6$ [M-H] (Calculated 583.3998).

**Compound 4a: 28-propynoyl-3-(3′,3′-dimethylglutaryl)betulin**

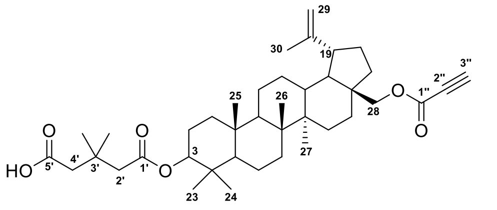


Yield 72%; mp 114-116°C; $R_f$ 0.58 (chloroform/ethanol, 15:1, v/v). $^{1}$H NMR (600 MHz, $CDCl_3$) δ (ppm): 0.79-0.80 (m, 1H, H-5), 0.85 (s, 3H, $CH_3$-24), 0.86 (s, 3H, $CH_3$-23), 0.87 (s, 3H, $CH_3$-25), 0.99 (s, 3H, $CH_3$-27), 1.04 (s, 3H, $CH_3$-26), 1.18 (m, 6H, 2 x $CH_3$, dimethylglutaric), 1.70 (s, 3H, $CH_3$-30), 2.00-0.85 (m, 24H, CH, $CH_2$), 2.44-2.51 (m, 4H, 2 x $CH_2$, dimethylglutaric H-2′, H-4′; H-19), 2.92 (s, 1H, C≡CH), 4.00 (d, 1H, $^{2}J$ = 10.8 Hz, H-28), 4.40 (d, 1H, $^{2}J$ = 10.8 Hz, H-28), 4.49-4.52 (m, 1H, H-3), 4.61 (s, 1H, H-29), 4.70 (s, 1H, H-29). $^{13}$C NMR (150 MHz, $CDCl_3$) δ (ppm): 14.8, 16.0, 16.1, 16.6, 18.2, 19.1, 20.8, 23.8, 25.1, 27.0, 28.0, 28.0, 28.0, 29.4, 29.6, 32.7, 34.1, 34.4, 37.0, 37.6, 37.7, 38.3, 40.9, 42.7, 45.2, 45.6, 46.4, 47.7, 48.8, 50.2, 55.4, 64.9, 74.8, 81.4, 110.1, 149.9, 153.3, 172.5, 176.5. HRMS (APCI) m/z (neg): 635.4391; $C_{40}H_{59}O_6$ [M-H] (Calculated 635.4311).

**Compound 4b: 28-pentynoyl-3-(3′,3′-dimethylglutaryl)betulin**

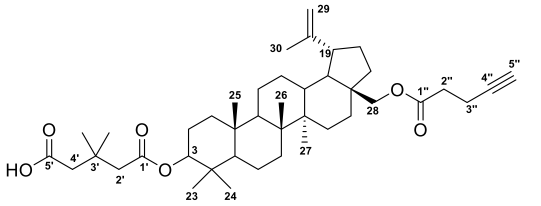


Yield 70%; mp 117-119°C; $R_f$ 0.51 (chloroform/ethanol, 15:1, v/v). $^{1}$H NMR (600 MHz, $CDCl_3$) δ (ppm): 0.79-0.81 (m, 1H, H-5), 0.86 (s, 3H, $CH_3$-24), 0.87 (s, 3H, $CH_3$-23), 0.88 (s, 3H, $CH_3$-25), 0.99 (s, 3H, $CH_3$-27), 1.05 (s, 3H, $CH_3$-26), 1.16 (m, 6H, 2 x $CH_3$, dimethylglutaric), 1.70 (s, 3H, $CH_3$-30), 2.00 (m, 1H, C≡CH, CH-5″), 2.00-0.86 (m, 24H, CH, $CH_2$), 2.44-2.48 (m, 4H, 2 x $CH_2$, dimethylglutaric H-2′, H-4′; H-19), 2.54-2.60 (m, 4H, 2 x $CH_2$, H-2″, H-3″), 3.89 (d, 1H, $^{2}J$ = 10.8 Hz, H-28), 4.32 (d, 1H, $^{2}J$ = 10.8 Hz, H-28), 4.51-4.54 (m, 1H, H-3), 4.61 (s, 1H, H-29), 4.71 (s, 1H, H-29). $^{13}$C NMR (150 MHz, $CDCl_3$) δ (ppm): 14.5, 14.8, 16.1, 16.6, 18.2, 19.1, 20.8, 23.8, 24.3, 25.1, 27.0, 28.0, 28.2, 28.3, 29.5, 29.7, 33.0, 33.6, 34.1, 34.6, 37.1, 37.6, 37.7, 38.3, 40.9, 42.7, 45.3, 46.4, 47.7, 48.8, 50.2, 55.4, 63.1, 69.1, 81.2, 82.5, 110.0, 150.1, 172.2, 173.2, 174.1. HRMS (APCI) m/z (neg): 663.4619; $C_{42}H_{63}O_6$ [M-H] (Calculated 663.4624).

**Compound 4c: 28-(2-butynoyl)- 3-(3′,3′-dimethylglutaryl)betulin**

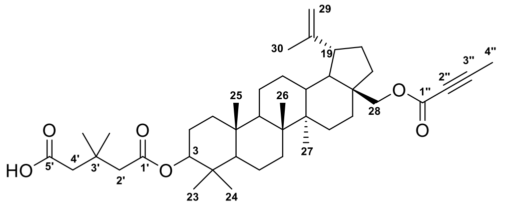

Yield 72%; mp 131-133°C; $R_f$ 0.56 (chloroform/ethanol, 15:1, v/v). $^1$H NMR (600 MHz, $CDCl_3$) δ (ppm): 0.79-0.80 (m, 1H, H-5), 0.85 (s, 3H, $CH_3$-24), 0.86 (s, 3H, $CH_3$-23), 0.87 (s, 3H, $CH_3$-25), 0.99 (s, 3H, $CH_3$-27), 1.04 (s, 3H, $CH_3$-26), 1.16 (m, 6H, 2 x $CH_3$, dimethylglutaric), 1.70 (s, 3H, $CH_3$-30), 1.98-0.85 (m, 24H, CH, $CH_2$), 2.01 (s, 3H, $CH_3$-4″), 2.44-2.48 (m, 4H, 2 x $CH_2$, dimethylglutaric H-2′, H-4′; H-19), 3.96 (d, 1H, $^2J$ = 10.8 Hz, H-28), 4.35 (d, 1H, $^2J$ = 10.8 Hz, H-28), 4.50-4.52 (m, 1H, H-3), 4.61 (s, 1H, H-29), 4.70 (s, 1H, H-29). $^{13}$C NMR (150 MHz, $CDCl_3$) δ (ppm): 3.9, 14.7, 16.0, 16.1, 16.6, 18.2, 19.1, 20.8, 23.8, 25.1, 27.0, 28.0, 28.0, 28.1, 29.5, 32.8, 34.1, 34.5, 37.0, 37.6, 37.7, 38.3, 40.9, 42.7, 45.2, 45.5, 46.4, 47.7, 48.8, 50.2, 53.5, 55.4, 64.3, 72.5, 81.6, 85.6, 110.0, 150.0, 154.4, 172.7, 175.8. HRMS (APCI) m/z (neg): 649.4461; $C_{41}H_{61}O_6$ [M-H] (Calculated 649.4468).

**Compound 4d: 28-propanoyl-3-(3′,3′-dimethylglutaryl)betulin**

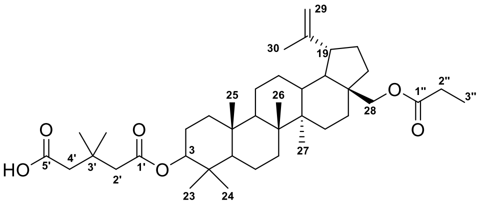


Yield 75%; mp 107-109°C; $R_f$ 0.56 (chloroform/ethanol, 15:1, v/v). $^1$H NMR (600 MHz, $CDCl_3$) δ (ppm): 0.79-0.80 (m, 1H, H-5), 0.85 (s, 3H, $CH_3$-24), 0.86 (s, 3H, $CH_3$-23), 0.87 (s, 3H, $CH_3$-25), 0.98 (s, 3H, $CH_3$-27), 1.06 (s, 3H, $CH_3$-26), 1.16 (m, 6H, 2 x $CH_3$, dimethylglutaric; 3H, $CH_3$-3″), 1.69 (s, 3H, $CH_3$-30), 1.99-0.85 (m, 24, CH, $CH_2$), 2.38-2.51 (m, 4H, 2 x $CH_2$, dimethylglutaric H-2′, H-4′; H-19; 2H, $CH_2$-2″), 3.86 (d, 1H, $^2J$ = 10.8 Hz, H-28), 4.28 (d, 1H, $^2J$ = 11.4 Hz, H-28), 4.49-4.51 (m, 1H, H-3), 4.60 (s, 1H, H-29), 4.70 (s, 1H, H-29). $^{13}$C NMR (150 MHz, $CDCl_3$) δ (ppm): 9.3, 14.3, 14.7, 16.0, 16.1, 16.6, 18.2, 19.1, 20.8, 23.8, 25.1, 27.0, 27.8, 28.0, 29.6, 29.7, 32.6, 34.1, 34.6, 37.0, 37.5, 37.7, 38.3, 40.9, 42.7, 45.2, 45.6, 46.4, 47.7, 48.8, 50.2, 55.4, 60.4, 62.6, 81.4, 109.9, 150.2, 172.7, 175.0, 176.5. HRMS (APCI) m/z (neg): 639.4682; $C_{40}H_{63}O_6$ [M-H] (Calculated 639.4625).

**Compound 4e: 28-(3-cyclopropyl-2-propynoyl)-3-(3′,3′-dimethylglutaryl)betulin**

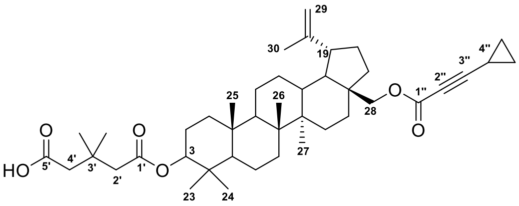


Yield 66%; mp 162-164°C; $R_f$ 0.55 (chloroform/ethanol, 15:1, v/v). $^1$H NMR (600 MHz, $CDCl_3$) δ (ppm): 0.79-0.80 (m, 1H, H-5), 0.85 (s, 3H, $CH_3$-24), 0.86 (s, 3H, $CH_3$-23), 0.87 (s, 3H, $CH_3$-25), 0.94-0.96 (m, 5H, $CH_2$, CH-4″), 0.98 (s, 3H, $CH_3$-27), 1.03 (s, 3H, $CH_3$-26), 1,16 (m, 6H, 2 x $CH_3$, dimethylglutaric), 1.70 (s, 3H, $CH_3$-30), 2.03-0.85 (m, 24H, CH, $CH_2$), 2.40-2.48 (m, 4H, 2 x $CH_2$, dimethylglutaric H-2′, H-4′; H-19), 3.95 (d, 1H, $^2J$ = 10.8 Hz, H-28), 4.33 (d, 1H, $^2J$ = 11.4 Hz, H-28), 4.50-4.52 (m, 1H, H-3), 4.60 (s, 1H, H-29), 4.70 (s, 1H, H-29). $^{13}$C NMR (150 MHz, $CDCl_3$) δ (ppm): -0.5, 9.2, 14.8, 16.0, 16.1, 16.6, 18.2, 19.1, 20.8, 23.8, 25.1, 27.0, 28.0, 28.1, 29.5, 29.6, 29.7, 32.8, 34.1, 34.5, 37.0, 37.6, 37.7, 38.3, 40.9, 42.7, 45.2, 45.6, 46.4, 47.7, 48.8, 50.2, 53.5, 55.4, 64.2, 68.6, 81.6, 93.3, 110.0, 150.0, 154.5, 172.6, 175.9. HRMS (APCI) m/z (neg): 675.4658; $C_{43}H_{63}O_6$ [M-H] (Calculated 675.4625).

**Compound 4f: 28-phenylpropynoyl-3-(3′,3′-dimethylglutaryl)betulin**

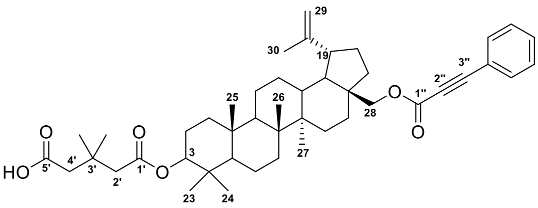

Yield 68%; mp 110-113°C; $R_f$ 0.61 (chloroform/ethanol, 15:1, v/v). $^1$H NMR (600 MHz, $CDCl_3$) δ (ppm): 0.80-0.82 (m, 1H, H-5), 0.87 (s, 3H, $CH_3$-24), 0.88 (s, 6H, 2 x $CH_3$-23,25), 1.00 (s, 3H, $CH_3$-27), 1.07 (s, 3H, $CH_3$-26), 1.16 (m, 6H, 2 x $CH_3$, dimethylglutaric), 1.72 (s, 3H, $CH_3$-30), 2.02-0.87 (m, 24H, CH, $CH_2$), 2.41-2.51 (m, 4H, 2 x $CH_2$, dimethylglutaric H-2′, H-4′; H-19), 4.05 (d, 1H, $^2J$ = 10.8 Hz, H-28), 4.44 (d, 1H, $^2J$ = 10.8 Hz, H-28), 4.52-4.55 (m, 1H, H-3), 4.62 (s, 1H, H-29), 4.72 (s, 1H, H-29), 7.39-7.62 (m, 5H, $H_{Ar}$). $^{13}$C NMR (150 MHz, $CDCl_3$) δ (ppm): 14.8, 16.0, 16.1, 16.6, 18.2, 19.1, 20.8, 23.8, 25.0, 25.1, 27.0, 28.0, 28.2, 28.3, 29.5, 29.6, 33.0, 34.1, 34.6, 37.1, 37.7, 37.7, 38.3, 40.9, 42.7, 45.3, 45.5, 46.5, 47.7, 48.8, 50.2, 53.5, 55.4, 64.6, 80.7, 81.9, 110.0, 119.7, 128.6, 130.6, 133.0, 150.0, 154.8, 174.0. HRMS (APCI) m/z (neg): 711.4603; $C_{46}H_{63}O_6$ [M-H] (Calculated 711.4625).

**Compound 5a: 28-propynoyl-3-(3′,3′-dimethylsuccinyl)betulin**

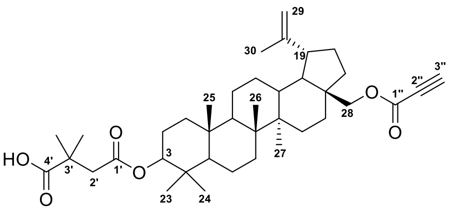

Yield 52%; mp 142-145°C; $R_f$ 0.55 (chloroform/ethanol, 15:1, v/v). $^1$H NMR (600 MHz, $CDCl_3$) δ (ppm): 0.68-0.70 (m, 1H, H-5), 0.74 (s, 3H, $CH_3$-24), 0.76 (s, 6H, 2 x $CH_3$-23, 25), 0.90 (s, 3H, $CH_3$ -27), 0.95 (s, 3H, $CH_3$-26), 1.27 (s, 3H, $CH_3$, dimethylsuccinic), 1.23 (s, 3H, $CH_3$, dimethylsuccinic), 1.59 (s, 3H, $CH_3$), 0.79-2.00 (m, 23H, CH, $CH_2$), 2.32-2.40 (m, 1H, H-19), 2.50 (d, 1H, dimethylsuccinic H-2′ $^2J$=15.6 Hz), 2.60 (d, 1H, dimethylsuccinic H-2′ $^2J$ =15.6 Hz); 2.83 (s, 1H, C≡CH), 3.91 (d, 1H, $J$ = 10.8 Hz, H-28), 4.31 (d, 1H, $^2J$ = 10.8 Hz, H-28), 4.41-4.43 (m, 1H, H-3), 4.53 (s, 1H, H-29), 4.62 (s, 1H, H-29). $^{13}$C NMR (150 MHz, $CDCl_3$) δ (ppm): 14.7, 16.0, 16.1, 16.5, 18.1, 19.1, 20.8, 23.6, 24.7, 25.1, 25.4, 25.6, 27.0, 27.9, 29.4, 29.6, 33.4, 34.1, 34.4, 37.0, 37.7, 37.7, 38.4, 40.4, 40.9, 42.7, 44.7, 46.4, 47.7, 48.8, 50.2, 55.4, 64.9, 74.7, 74.8, 81.5, 110.1, 149.9, 153.3, 171.1, 181.3. HRMS (APCI) m/z (neg): 621.4155; $C_{39}H_{57}O_6$ [M-H] (Calculated 621.4155).

**Compound 5b: 28-pentynoyl-3-(3′,3′-dimethylsuccinyl)betulin**

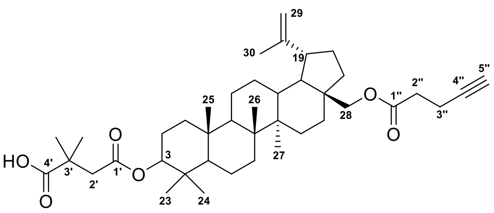

Yield 82%; mp 122-125°C; $R_f$ 0.53 (chloroform/ethanol, 15:1, v/v). $^1$H NMR (600 MHz, $CDCl_3$) δ (ppm): 0.69-0.71 (m, 1H, H-5), 0.74 (s, 3H, $CH_3$-24), 0.76 (s, 6H, 2 x $CH_3$-23, 25), 0.90 (s, 3H, $CH_3$-27), 0.96 (s, 3H, $CH_3$-26), 1.22 (s, 3H, $CH_3$, dimethylsuccinic), 1.23 (s, 3H, $CH_3$, dimethylsuccinic), 1.59 (s, 3H, $CH_3$), 1.91 (t, 1H, CH-5′′ C≡CH, $^4J$ =2.4 Hz ), 0.79-2.00 (m, 23H, CH, $CH_2$), 2.33-2.40 (m, 1H, H-19), 2.41-2.51 (m, 5H, dimethylsuccinic H-2′, $CH_2$-2′′, $CH_2$-3′′), 2.60 (d, 1H, dimethylsuccinic H-2′ $^2J$ =15.6 Hz), 3.81 (d, 1H, $^2J$ = 10.8 Hz, H-28), 4.23 (d, 1H, $^2J$ = 10.8 Hz, H-28), 4.40-4.43 (m, 1H, H-3), 4.52 (br s, 1H, H-29), 4.62 (br s,

1H, H-29). $^{13}$C NMR (150 MHz, $CDCl_3$) δ (ppm): 14.7, 16.0, 16.1, 16.5, 18.1, 19.1, 20.8, 23.6, 24.7, 25.1, 25.4, 25.6, 27.0, 27.9, 29.5, 29.7, 33.4, 33.6, 34.1, 34.5, 37.0, 37.6, 37.7, 38.4, 40.4, 40.9, 42.7, 44.7, 46.4, 47.7, 48.8, 50.2, 55.4, 63.1, 69.1, 81.6, 82.5, 109.9, 150.1, 171.1, 172.2, 181.4. HRMS (APCI) m/z (neg): 649.4460; $C_{41}H_{61}O_6$ [M-H] (Calculated 649.4468).

**Compound 5c: 28-(2-butynoyl)- 3-(3′,3′-dimethylsuccinyl)betulin**

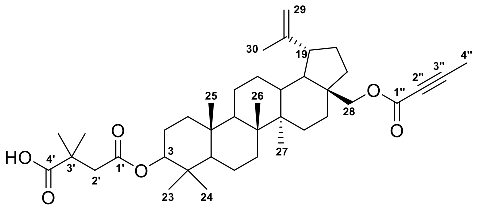

Yield 74%; mp 131-133°C; $R_f$ 0.53 (chloroform/ethanol, 15:1, v/v). $^1$H NMR ($CDCl_3$, 600 MHz) δ (ppm): 0.68-0.69 (m, 1H, H-5), 0.74 (s, 3H, $CH_3$-24), 0.76 (s, 6H, $CH_3$-23, 25), 0.91 (s, 3H, $CH_3$-27), 0.94 (s, 3H, $CH_3$-26), 1.22 (s, 3H, dimethylsuccinic $CH_3$), 1.24 (s, 3H, dimethylsuccinic $CH_3$), 1.61 (s, 3H, $CH_3$-30), 1.90-0.77 (m, 23 H, CH, $CH_2$), 1.93 (s, 3H, $CH_3$-4′′), 2.30-2.39 (m, 1H, H-19), 2.50 (d, 1H, dimethylsuccinic H-2′ $^2J$ =15.6 Hz), 2.60 (d, 1H, dimethylsuccnic H-2′ $^2J$ =15.6 Hz), 3.88 (d, 1H, H-28 $^2J$=10.8 Hz), 4.26 (d, 1H, H-28 $^2J$=10.8 Hz), 4.42 (m, 1H, H-3), 4.51 (s, 1H, H-29), 4.61 (s, 1H, H-29). $^{13}$C NMR (150 MHz, $CDCl_3$) δ (ppm): 3.9, 14.7, 16.0, 16.1, 16.5, 18.1, 19.1, 20.8, 23.6, 24.3, 25.1, 25.0, 25.6, 27.0, 27.9, 29.6, 32.5, 34.1, 34.5, 37.0, 37.6, 37.7, 38.4, 40.4, 40.9, 42.7, 44.7, 46.4, 47.7, 48.8, 50.2, 55.4, 64.3, 72.5, 81.6, 85.6, 110.0, 150.0, 154.4, 171.1, 181.2. HRMS (APCI) m/z (neg): 635.4319; $C_{40}H_{59}O_6$ [M-H] (Calculated 635.4311).

**Compound 5d: 28-propanoyl-3-(3′,3′-dimethylsuccinyl)betulin**

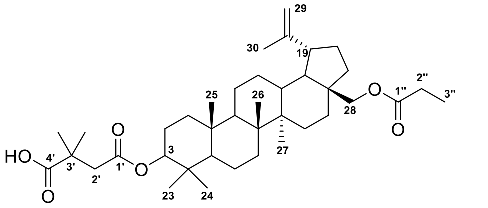

Yield 87%; mp 136-138°C; Rf 0.55 (chloroform/ethanol, 15:1, v/v). $^1$H NMR (CDCl3, 600 MHz) δ (ppm): 0.77-0.79 (m, 1H, H-5), 0.83 (s, 3H, $CH_3$-24), 0.85 (s, 6H, $CH_3$-23, 25), 0.99 (s, 3H, $CH_3$-27), 1.04 (s, 3H, $CH_3$-26), 1.16 (m, 3H, $CH_3$-3′′), 1.33 (s, 3H, dimethylsuccinic $CH_3$), 1.37 (s, 3H, dimethylsuccinic $CH_3$), 1.70 (s, 3H, $CH_3$-30), 0.87-2.00 (m, 23H, CH, $CH_2$), 2.36 (m, 2H, $CH_2$-2′′), 2.44 (m, 1H, H-19), 2.58 (d, 1H, dimethylsuccinic H-2′ $^2J$ =15.6 Hz), 2.69 (d, 1H, dimethylsuccinic H-2′ $^2J$ =15.6 Hz), 3.86 (d, 1H, H-28 $^2J$=10.8 Hz), 4.28 (d, 1H, H-28 $^2J$=10.8 Hz), 4.50 (m, 1H, H-3), 4.60 (s, 1H, H-29), 4.70 (s, 1H, H-29). $^{13}$C NMR (150 MHz, $CDCl_3$) δ (ppm): 9.3, 14.7, 16.0, 16.1, 16.5, 18.1, 19.1, 20.8, 23.6, 25.1, 25.6, 27.7, 27.9, 29.7, 29.7, 32.2, 32.8, 34.1, 34.5, 37.0, 37.5, 37.7, 38.4, 40.9, 42.7, 44.7, 46.5, 47.7, 48.8, 50.3, 55.4, 58.5, 62.6, 81.5, 109.9, 150.2, 171.1, 175.0, 181.4. HRMS (APCI) m/z (neg): 625.4468; $C_{39}H_{61}O_6$ [M-H] (Calculated 625.4430).

**Compound 6a: 28-propynoyl-3-succinylbetulin**

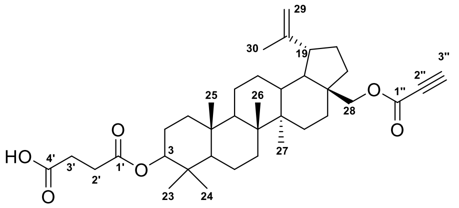

Yield 56%; mp 125-127°C; $R_f$ 0.49 (chloroform/ethanol, 15:1, v/v). $^1$H NMR (600 MHz, $CDCl_3$) δ (ppm): 0.78-0.80 (m, 1H, H-5), 0.84 (s, 3H, $CH_3$), 0.85 (s, 3H, $CH_3$), 0.86 (s, 3H, $CH_3$), 0.99 (s, 3H, $CH_3$), 1.04 (s,

3H, $CH_3$), 1.70 (s, 3H, $CH_3$-30), 0.81-2.00 (m, 23H, CH, $CH_2$), 2.42-2.47 (m, 1H, H-19), 2.42-2.47 (m, 2H, succinic $CH_2$), 2.63-2.67 (m, 2H, succinic $CH_2$), 2.64 (s, 1H, C≡CH, H3''), 4.00 (d, 1H, $^2J$ = 10.8 Hz, H-28), 4.40 (d, 1H, $^2J$ = 10.8 Hz, H-28), 4.50-4.53 (m, 1H, H-3), 4.61 (s, 1H, H-29), 4.71 (s, 1H, H-29). $^{13}$C NMR (150 MHz, $CDCl_3$) δ (ppm): 14.7, 16.0, 16.1, 16.5, 18.1, 19.1, 20.8, 23.6, 24.8, 25.1, 26.9, 27.9, 29.6, 29.7, 34.1, 34.4, 37.0, 37.7, 37.8, 38.4, 40.9, 42.7, 46.4, 47.7, 48.8, 50.2, 55.4, 64.9, 74.7, 74.8, 81.5, 110.1, 149.9, 153.3, 171.9, 176.8. HRMS (APCI) m/z (neg): 594.3845; $C_{37}H_{53}O_6$ [M-H] (Calculated 593.3842).

**Compound 6b: 28-pentynoyl-3-succinylbetulin**

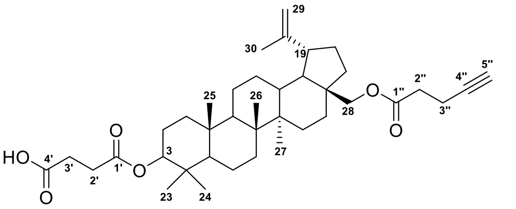


Yield 82%; mp 151-153°C; $R_f$ 0.53 (chloroform/ethanol, 15:1, v/v). $^1$H NMR (600 MHz, $CDCl_3$) δ (ppm): 0.78-82 (m, 1H, H-5), 0.85 (s, 6H, 2 x $CH_3$), 0.86 (s, 3H, $CH_3$), 0.99 (s, 3H, $CH_3$), 1.04 (s, 3H, $CH_3$), 1.70 (s, 3H, $CH_3$-30), 1.99 (t, 1H, CH-5'' C≡CH, $^4J$ =2.4 Hz ); 0.79-2.00 (m, 23H, CH, $CH_2$), 2.43-2.47 (m, 1H, H-19), 2.54-2.70 (m, 8H, succinic H-2', H-3'; $CH_2$-2'', $CH_2$-3''), 3.90-3.91 (d, 1H, $^2J$ = 10.8 Hz, H-28), 4.31-4.35 (d, 1H, $^2J$ = 10.8 Hz, H-28), 4.50-4.53 (m, 1H, H-3), 4.61 (s, 1H, H-29), 4.71 (br s, 1H, H-29). $^{13}$C NMR (150 MHz, $CDCl_3$) δ (ppm): 14.7, 16.0, 16.1, 16.5, 18.1, 19.1, 20.8, 23.6, 25.1, 25.1, 27.0, 27.9, 28.9, 29.3, 29.5, 29.7, 33.5, 34.0, 34.5, 37.0, 37.5, 37.8, 38.3, 40.8, 42.7, 46.4, 47.7, 48.8, 50.2, 55.4, 63.1, 69.1, 81.5, 82.5, 109.9, 150.1, 171.9, 172.2, 177.1. HRMS (APCI) m/z (neg): 621.4158; $C_{39}H_{57}O_6$ [M-H] (Calculated 621.4155).

**Figure S5.** $^1$H NMR, compound **1**

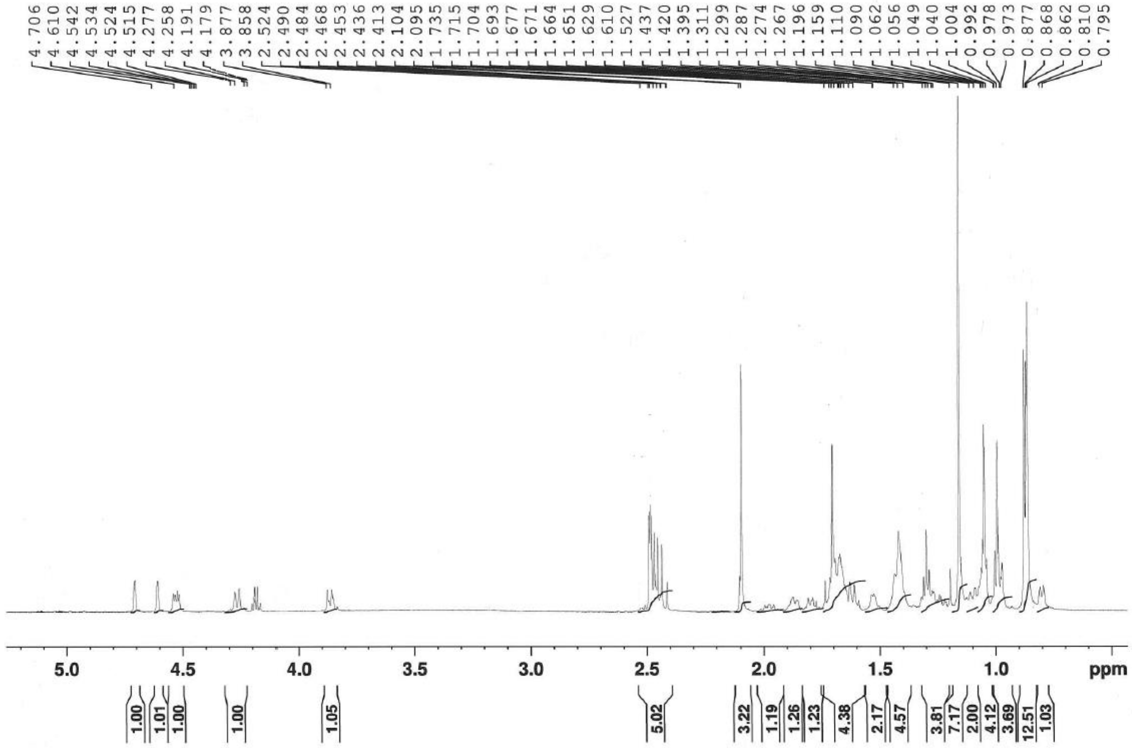

**Figure S6.** $^{13}$C NMR, compound **1**

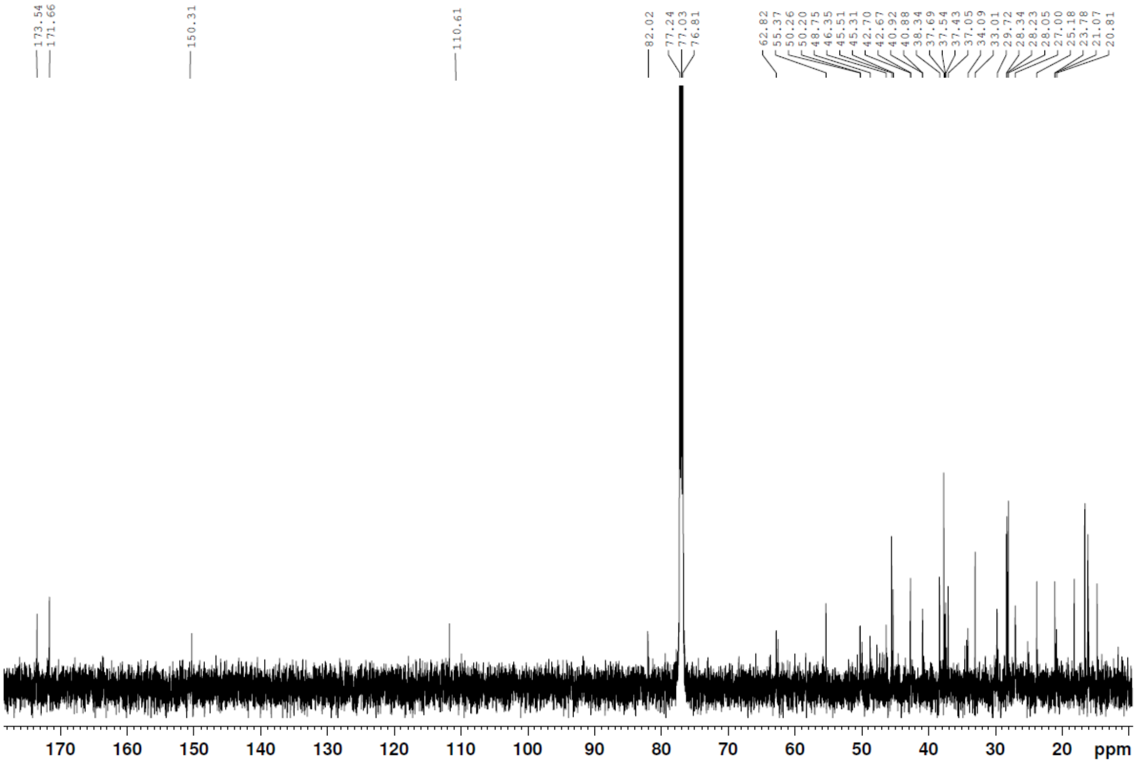


**Figure S7.** HRMS, compound **1**

## Mass Spectrum List Report

**Analysis Info**

| | | | |
|---|---|---|---|
| Analysis Name | D:\Data\EB 320a.d | Acquisition Date | 7/16/2020 11:01:31 AM |
| Method | low_mass_20200619.m | Operator | KM |
| Sample Name | bzt | Instrument | impact II 1825265.10082 |
| Comment | | | |

**Acquisition Parameter**

| | | | | | |
|---|---|---|---|---|---|
| Source Type | APCI | Ion Polarity | Negative | Set Nebulizer | 2.0 Bar |
| Focus | Not active | Set Capillary | 4000 V | Set Dry Heater | 200 °C |
| Scan Begin | 100 m/z | Set End Plate Offset | -500 V | Set Dry Gas | 5.0 l/min |
| Scan End | 2500 m/z | Set Charging Voltage | 2000 V | Set Divert Valve | Source |
| | | Set Corona | 2000 nA | Set APCI Heater | 450 °C |

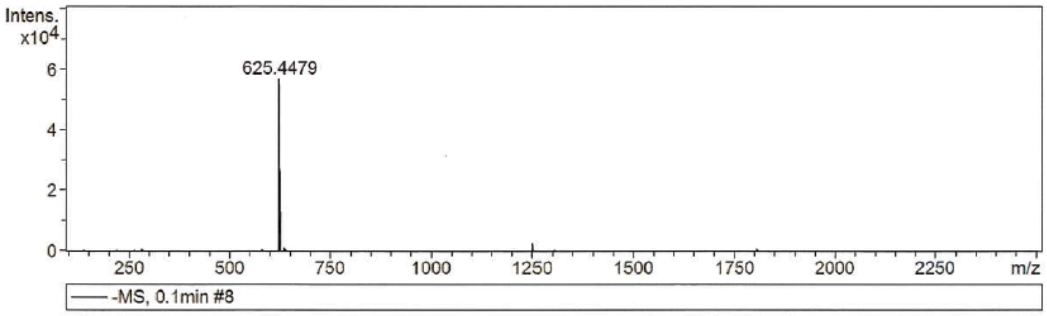


| # | m/z | Res. | S/N | I | I % | FWHM |
|---|---|---|---|---|---|---|
| 1 | 625.4479 | 15810 | 4054.9 | 56759 | 100.0 | 0.0396 |

**Figure S8.** $^{1}$H NMR, compound **2**

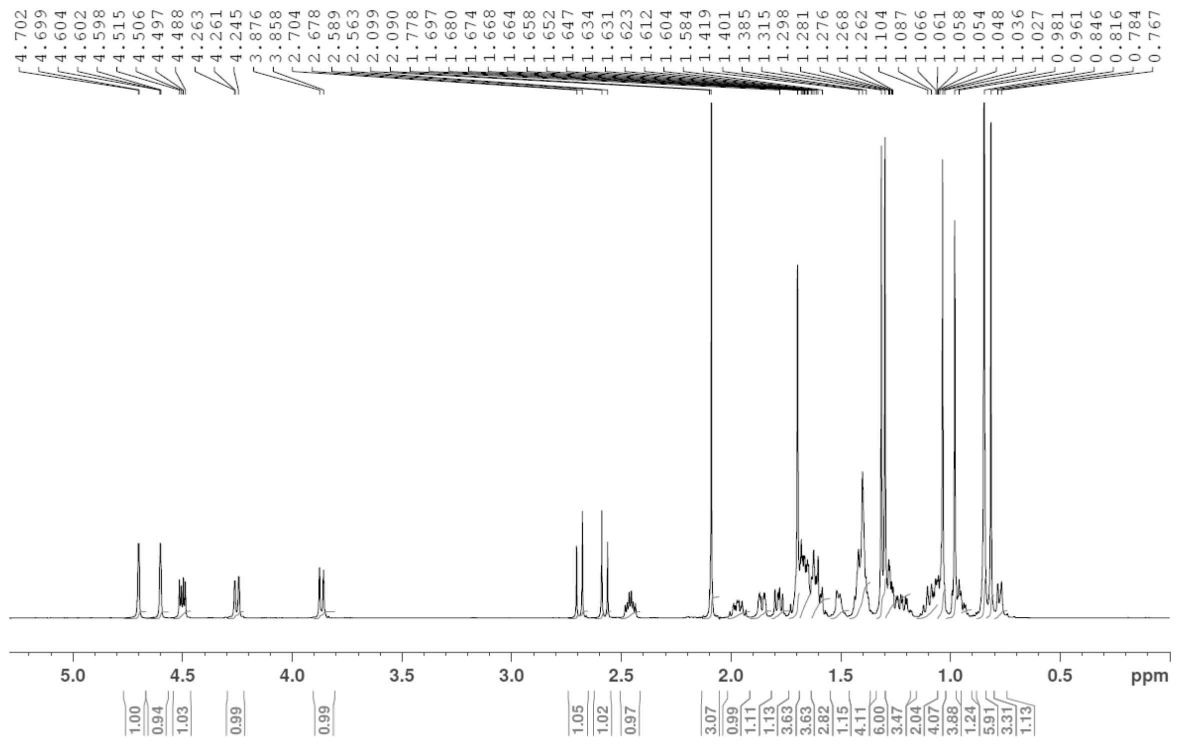


**Figure S9.** $^{13}$C NMR, compound **2**

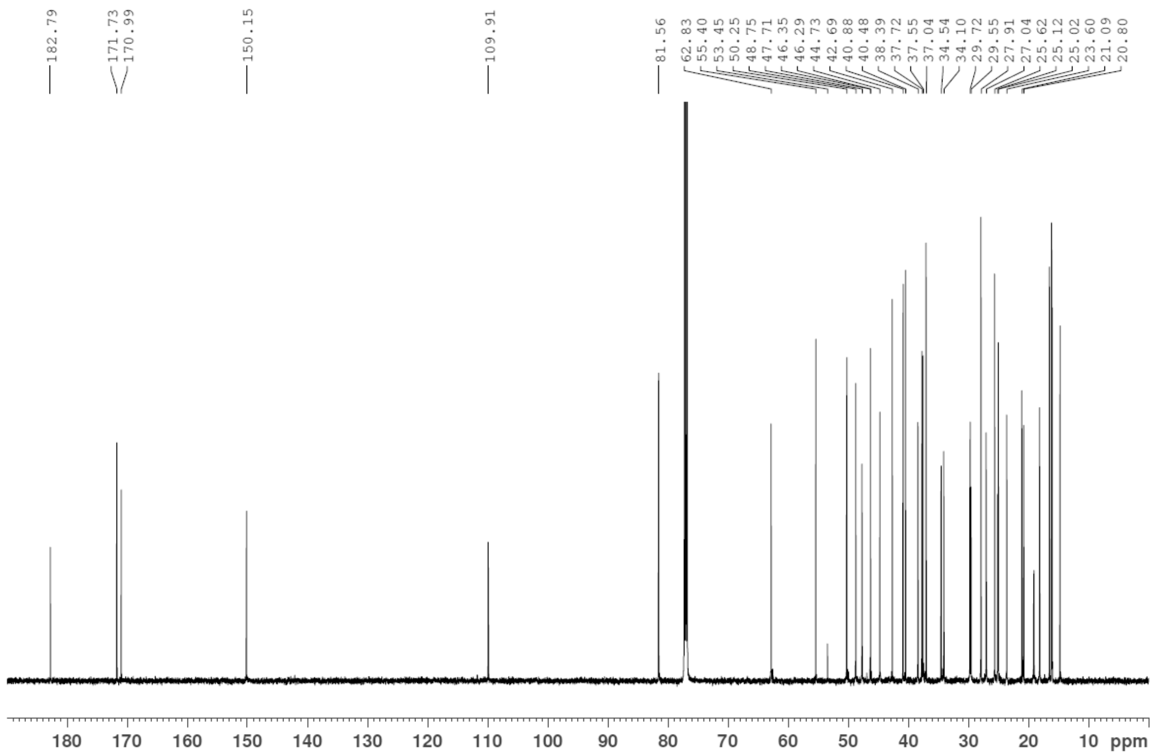

**Figure S10.** HRMS, compound **2**

## Mass Spectrum List Report

**Analysis Info**

| | | | |
|---|---|---|---|
| Analysis Name | D:\Data\ECH308.d | Acquisition Date | 7/19/2023 1:39:06 PM |
| Method | APCI_low_ mass_negative.m | Operator | KM |
| Sample Name | cf3 | Instrument | impact II 1825265.10082 |
| Comment | | | |

**Acquisition Parameter**

| | | | | | |
|---|---|---|---|---|---|
| Source Type | APCI | Ion Polarity | Negative | Set Nebulizer | 2.0 Bar |
| Focus | Active | Set Capillary | 4000 V | Set Dry Heater | 200 °C |
| Scan Begin | 100 m/z | Set End Plate Offset | -500 V | Set Dry Gas | 4.0 l/min |
| Scan End | 1600 m/z | Set Charging Voltage | 2000 V | Set Divert Valve | Source |
| | | Set Corona | 15000 nA | Set APCI Heater | 450 °C |

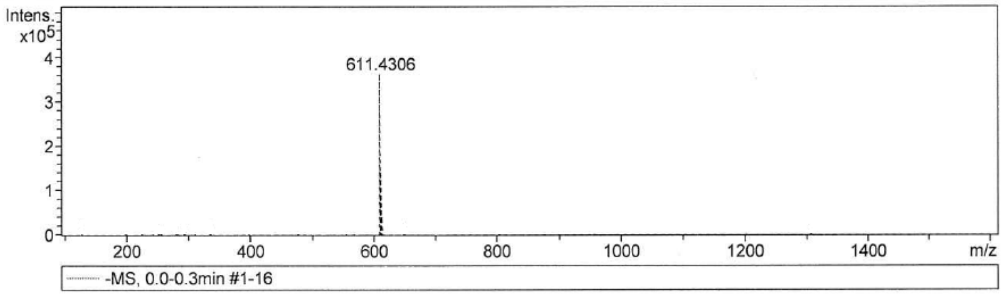


| # | m/z | Res. | S/N | I | I % | FWHM |
|---|---|---|---|---|---|---|
| 1 | 611.4306 | 42065 | 13072.6 | 361816 | 100.0 | 0.0145 |

**Figure S11.** $^{1}$H NMR, compound **3**

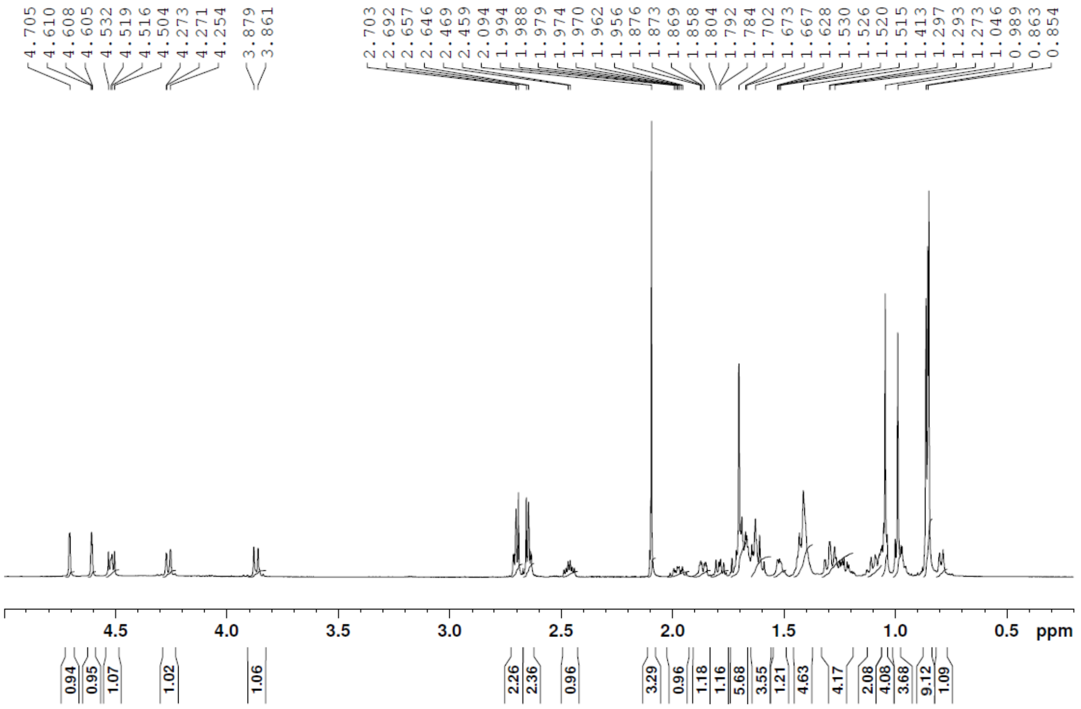

**Figure S12.** $^{13}$C NMR, compound **3**

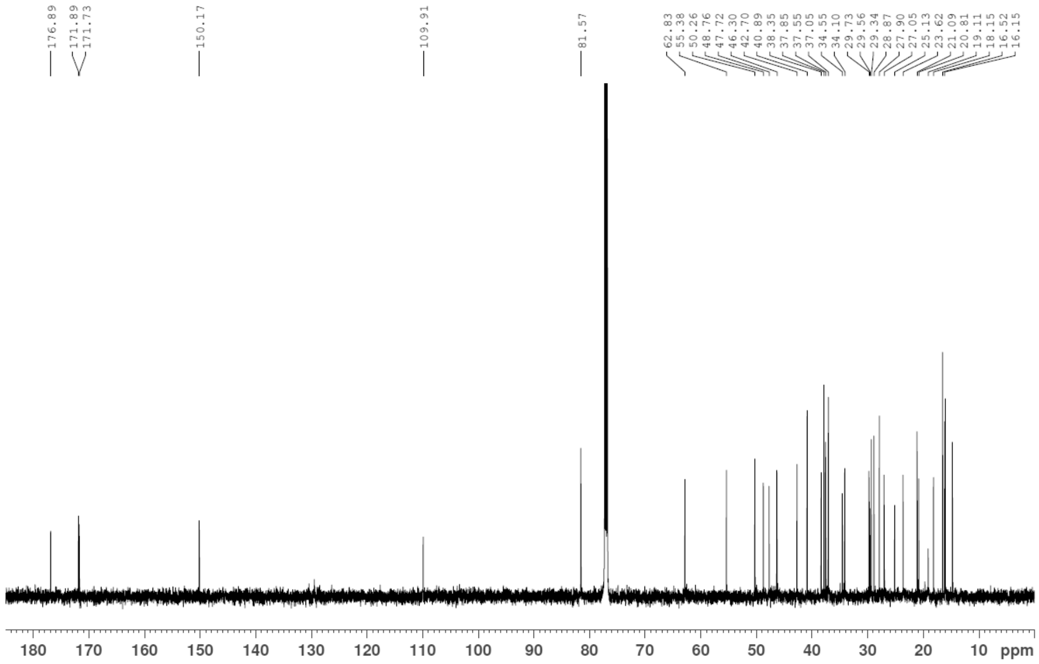


**Figure S13.** HRMS, compound **3**

## Compound Spectrum List Report

**Analysis Info**

Acquisition Date 3/4/2022 11:33:14 AM

Analysis Name D:\Data\ECh 316.d
Method APCI_low_ mass_negative.m
Sample Name 1-tolil
Comment

Operator KM
Instrument impact II 1825265.10082

**Acquisition Parameter**

| | | | | | |
|---|---|---|---|---|---|
| Source Type | APCI | Ion Polarity | Negative | Set Nebulizer | 2.0 Bar |
| Focus | Active | Set Capillary | 4000 V | Set Dry Heater | 200 °C |
| Scan Begin | 100 m/z | Set End Plate Offset | -500 V | Set Dry Gas | 4.0 l/min |
| Scan End | 1600 m/z | Set Charging Voltage | 2000 V | Set Divert Valve | Source |
| | | Set Corona | 15000 nA | Set APCI Heater | 450 °C |

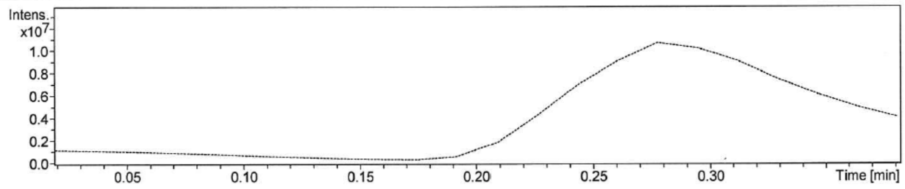


**-MS, 0.3min #16**

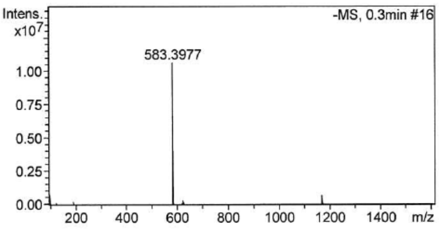


| # | m/z | Res. | S/N | I | I % | FWHM |
|---|---|---|---|---|---|---|
| 1 | 583.3977 | 11159 | 148353.9 | 10634305 | 100.0 | 0.0523 |

**Figure S14.** $^{1}$H NMR, compound **4a**

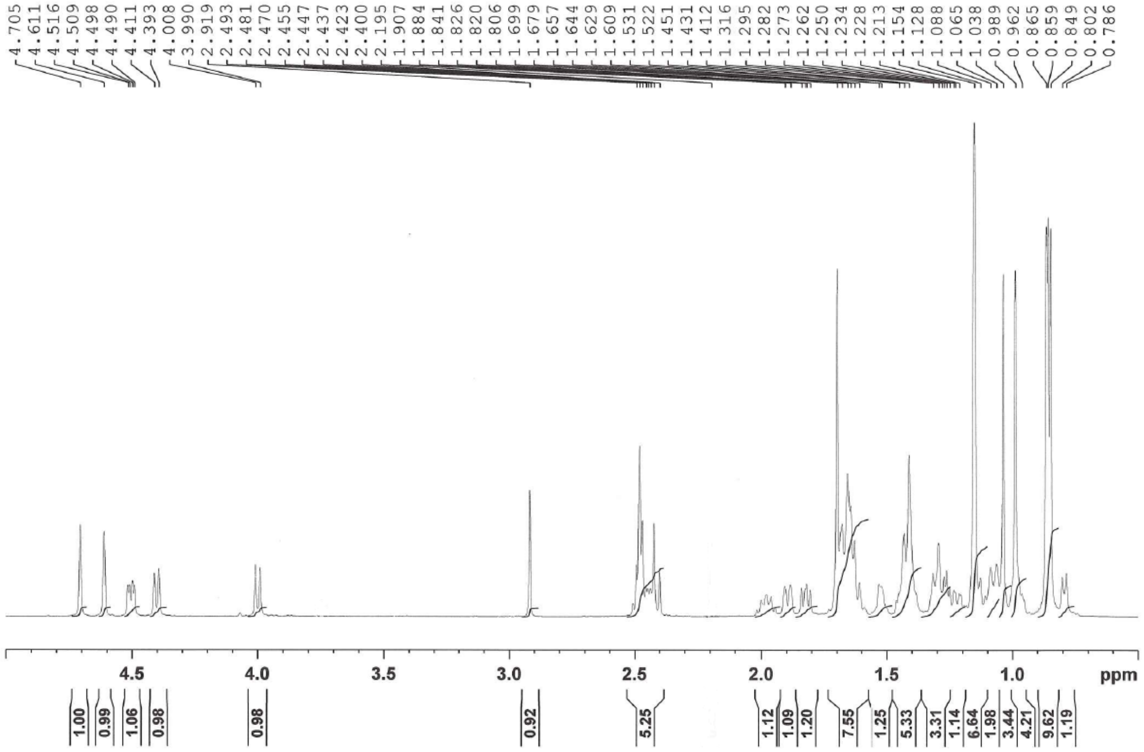


**Figure S15.** $^{13}$C NMR, compound **4a**

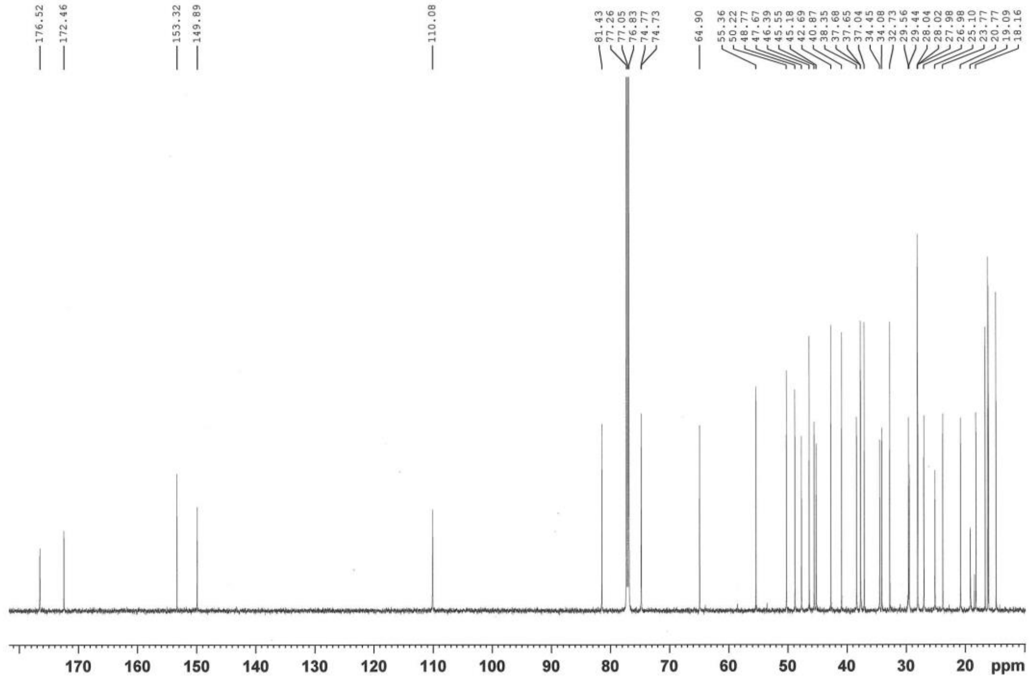

**Figure S16.** HRMS, compound **4a**

## Mass Spectrum List Report

**Analysis Info**

| | | | |
|---|---|---|---|
| Analysis Name | D:\Data\EB 327a.d | Acquisition Date | 7/16/2020 11:09:52 AM |
| Method | low_mass_20200619.m | Operator | KM |
| Sample Name | bzt | Instrument | impact II 1825265.10082 |
| Comment | | | |

**Acquisition Parameter**

| | | | | | |
|---|---|---|---|---|---|
| Source Type | APCI | Ion Polarity | Negative | Set Nebulizer | 2.0 Bar |
| Focus | Not active | Set Capillary | 4000 V | Set Dry Heater | 200 °C |
| Scan Begin | 100 m/z | Set End Plate Offset | -500 V | Set Dry Gas | 5.0 l/min |
| Scan End | 2500 m/z | Set Charging Voltage | 2000 V | Set Divert Valve | Source |
| | | Set Corona | 2000 nA | Set APCI Heater | 450 °C |

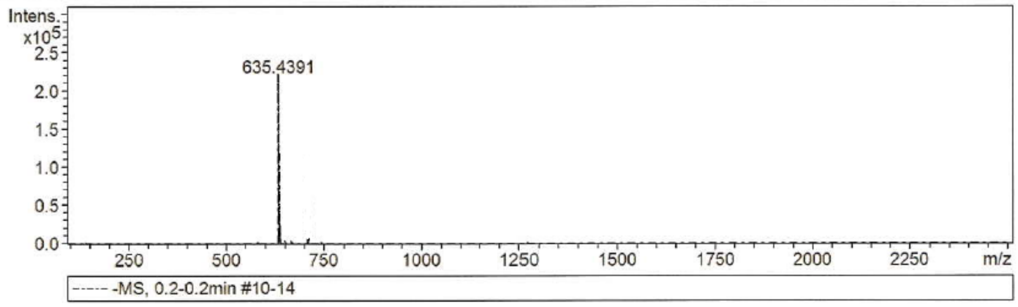


| # | m/z | Res. | S/N | I | I % | FWHM |
|---|---|---|---|---|---|---|
| 1 | 635.4391 | 17656 | 6143.2 | 217247 | 100.0 | 0.0360 |

**Figure S17.** $^{1}$H NMR, compound **4b**

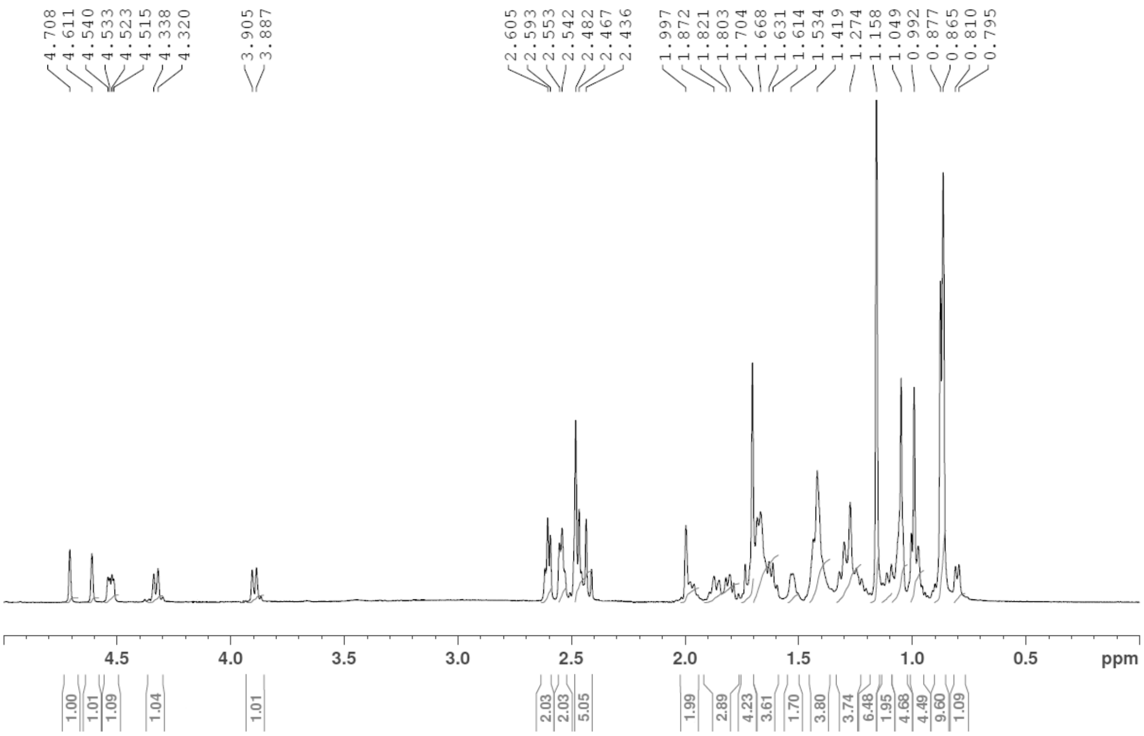

**Figure S18.** $^{13}C$ NMR, compound **4b**

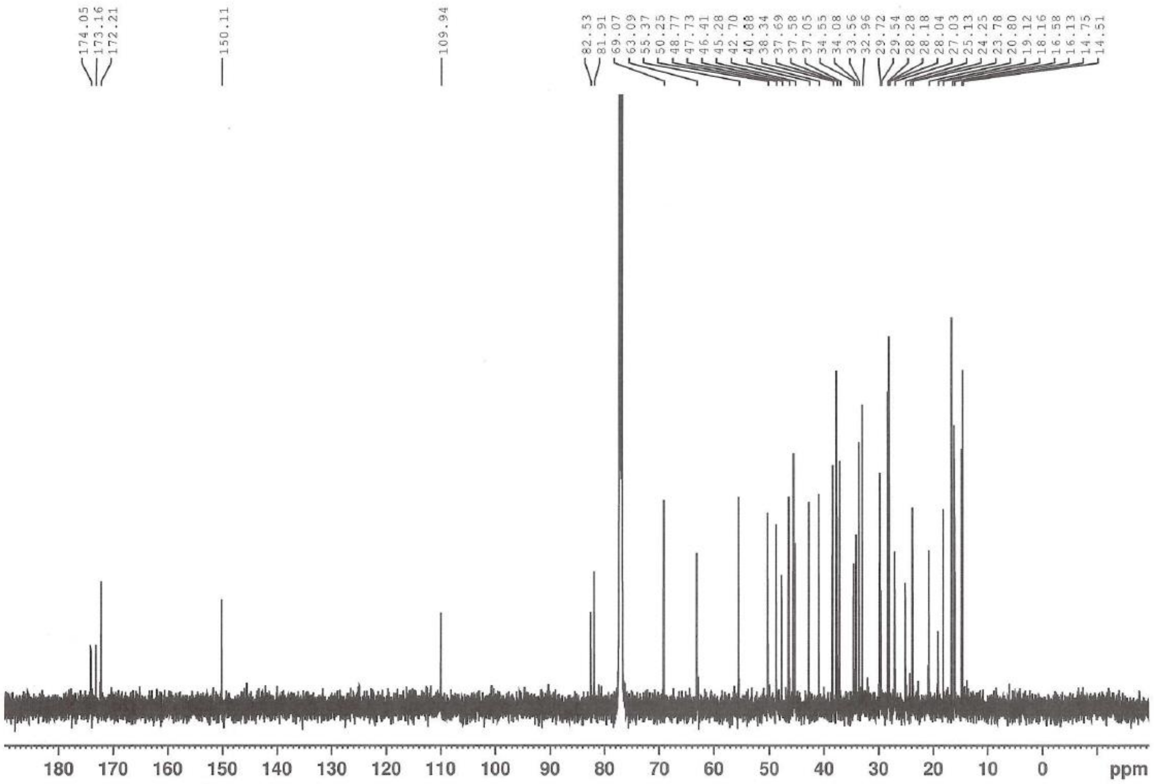


**Figure S19.** HRMS, compound **4b**

## Mass Spectrum List Report

**Analysis Info**

| | | | |
|---|---|---|---|
| Analysis Name | D:\Data\EB 341a.d | Acquisition Date | 7/16/2020 11:31:24 AM |
| Method | low_mass_20200619.m | Operator | KM |
| Sample Name | bzt | Instrument | impact II 1825265.10082 |
| Comment | | | |

**Acquisition Parameter**

| | | | | | |
|---|---|---|---|---|---|
| Source Type | APCI | Ion Polarity | Negative | Set Nebulizer | 2.0 Bar |
| Focus | Not active | Set Capillary | 4000 V | Set Dry Heater | 200 °C |
| Scan Begin | 100 m/z | Set End Plate Offset | -500 V | Set Dry Gas | 5.0 l/min |
| Scan End | 2500 m/z | Set Charging Voltage | 2000 V | Set Divert Valve | Source |
| | | Set Corona | 2000 nA | Set APCI Heater | 450 °C |

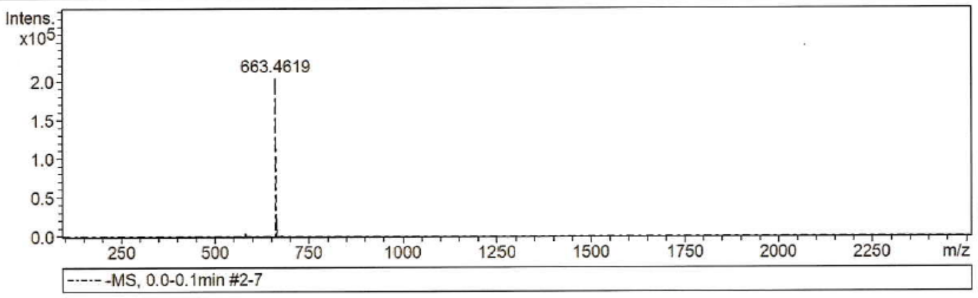


| # | m/z | Res. | S/N | I | I % | FWHM |
|---|---|---|---|---|---|---|
| 1 | 663.4619 | 17638 | 8478.1 | 206084 | 100.0 | 0.0376 |

**Figure S20.** $^{1}$H NMR, compound **4c**

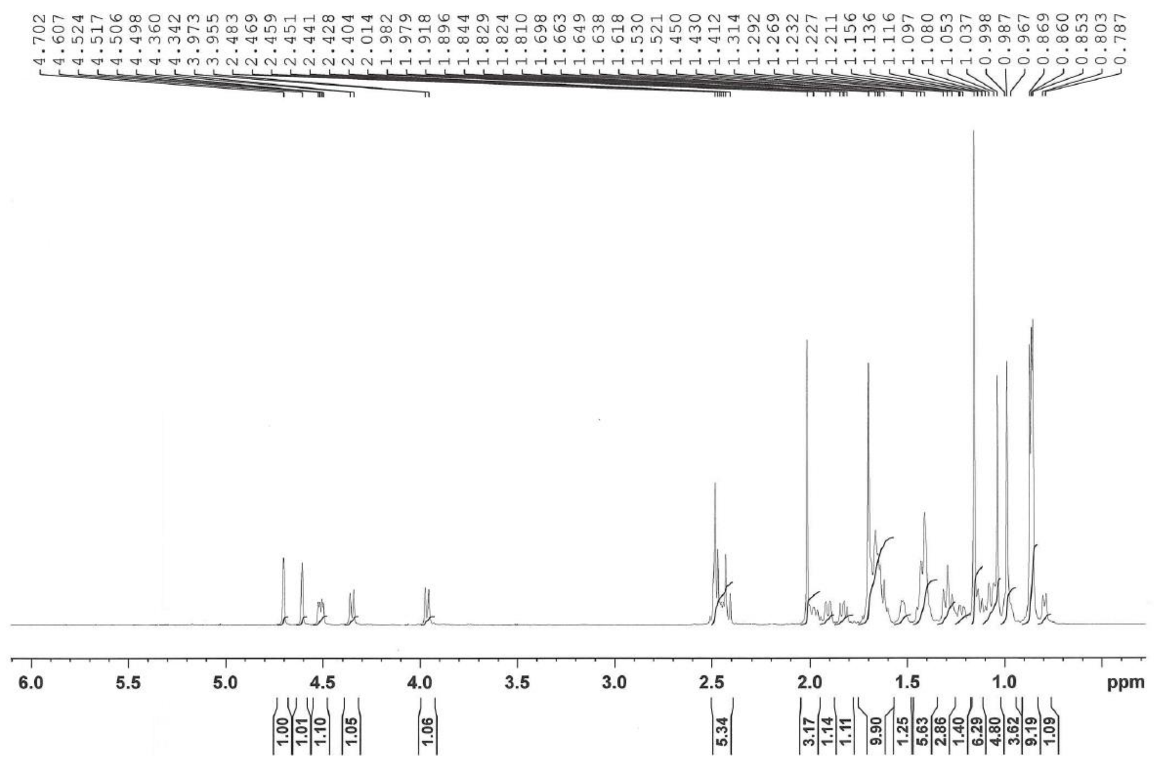

**Figure S21.** $^{13}$C NMR, compound **4c**

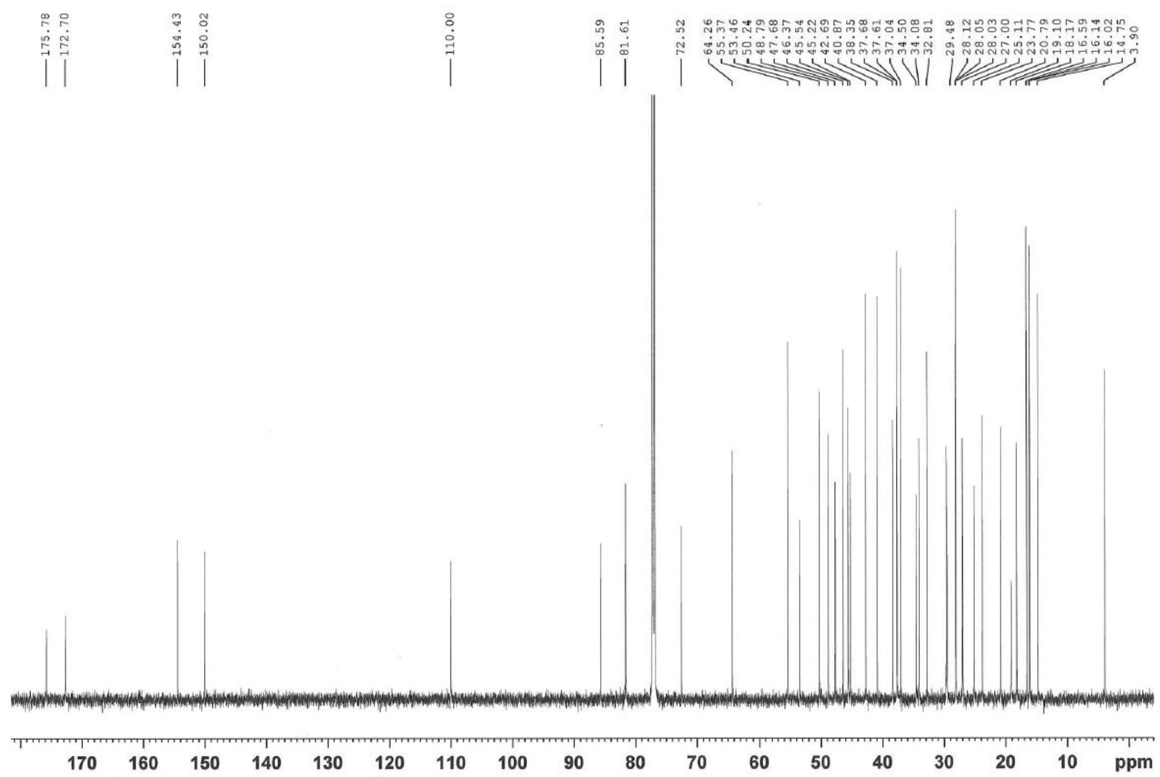

**Figure S22.** HRMS, compound **4c**

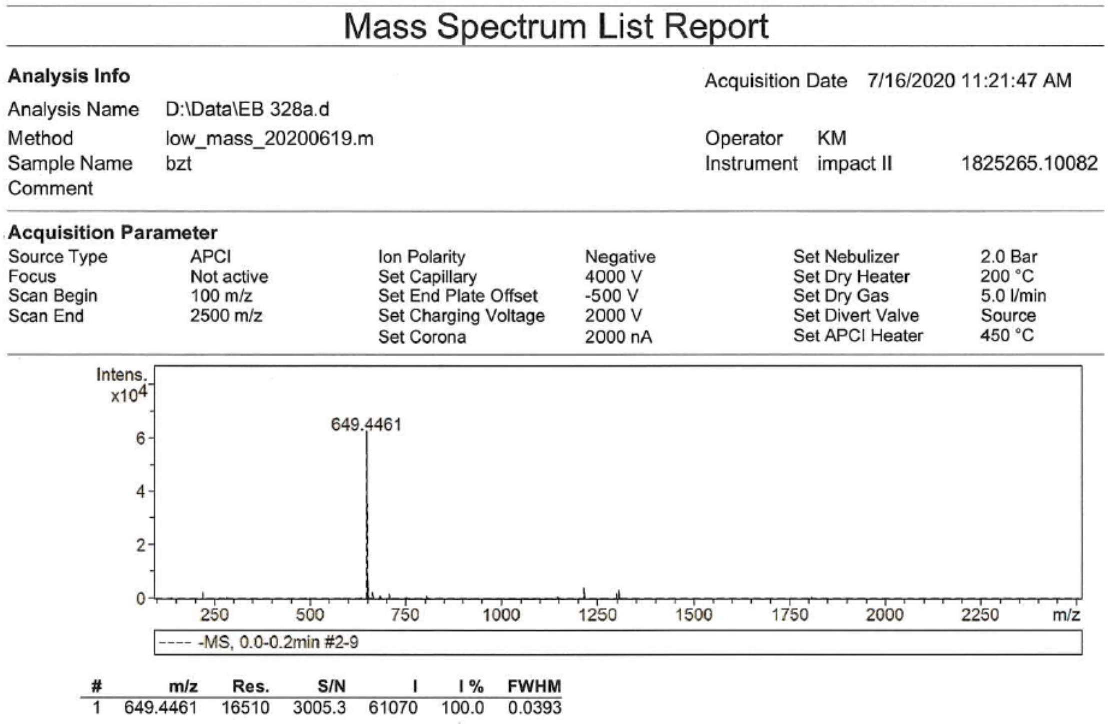

Mass Spectrum List Report

**Analysis Info**

Acquisition Date 7/16/2020 11:21:47 AM

Analysis Name D:\Data\EB 328a.d
Method low_mass_20200619.m
Sample Name bzt
Comment

Operator KM
Instrument impact II 1825265.10082

**Acquisition Parameter**

| | | | | | |
|---|---|---|---|---|---|
| Source Type | APCI | Ion Polarity | Negative | Set Nebulizer | 2.0 Bar |
| Focus | Not active | Set Capillary | 4000 V | Set Dry Heater | 200 °C |
| Scan Begin | 100 m/z | Set End Plate Offset | -500 V | Set Dry Gas | 5.0 l/min |
| Scan End | 2500 m/z | Set Charging Voltage | 2000 V | Set Divert Valve | Source |
| | | Set Corona | 2000 nA | Set APCI Heater | 450 °C |



| # | m/z | Res. | S/N | I | I % | FWHM |
|---|---|---|---|---|---|---|
| 1 | 649.4461 | 16510 | 3005.3 | 61070 | 100.0 | 0.0393 |

**Figure S23.** $^{1}$H NMR, compound **4d**

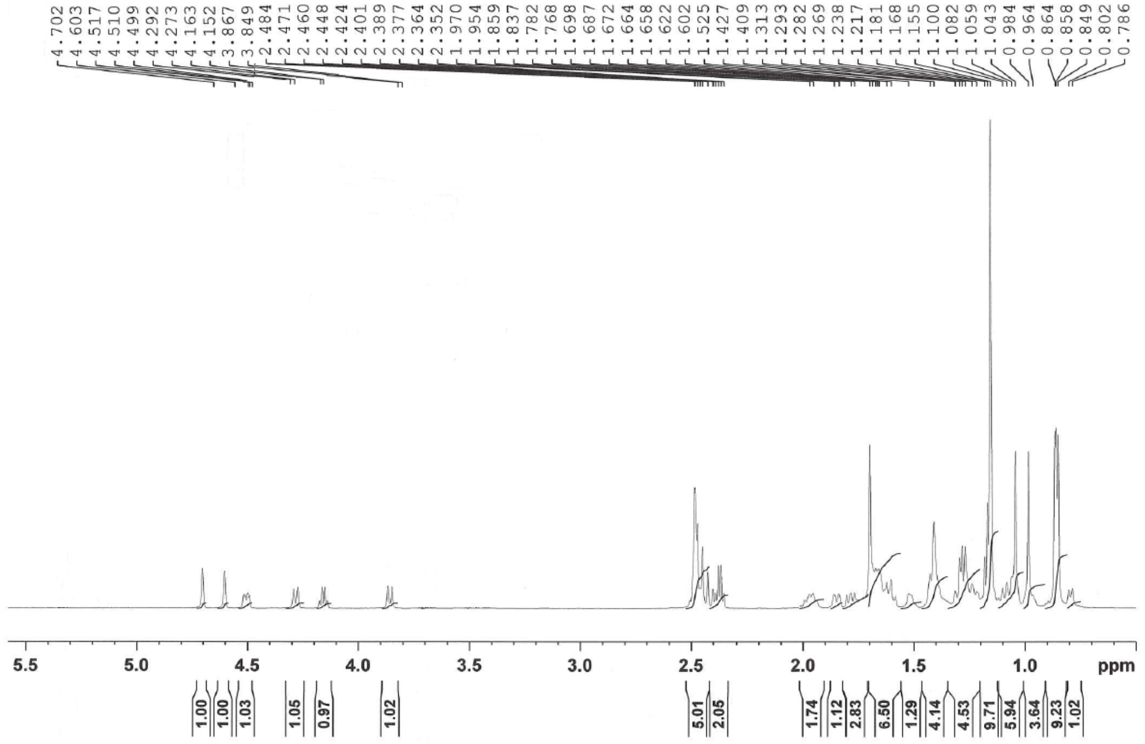

**Figure S24.** $^{13}$C NMR, compound **4d**

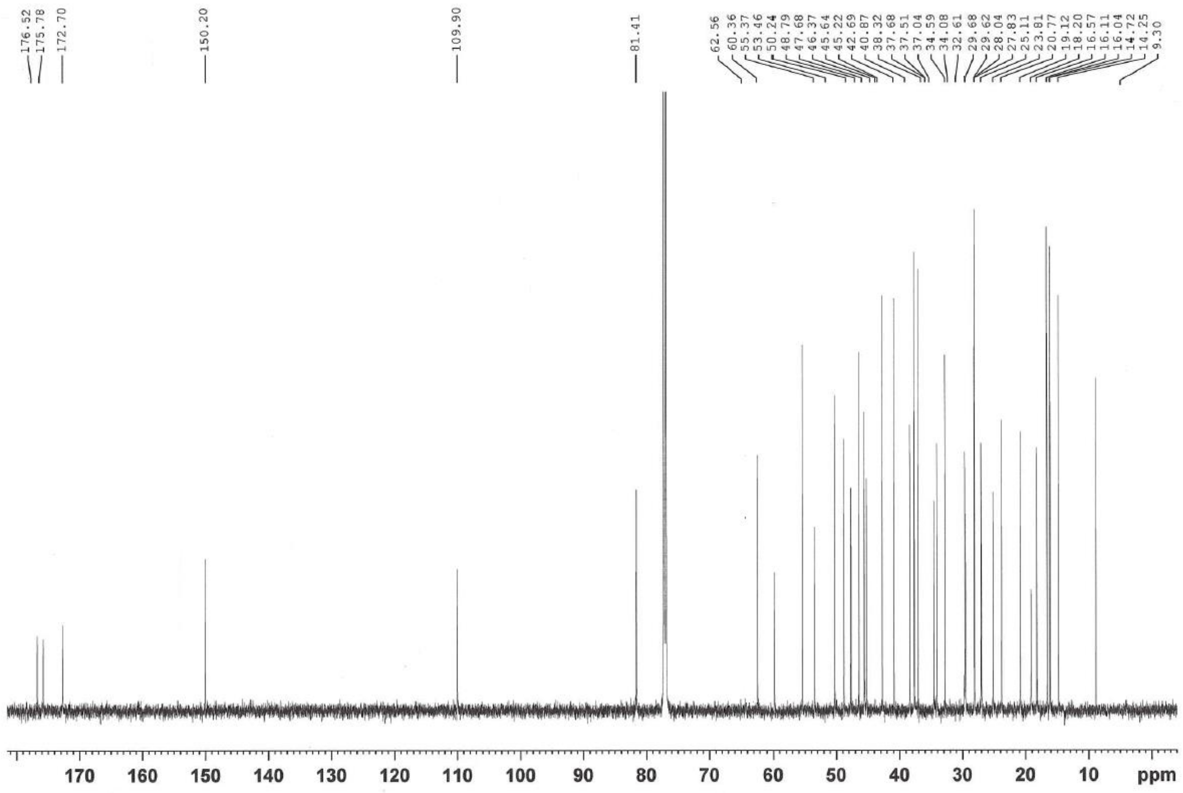


**Figure S25.** HRMS, compound **4d**

## Mass Spectrum List Report

**Analysis Info** Acquisition Date 7/16/2020 10:57:51 AM

| | | | | |
|---|---|---|---|---|
| Analysis Name | D:\Data\EB 330.d | | | |
| Method | low_mass_20200619.m | Operator | KM | |
| Sample Name | bzt | Instrument | impact II | 1825265.10082 |
| Comment | | | | |

**Acquisition Parameter**

| | | | | | |
|---|---|---|---|---|---|
| Source Type | APCI | Ion Polarity | Negative | Set Nebulizer | 2.0 Bar |
| Focus | Not active | Set Capillary | 4000 V | Set Dry Heater | 200 °C |
| Scan Begin | 100 m/z | Set End Plate Offset | -500 V | Set Dry Gas | 5.0 l/min |
| Scan End | 2500 m/z | Set Charging Voltage | 2000 V | Set Divert Valve | Source |
| | | Set Corona | 2000 nA | Set APCI Heater | 450 °C |

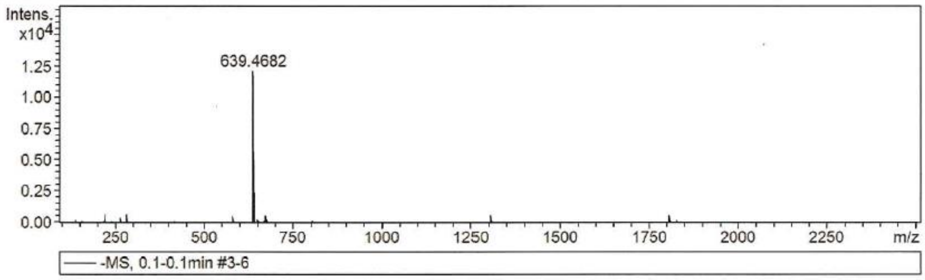


| # | m/z | Res. | S/N | I | I % | FWHM |
|---|---|---|---|---|---|---|
| 1 | 639.4682 | 18228 | 827.1 | 12136 | 100.0 | 0.0351 |

**Figure S26.** $^{1}$H NMR, compound **4e**

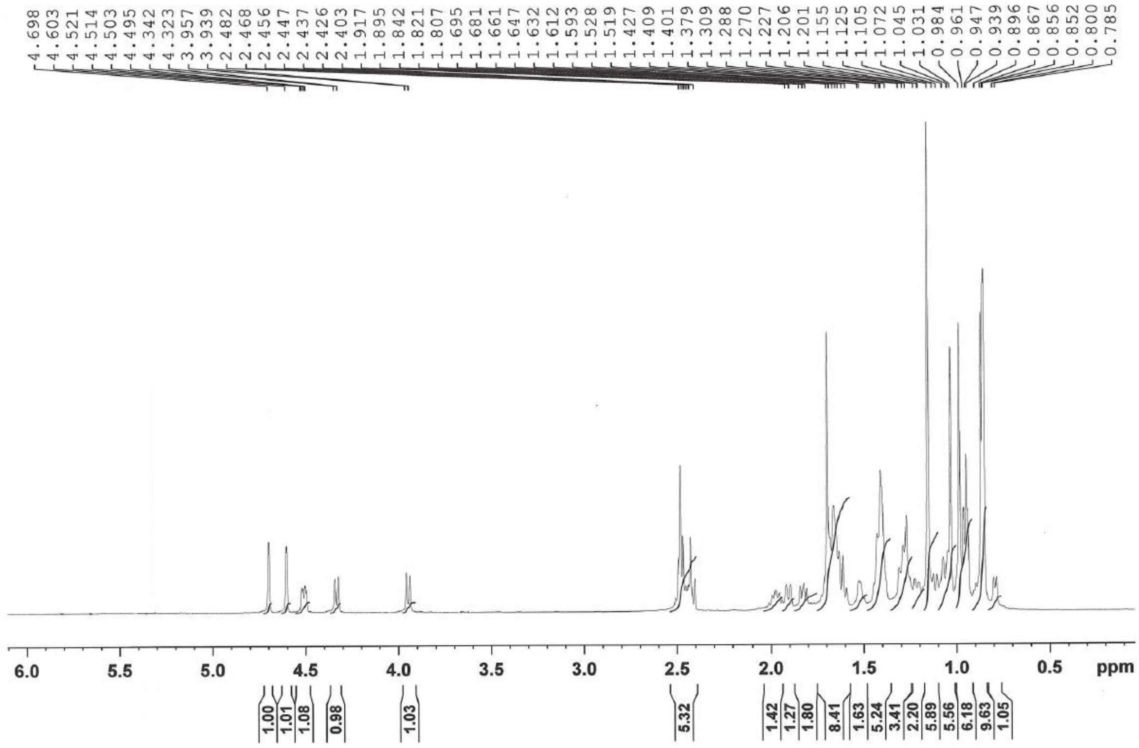


**Figure S27.** $^{13}$C NMR, compound **4e**

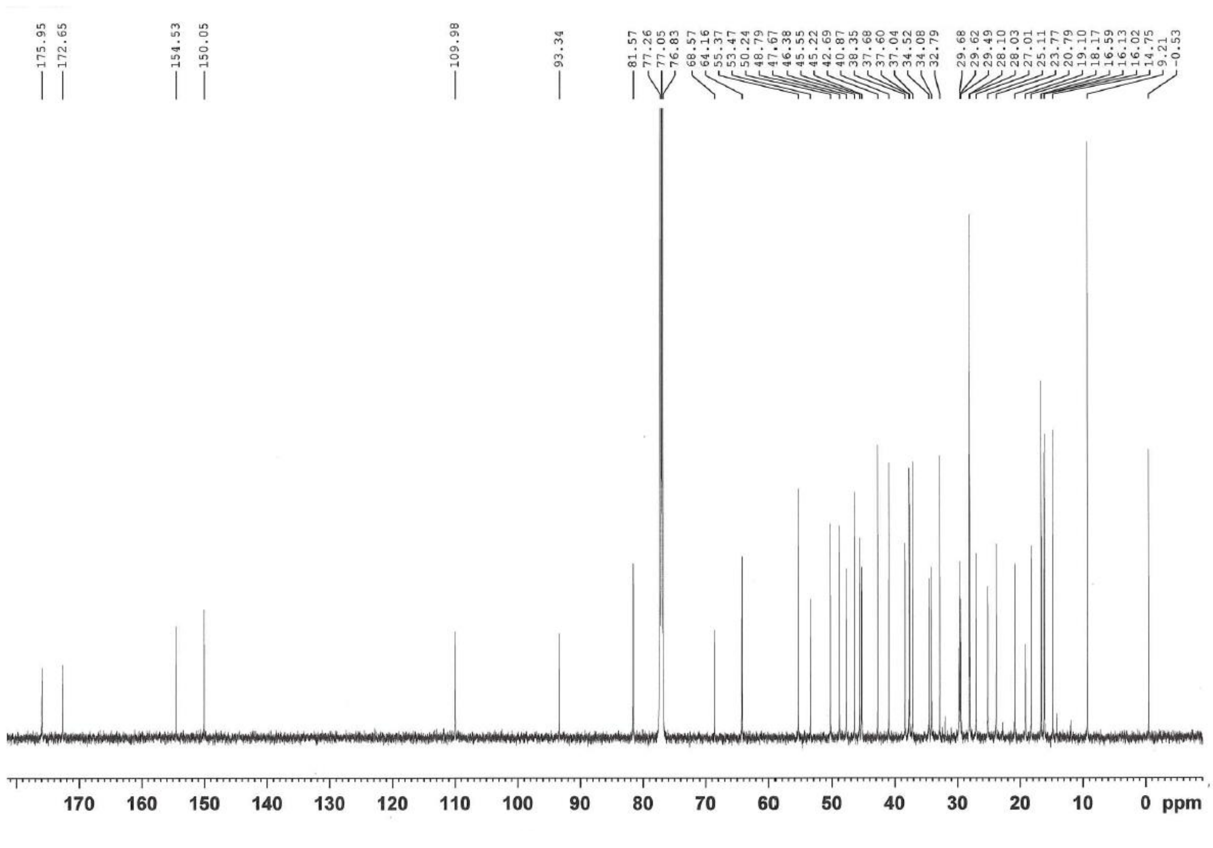

**Figure S28.** HRMS, compound **4e**

## Mass Spectrum List Report

**Analysis Info**

Analysis Name D:\Data\EB 329.d
Method low_mass_20200619.m
Sample Name bzt
Comment

Acquisition Date 7/16/2020 10:53:41 AM
Operator KM
Instrument impact II 1825265.10082

**Acquisition Parameter**

| | | | | | |
|---|---|---|---|---|---|
| Source Type | APCI | Ion Polarity | Negative | Set Nebulizer | 2.0 Bar |
| Focus | Not active | Set Capillary | 4000 V | Set Dry Heater | 200 °C |
| Scan Begin | 100 m/z | Set End Plate Offset | -500 V | Set Dry Gas | 5.0 l/min |
| Scan End | 2500 m/z | Set Charging Voltage | 2000 V | Set Divert Valve | Source |
| | | Set Corona | 2000 nA | Set APCI Heater | 450 °C |

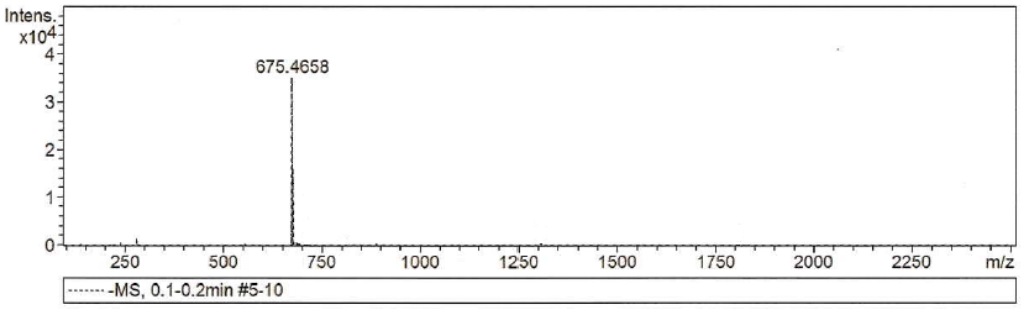


| # | m/z | Res. | S/N | I | I % | FWHM |
|---|---|---|---|---|---|---|
| 1 | 675.4658 | 18234 | 1799.0 | 35097 | 100.0 | 0.0370 |

**Figure S29.** $^1$H NMR, compound **4f**

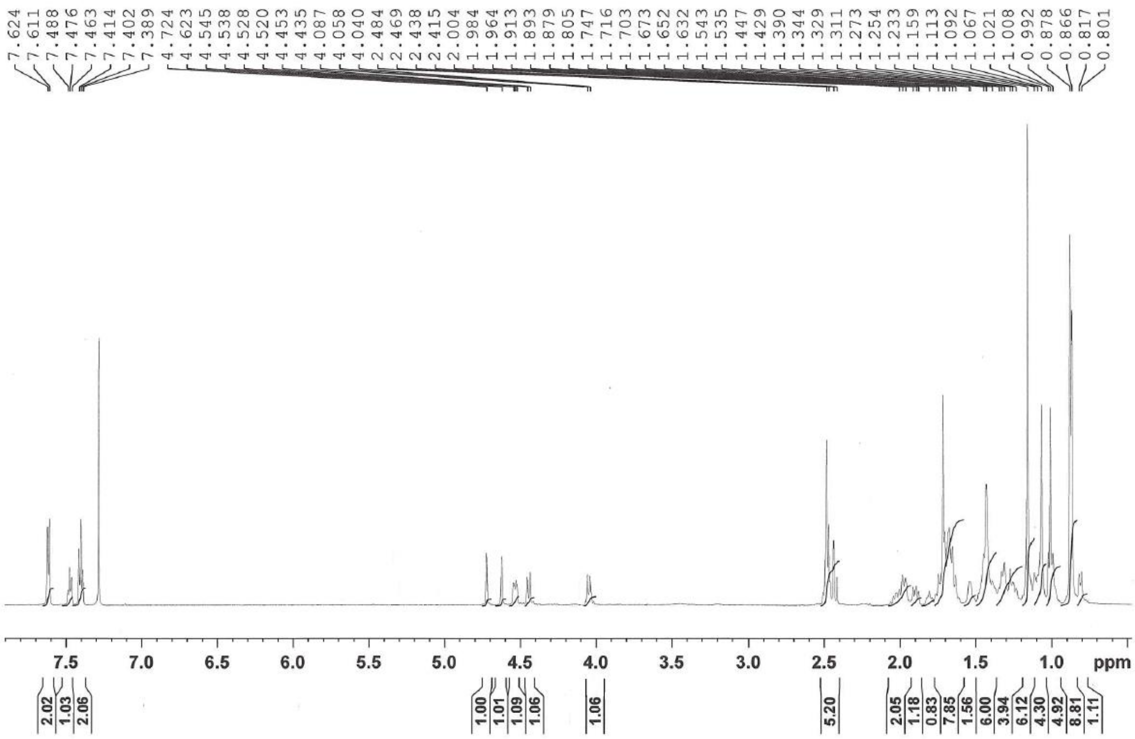

**Figure S30.** $^{13}$C NMR, compound **4f**

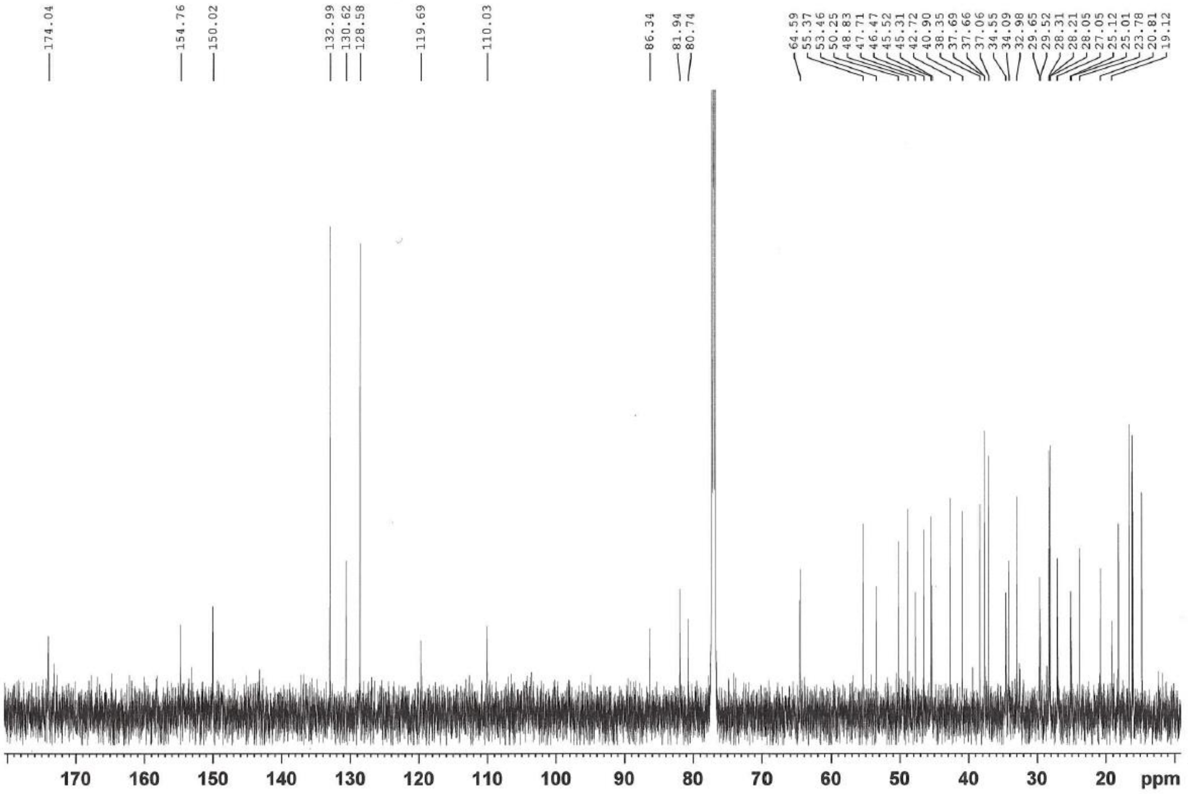


**Figure S31.** HRMS, compound **4f**

## Mass Spectrum List Report

**Analysis Info**

| | | | |
|---|---|---|---|
| Analysis Name | D:\Data\EB 331.d | Acquisition Date | 7/16/2020 11:05:40 AM |
| Method | low_mass_20200619.m | Operator | KM |
| Sample Name | bzt | Instrument | impact II 1825265.10082 |
| Comment | | | |

**Acquisition Parameter**

| | | | | | |
|---|---|---|---|---|---|
| Source Type | APCI | Ion Polarity | Negative | Set Nebulizer | 2.0 Bar |
| Focus | Not active | Set Capillary | 4000 V | Set Dry Heater | 200 °C |
| Scan Begin | 100 m/z | Set End Plate Offset | -500 V | Set Dry Gas | 5.0 l/min |
| Scan End | 2500 m/z | Set Charging Voltage | 2000 V | Set Divert Valve | Source |
| | | Set Corona | 2000 nA | Set APCI Heater | 450 °C |

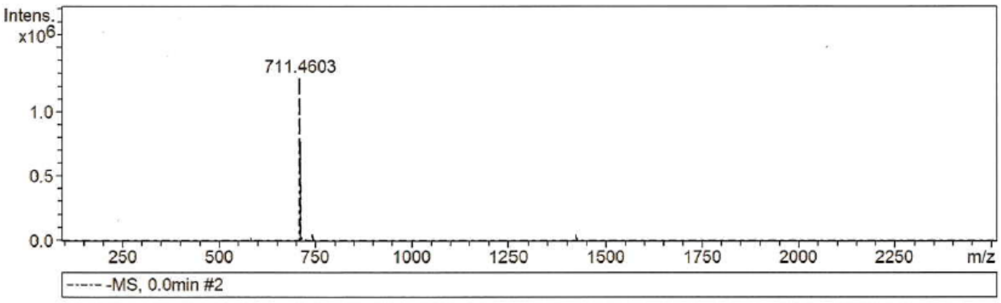


| # | m/z | Res. | S/N | I | I % | FWHM |
|---|---|---|---|---|---|---|
| 1 | 711.4603 | 15215 | 26450.4 | 1272304 | 100.0 | 0.0468 |

**Figure S32.** $^{1}$H NMR, compound **5a**

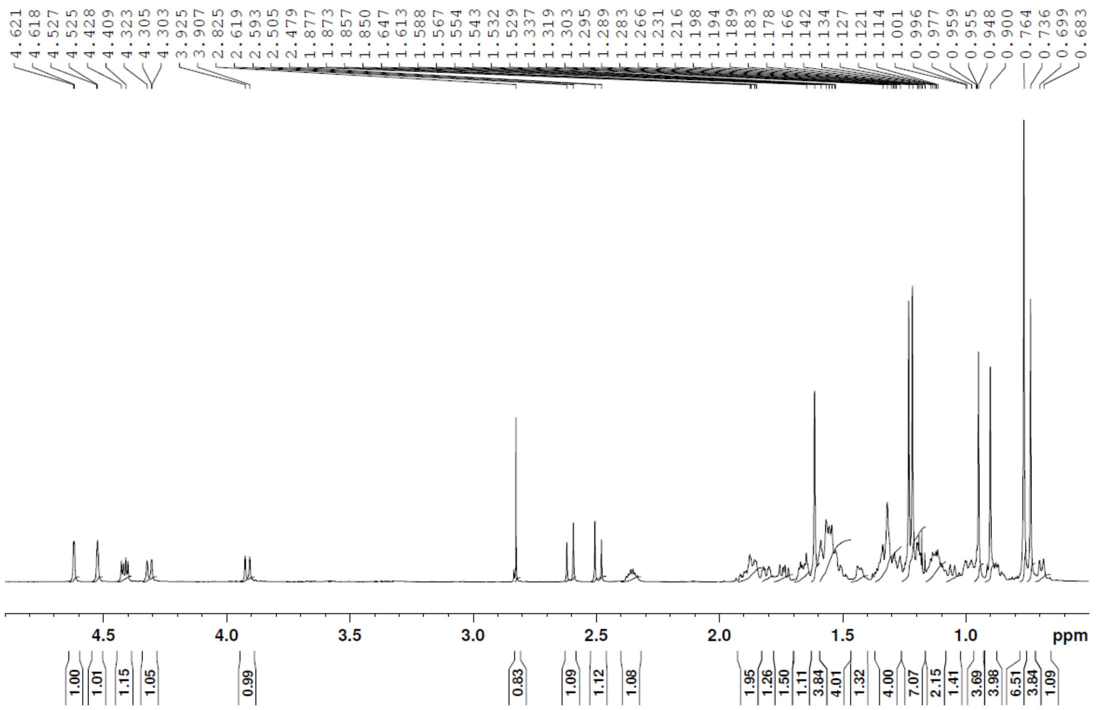


**Figure S33.** $^{13}$C NMR, compound **5a**

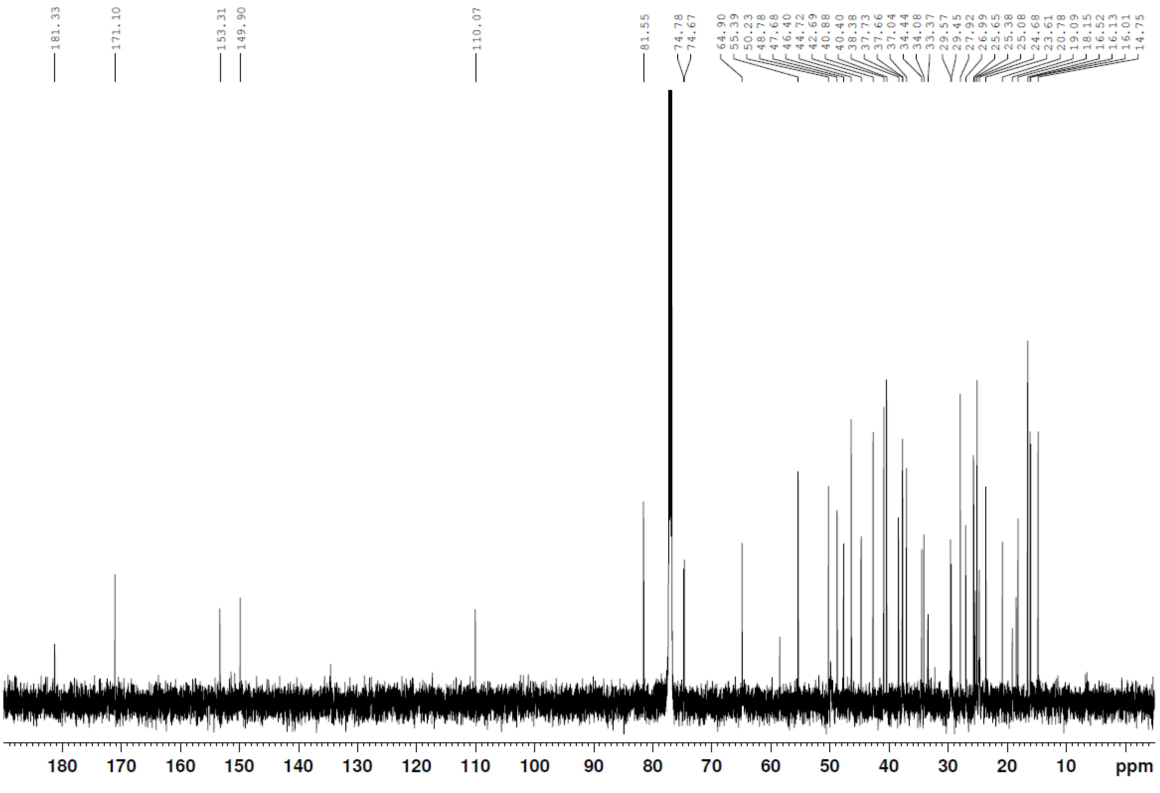

**Figure S34.** HRMS, compound **5a**

## Mass Spectrum List Report

**Analysis Info**

| | | | |
|---|---|---|---|
| Analysis Name | D:\Data\ECH311A_2.d | Acquisition Date | 11/26/2024 11:54:21 AM |
| Method | APCI_low_mass_negative.m | Operator | KM |
| Sample Name | Tune_neg_high_post | Instrument | impact II 1825265.10082 |
| Comment | | | |

**Acquisition Parameter**

| | | | | | |
|---|---|---|---|---|---|
| Source Type | APCI | Ion Polarity | Negative | Set Nebulizer | 2.0 Bar |
| Focus | Active | Set Capillary | 4000 V | Set Dry Heater | 200 °C |
| Scan Begin | 100 m/z | Set End Plate Offset | -500 V | Set Dry Gas | 4.0 l/min |
| Scan End | 1600 m/z | Set Charging Voltage | 2000 V | Set Divert Valve | Source |
| | | Set Corona | 15000 nA | Set APCI Heater | 450 °C |

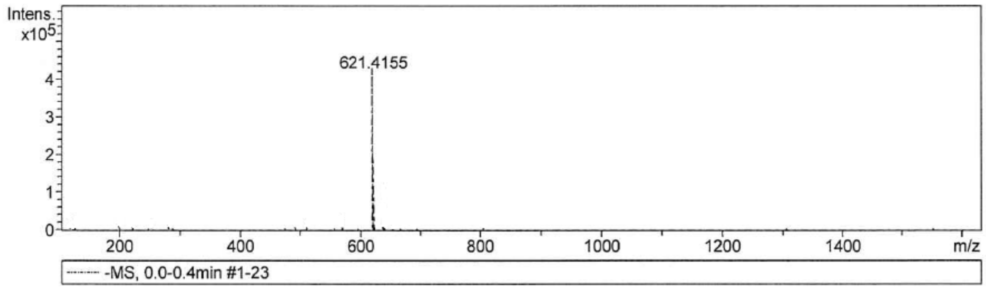


| # | m/z | Res. | S/N | I | I % | FWHM |
|---|---|---|---|---|---|---|
| 1 | 621.4155 | 28690 | 19108.7 | 418365 | 100.0 | 0.0217 |

**Figure S35.** $^{1}$H NMR, compound **5b**

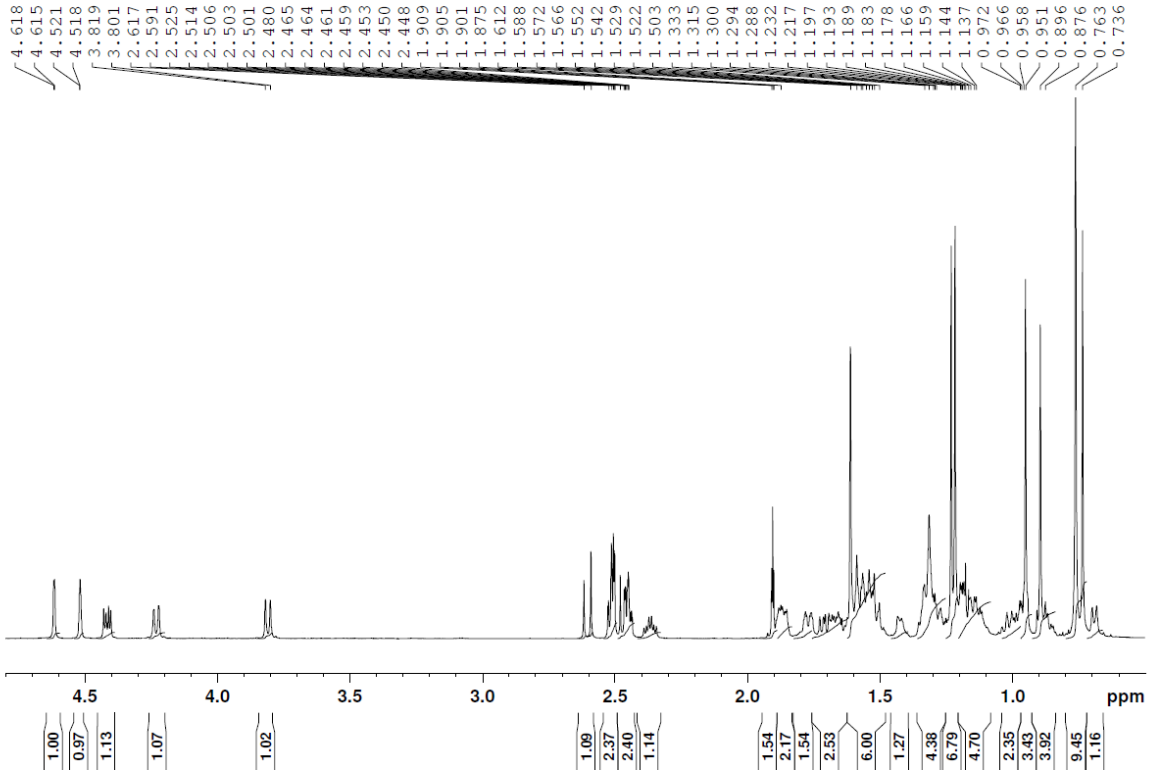

**Figure S36.** $^{13}$C NMR, compound **5b**

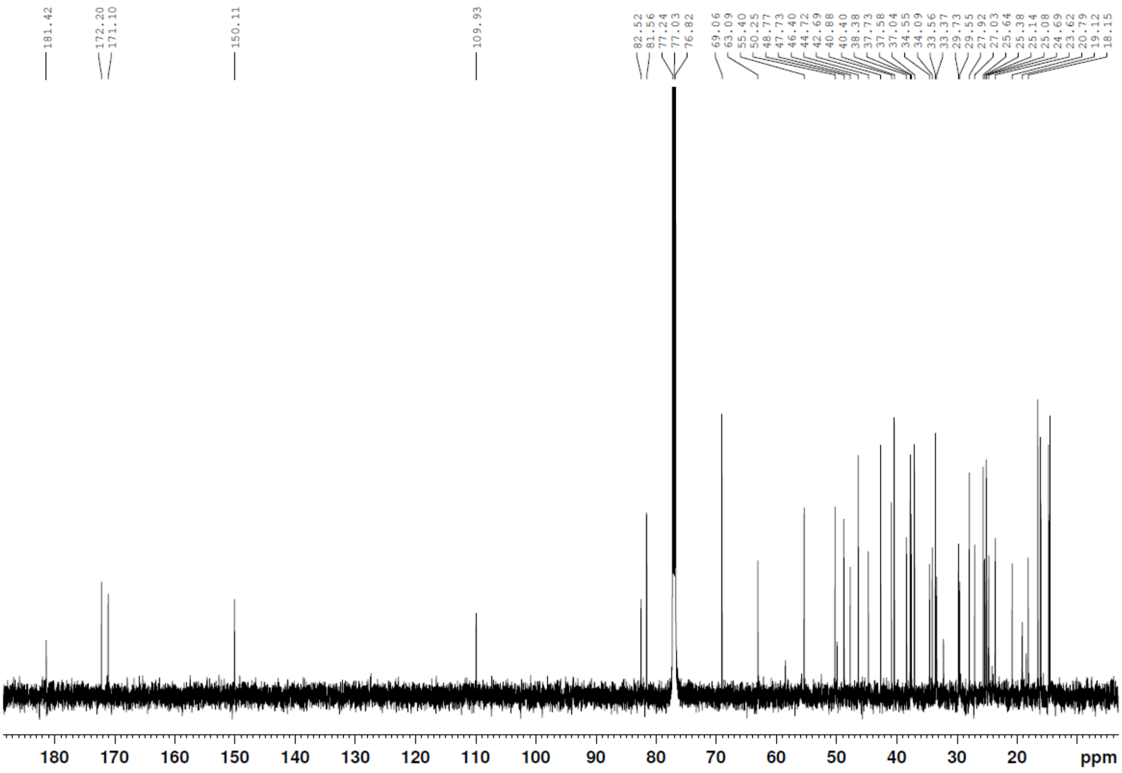


**Figure S37.** HRMS, compound **5b**

## Compound Spectrum List Report

**Analysis Info**

| | | | |
|---|---|---|---|
| Analysis Name | D:\Data\ECh 312.d | Acquisition Date | 3/4/2022 11:16:18 AM |
| Method | APCI_low_mass_negative.m | Operator | KM |
| Sample Name | 1-tolil | Instrument | impact II 1825265.10082 |
| Comment | | | |

**Acquisition Parameter**

| | | | | | |
|---|---|---|---|---|---|
| Source Type | APCI | Ion Polarity | Negative | Set Nebulizer | 2.0 Bar |
| Focus | Active | Set Capillary | 4000 V | Set Dry Heater | 200 °C |
| Scan Begin | 100 m/z | Set End Plate Offset | -500 V | Set Dry Gas | 4.0 l/min |
| Scan End | 1600 m/z | Set Charging Voltage | 2000 V | Set Divert Valve | Source |
| | | Set Corona | 15000 nA | Set APCI Heater | 450 °C |

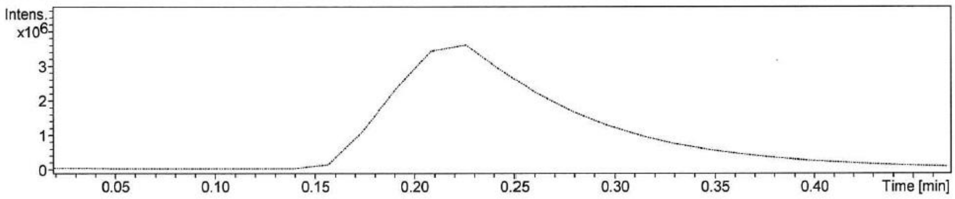


**-MS, 0.1-0.2min #8-10**

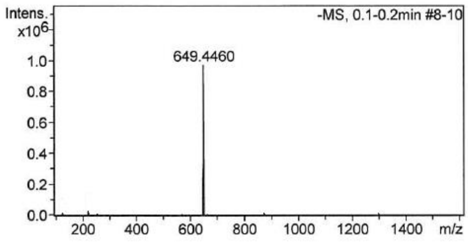


| # | m/z | Res. | S/N | I | I % | FWHM |
|---|---|---|---|---|---|---|
| 1 | 649.4460 | 21436 | 34623.2 | 972797 | 100.0 | 0.0303 |

**Figure S38.** $^{1}$H NMR, compound **5c**

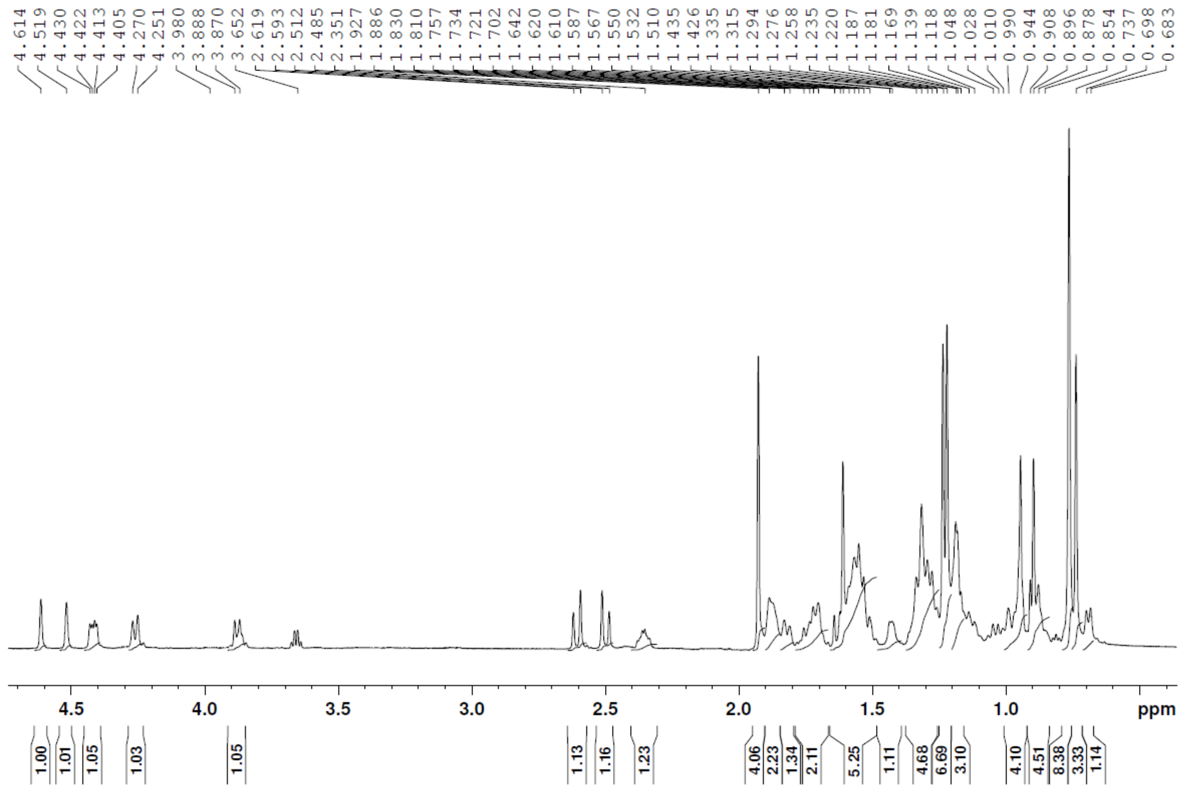


**Figure S39.** $^{13}$C NMR, compound **5c**

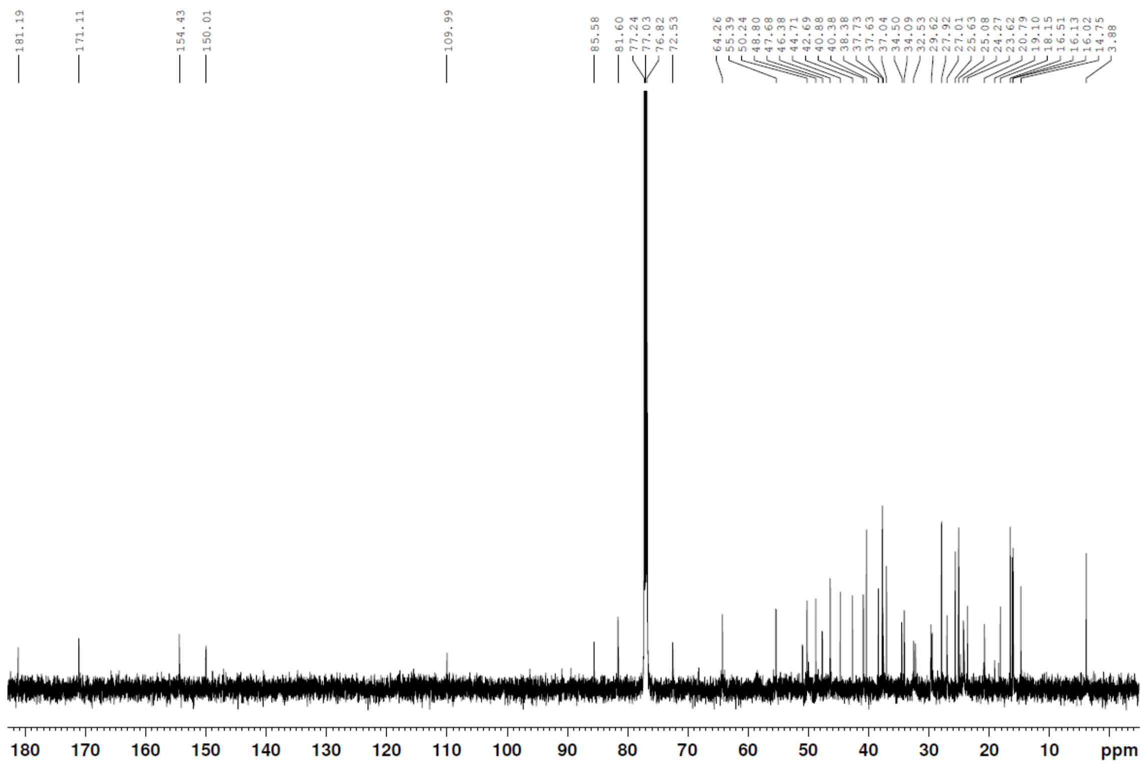

**Figure S40.** HRMS, compound **5c**

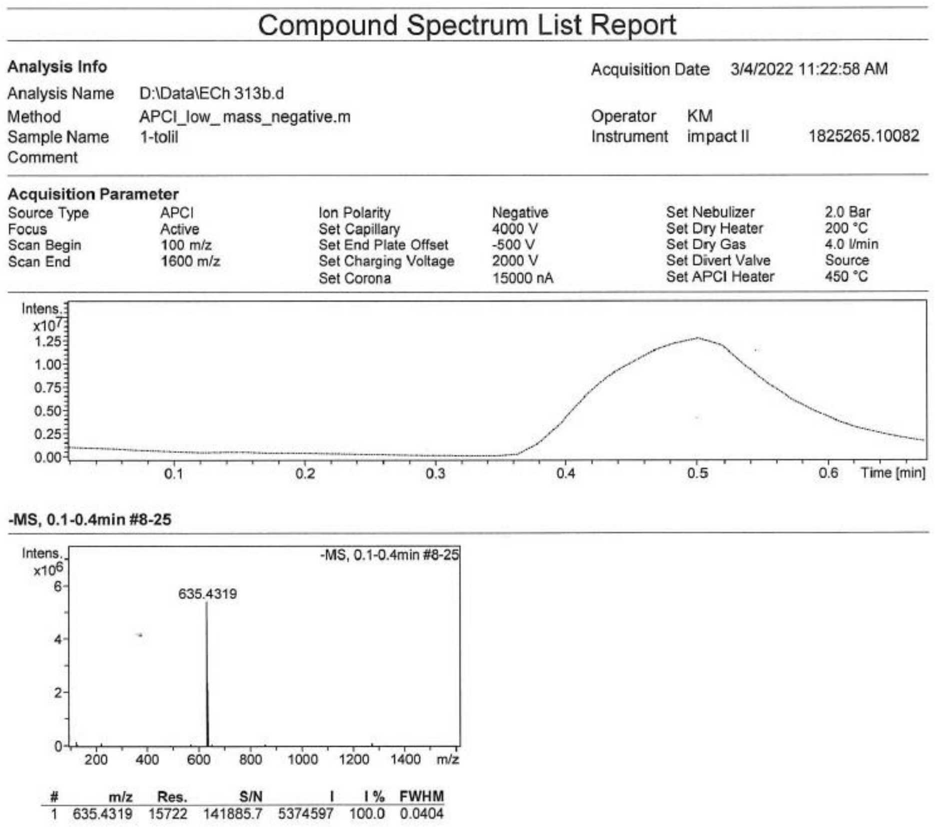


**Figure S41.** $^{1}$H NMR, compound **5d**

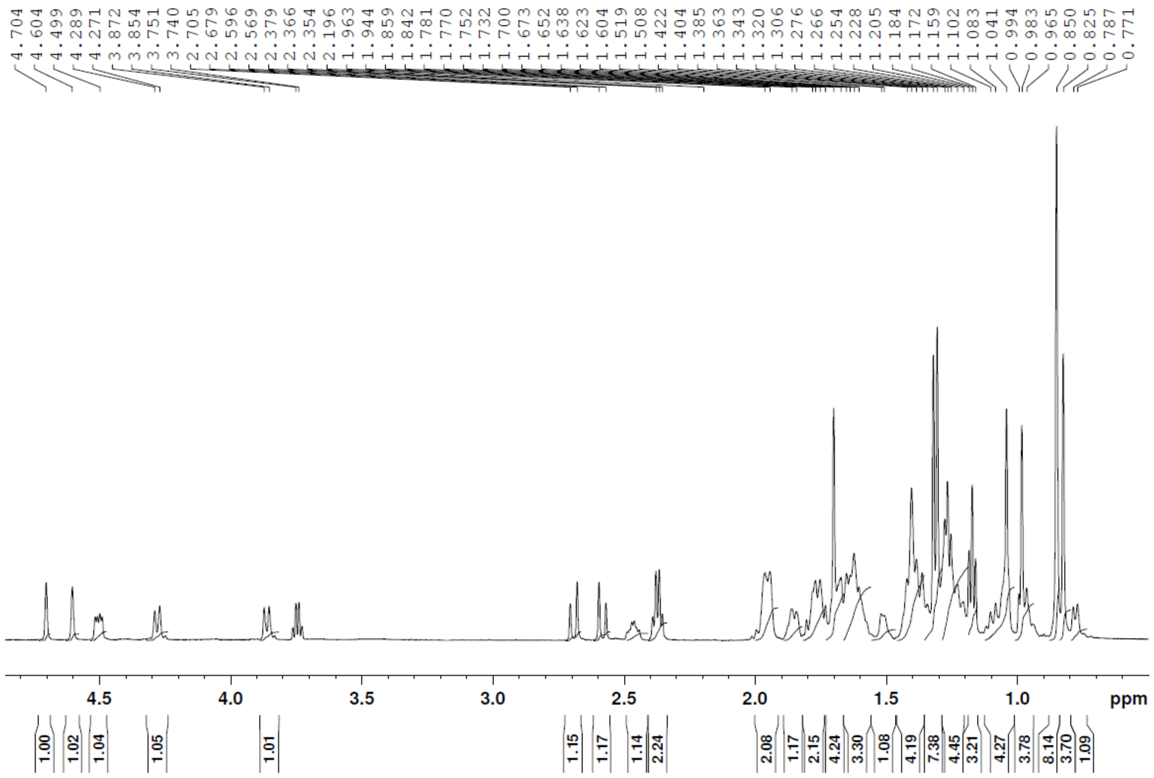

**Figure S42.** $^{13}$C NMR, compound **5d**

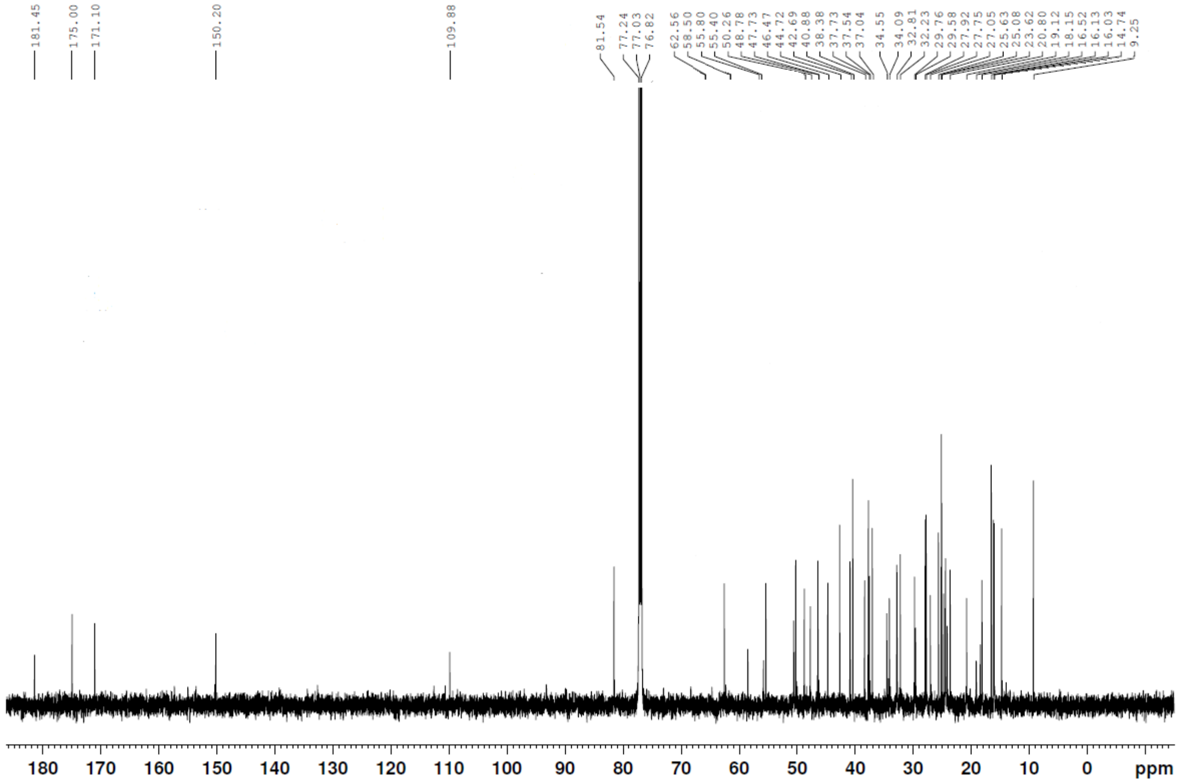


**Figure S43.** HRMS, compound **5d**

## Compound Spectrum List Report

**Analysis Info**

| | | | |
|---|---|---|---|
| Analysis Name | D:\Data\ECh 315.d | Acquisition Date | 3/4/2022 11:30:13 AM |
| Method | APCI_low_ mass_negative.m | Operator | KM |
| Sample Name | 1-tolil | Instrument | impact II 1825265.10082 |
| Comment | | | |

**Acquisition Parameter**

| | | | | | |
|---|---|---|---|---|---|
| Source Type | APCI | Ion Polarity | Negative | Set Nebulizer | 2.0 Bar |
| Focus | Active | Set Capillary | 4000 V | Set Dry Heater | 200 °C |
| Scan Begin | 100 m/z | Set End Plate Offset | -500 V | Set Dry Gas | 4.0 l/min |
| Scan End | 1600 m/z | Set Charging Voltage | 2000 V | Set Divert Valve | Source |
| | | Set Corona | 15000 nA | Set APCI Heater | 450 °C |

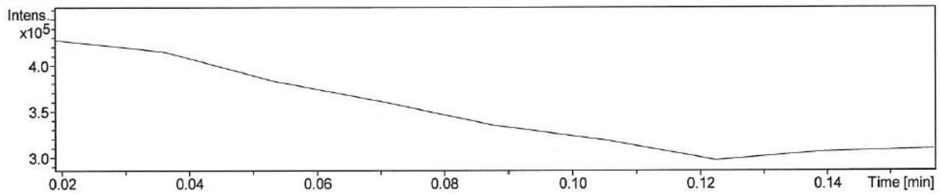


**-MS, 0.0-0.1min #2-3**

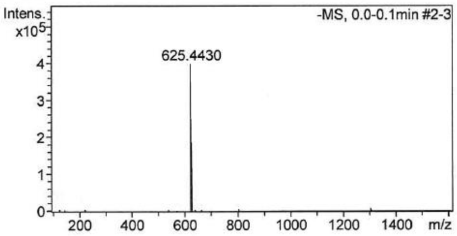


| # | m/z | Res. | S/N | I | I % | FWHM |
|---|---|---|---|---|---|---|
| 1 | 625.4430 | 14799 | 11871.7 | 399294 | 100.0 | 0.0423 |

**Figure S44.** $^{1}$H NMR, compound **6a**

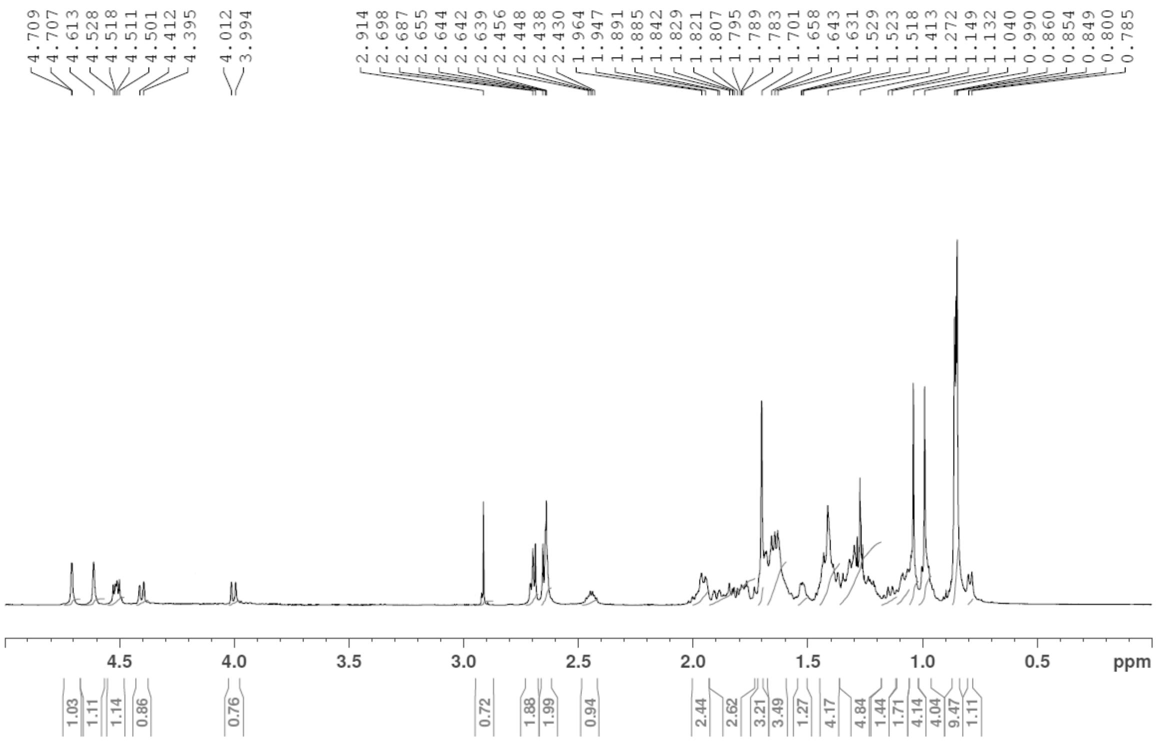


**Figure S45.** $^{13}$C NMR, compound **6a**

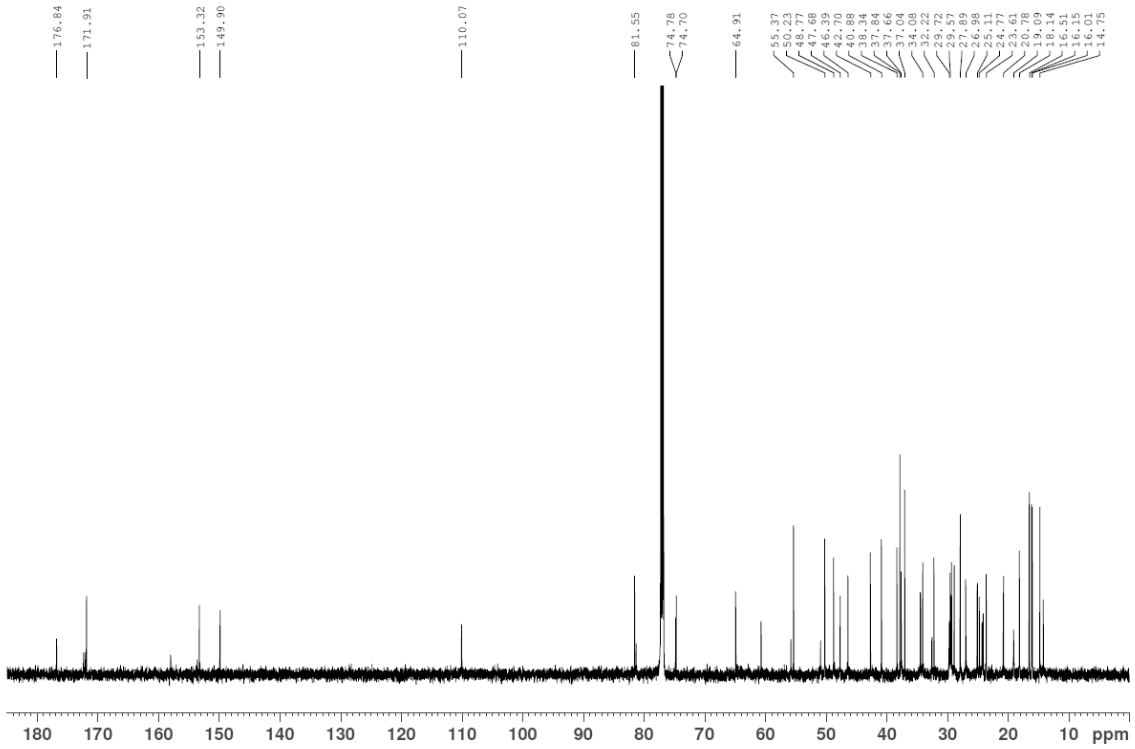

**Figure S46.** HRMS, compound **6a**

## Compound Spectrum List Report

**Analysis Info**

| | | | | |
|---|---|---|---|---|
| Analysis Name | D:\Data\ECh 319a.d | Acquisition Date | 3/4/2022 11:47:59 AM | |
| Method | APCI_low_ mass_negative.m | Operator | KM | |
| Sample Name | 1-tolil | Instrument | impact II | 1825265.10082 |
| Comment | | | | |

**Acquisition Parameter**

| | | | | | |
|---|---|---|---|---|---|
| Source Type | APCI | Ion Polarity | Negative | Set Nebulizer | 2.0 Bar |
| Focus | Active | Set Capillary | 4000 V | Set Dry Heater | 200 °C |
| Scan Begin | 100 m/z | Set End Plate Offset | -500 V | Set Dry Gas | 4.0 l/min |
| Scan End | 1600 m/z | Set Charging Voltage | 2000 V | Set Divert Valve | Source |
| | | Set Corona | 15000 nA | Set APCI Heater | 450 °C |

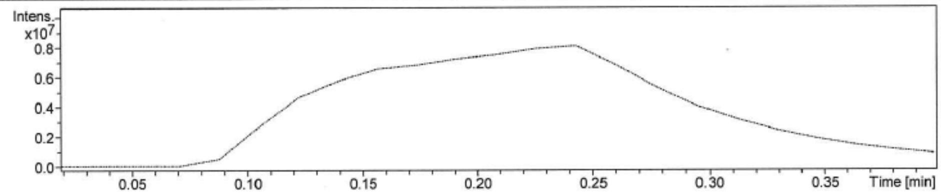


**-MS, 0.1-0.2min #6-9**

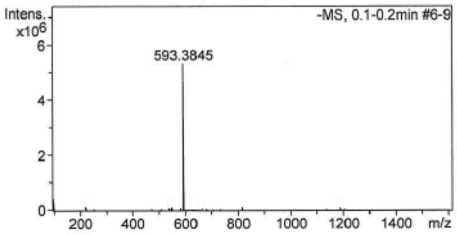


| # | m/z | Res. | S/N | I | I % | FWHM |
|---|---|---|---|---|---|---|
| 1 | 593.3845 | 29056 | 47946.2 | 5312838 | 100.0 | 0.0204 |

**Figure S47.** $^{1}$H NMR, compound **6b**

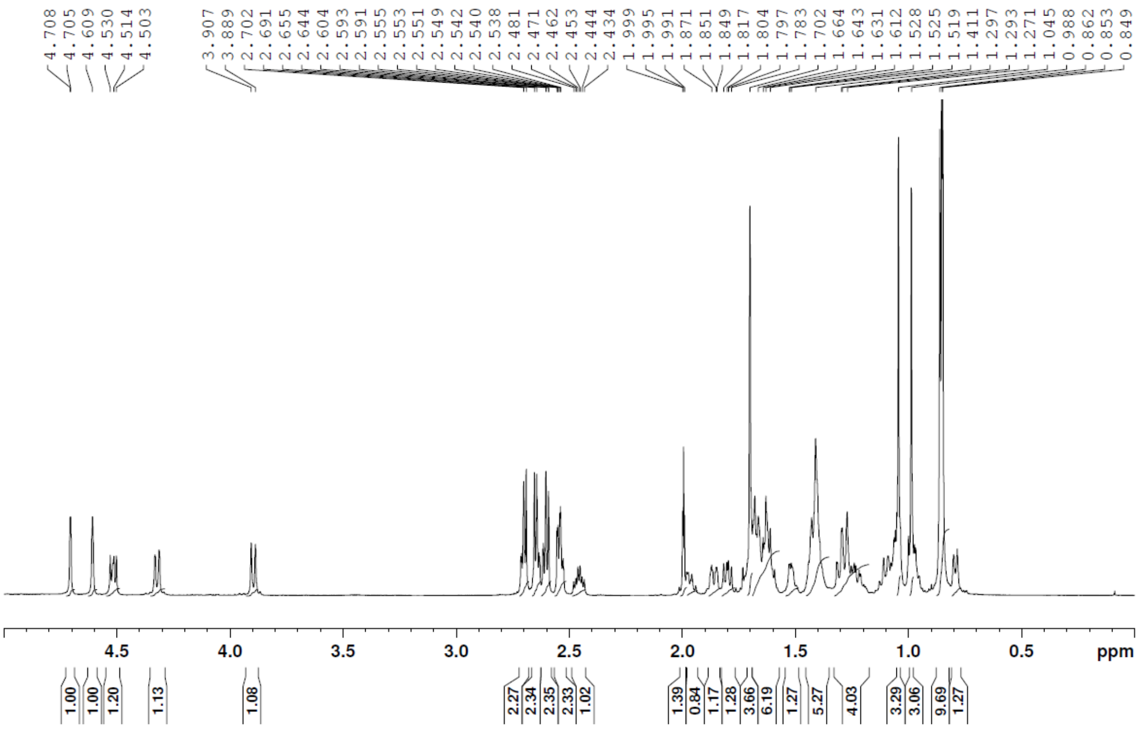

**Figure S48.** $^{13}$C NMR, compound **6b**

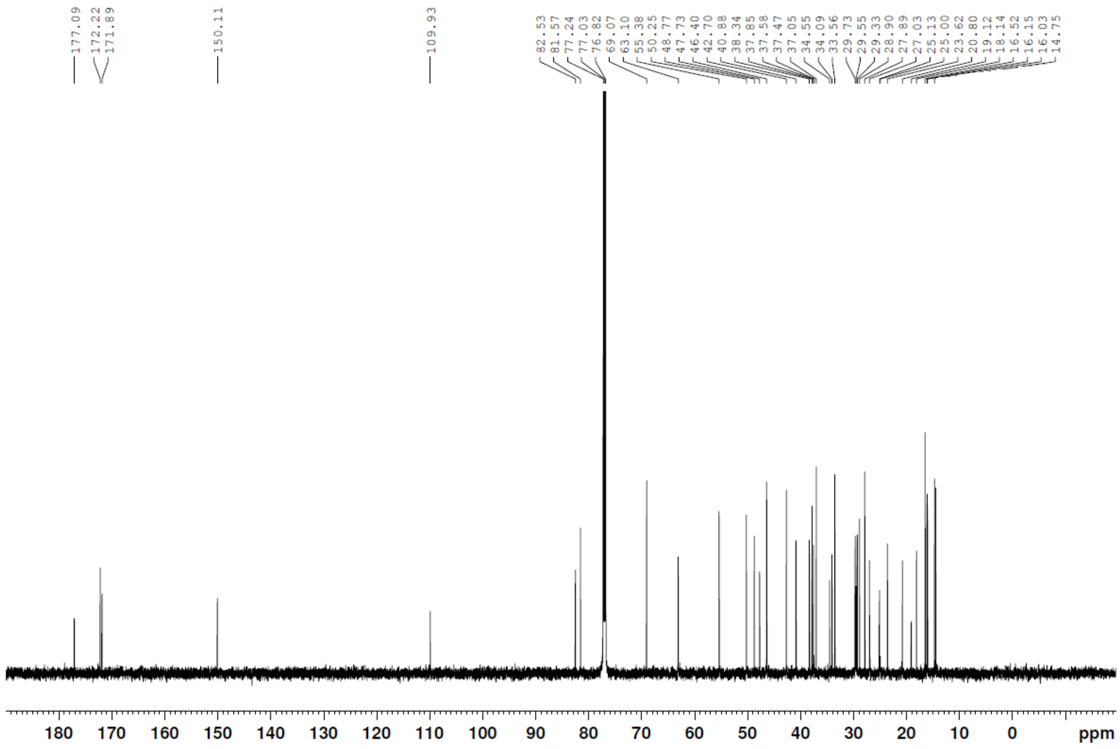


**Figure S49.** HRMS, compound **6b**

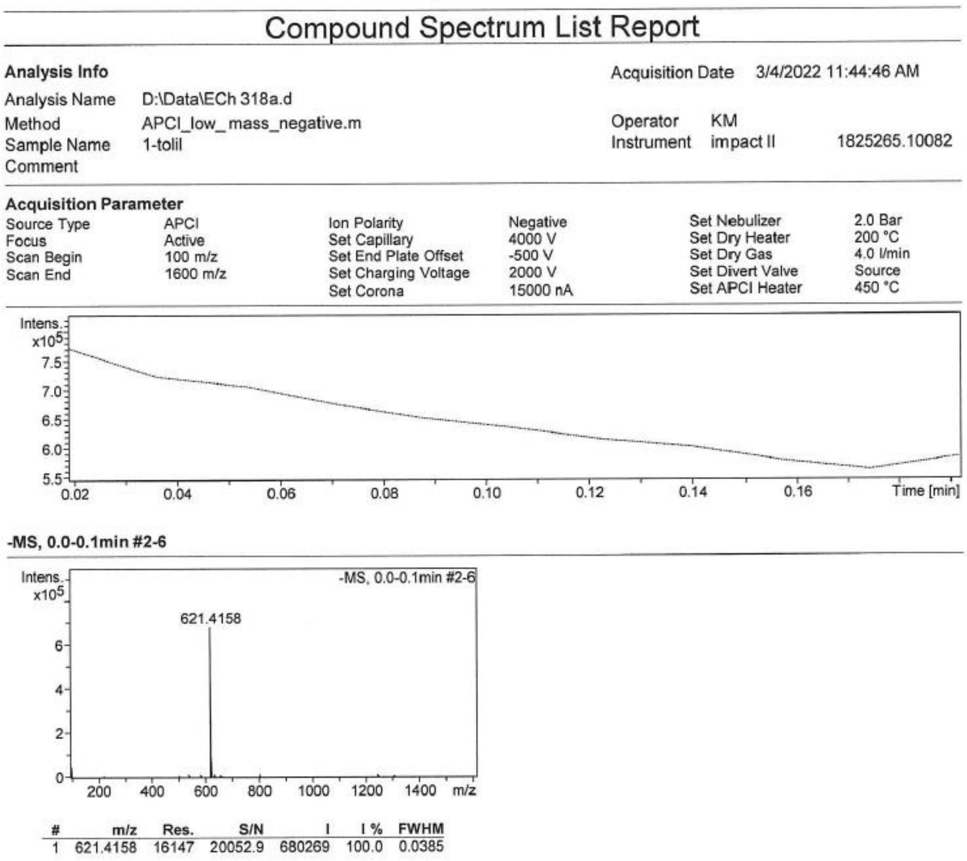

Compound Spectrum List Report

**Analysis Info**

| | | | |
|---|---|---|---|
| Analysis Name | D:\Data\ECh 318a.d | Acquisition Date | 3/4/2022 11:44:46 AM |
| Method | APCI_low_mass_negative.m | Operator | KM |
| Sample Name | 1-tolil | Instrument | impact II 1825265.10082 |
| Comment | | | |

**Acquisition Parameter**

| | | | | | |
|---|---|---|---|---|---|
| Source Type | APCI | Ion Polarity | Negative | Set Nebulizer | 2.0 Bar |
| Focus | Active | Set Capillary | 4000 V | Set Dry Heater | 200 °C |
| Scan Begin | 100 m/z | Set End Plate Offset | -500 V | Set Dry Gas | 4.0 l/min |
| Scan End | 1600 m/z | Set Charging Voltage | 2000 V | Set Divert Valve | Source |
| | | Set Corona | 15000 nA | Set APCI Heater | 450 °C |



**-MS, 0.0-0.1min #2-6**



| # | m/z | Res. | S/N | I | I % | FWHM |
|---|---|---|---|---|---|---|
| 1 | 621.4158 | 16147 | 20052.9 | 680269 | 100.0 | 0.0385 |
